# Synthetic studies with the brevicidine and laterocidine lipopeptide antibiotics including analogues with enhanced properties and *in vivo* efficacy†

**DOI:** 10.1039/d2sc00143h

**Published:** 2022-02-23

**Authors:** Karol Al Ayed, Ross D. Ballantine, Michael Hoekstra, Samantha J. Bann, Charlotte M. J. Wesseling, Alexander T. Bakker, Zheng Zhong, Yong-Xin Li, Nora C. Brüchle, Mario van der Stelt, Stephen A. Cochrane, Nathaniel I. Martin

**Affiliations:** Biological Chemistry Group, Institute of Biology, Leiden University Sylviusweg 72 2333 BE Leiden The Netherlands n.i.martin@biology.leidenuniv.nl; School of Chemistry and Chemical Engineering, Queen's University Belfast David Keir Building, Stranmillis Road BT9 5AG Belfast UK s.cochrane@qub.ac.uk; Molecular Physiology Group, Leiden Institute of Chemistry, Leiden University Einsteinweg 55 2333 CC Leiden The Netherlands; Department of Chemistry, The University of Hong Kong Pokfulam Road Hong Kong China

## Abstract

Brevicidine and laterocidine are two recently discovered lipopeptide antibiotics with promising antibacterial activity. Possessing a macrocyclic core, multiple positive charges, and a lipidated N-terminus, these lipopeptides exhibit potent and selective activity against Gram-negative pathogens, including polymyxin-resistant isolates. Given the low amounts of brevicidine and laterocidine accessible by fermentation of the producing microorganisms, synthetic routes to these lipopeptides present an attractive alternative. We here report the convenient solid-phase syntheses of both brevicidine and laterocidine and confirm their potent anti-Gram-negative activities. The synthetic routes developed also provide convenient access to novel structural analogues of both brevicidine and laterocidine that display improved hydrolytic stability while maintaining potent antibacterial activity in both *in vitro* assays and *in vivo* infection models.

## Abstract 2

Convenient solid-phase approaches are described for the synthesis of brevicidine and laterocidine. Also reported are novel analogues including a laterocidine variant with enhanced hydrolytic stability and potent *in vivo* antibacterial activity.

## Graphical abstract

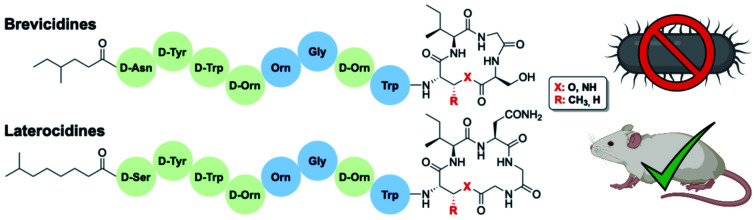

## Introduction

The accelerated appearance of multi-drug resistant bacterial pathogens has led to the worrying speculation that society may soon face a “post-antibiotic” era.^1–3^ The gravity of the antimicrobial resistance (AMR) crisis is most clearly reflected by the spread of the “ESKAPE” pathogens (*E. faecium*, *S. aureus*, *K. pneumoniae*, *A. baumannii*, *P. aeruginosa*, and Enterobacter species), a group of organisms that are increasingly difficult or impossible to treat with conventional antibiotics. Globally, deaths due to infections with drug-resistant bacteria are nearing one million per year.^4^ Even more worrying are recent projections suggesting that by the year 2050 the number of AMR associated deaths will grow to a staggering ten million per year.^4^

To prioritize the greatest threats currently associated with AMR, the World Health Organization (WHO) recently published its list of priority pathogens.^5^ Among these pathogens, it is exclusively the Gram-negative members of the ESKAPE family that are labeled as “critical”, the highest threat level on the WHO list. This is due to the rapidly accelerating rise in antibiotic resistance among Acinetobacter, Pseudomonas, and various Enterobacteriaceae (including Klebsiella and *Escherichia coli*) causing severe and often deadly bloodstream and pulmonary infections. The AMR threat underscores the importance of pursuing new strategies in discovering and developing the antibiotics of the future.

**Fig. 1:**
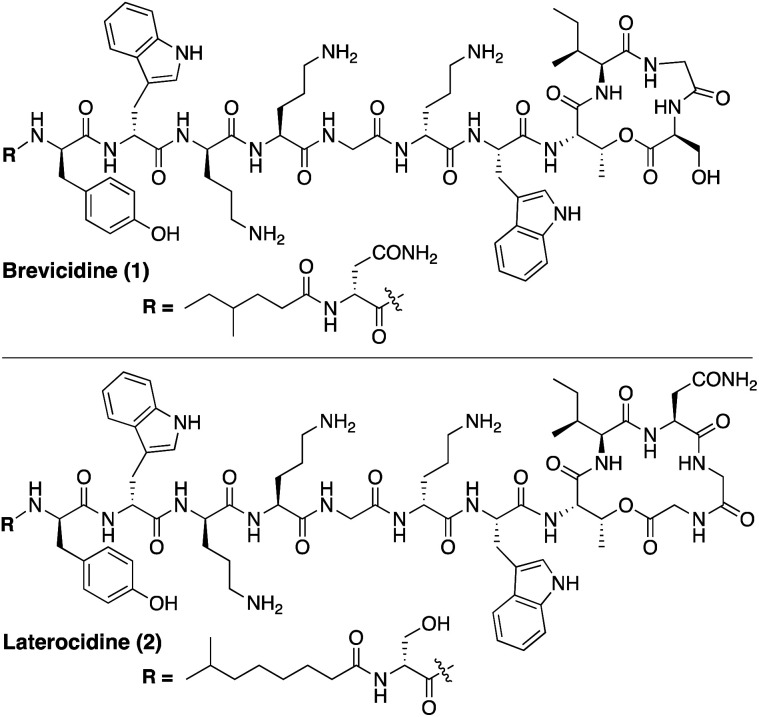
Structures of brevicidine (1) and laterocidine (2).

Using a biosynthetic gene cluster mining strategy, Li and co-workers recently reported the discovery of a promising new class of macrocyclic lipopeptides termed the brevicidines and laterocidines (Fig. 1).^6^ Produced by strains of *Brevibacillus laterosporus*, brevicidine and laterocidine specifically kill all Gram-negative members of the ESKAPE family, including drug-resistant strains. The antibacterial activity of these lipopeptides is promising, with MIC values comparable to the polymyxins, the only class of lipopeptides presently used in the clinical treatment of serious Gram-negative infections. Of particular note is the finding that brevicidine and laterocidine effectively kill pathogenic strains featuring the recently reported MCR-type polymyxin resistance mechanism.^6^ Furthermore, the initial report describing the discovery of brevicidine and laterocidine also indicates that these lipopeptide antibiotics show little propensity to induce resistance and have low toxicity towards mammalian cells.^6^

While brevicidine and laterocidine are promising new anti-Gram-negative antibiotics, both compounds are difficult to isolate in significant quantities from natural sources, presenting a major obstacle to investigating their full potential. Brevicidine and laterocidine can be obtained by fermentation of the producing microorganisms, however this labour-intensive process provides limited amounts of material (sub-milligram-per-litre yields in the case of laterocidine).^6^ For this reason, reliable synthetic routes to these lipopeptides present an attractive alternative as a means of providing larger amounts of material for both clinical evaluation and mechanistic studies.^7,8^ Herein we report the total syntheses of brevicidine and laterocidine by solid-phase peptide synthesis (SPPS).^9^ The synthetic compounds and natural products have identical ^1^H-NMR spectra, RP-HPLC retention times, and antibacterial activities. Using the same synthetic approach, the enantiomers of brevicidine and laterocidine were also prepared to probe the role of stereochemistry in the antibacterial mechanism of these unique lipopeptides. Furthermore, novel analogues wherein the ester moiety of the peptide macrocycle was replaced by an amide linkage, were prepared and their stability and antibacterial activities assessed both *in vitro* and *in vivo*.

## Results and discussion

Brevicidine and laterocidine share several structural features, including a C-terminal ester-linked macrocycle of 4- or 5-amino acids, a number of conserved residues including three positively charged ornithines, and a lipidated N-terminus. It was previously demonstrated that the macrocycle is necessary for antibacterial activity of these peptides.^6^ Key to the syntheses of both brevicidine and laterocidine was therefore development of a reliable approach for the introduction of the macrocycle formed *via* an ester linkage between the C-terminal carboxylate and the corresponding threonine side chain hydroxyl group. For both brevicidine and laterocidine, convenient solid phase approaches were developed that allowed on-resin formation of the key macrocycle and installation of all other amino acids.

**Scheme 1:**
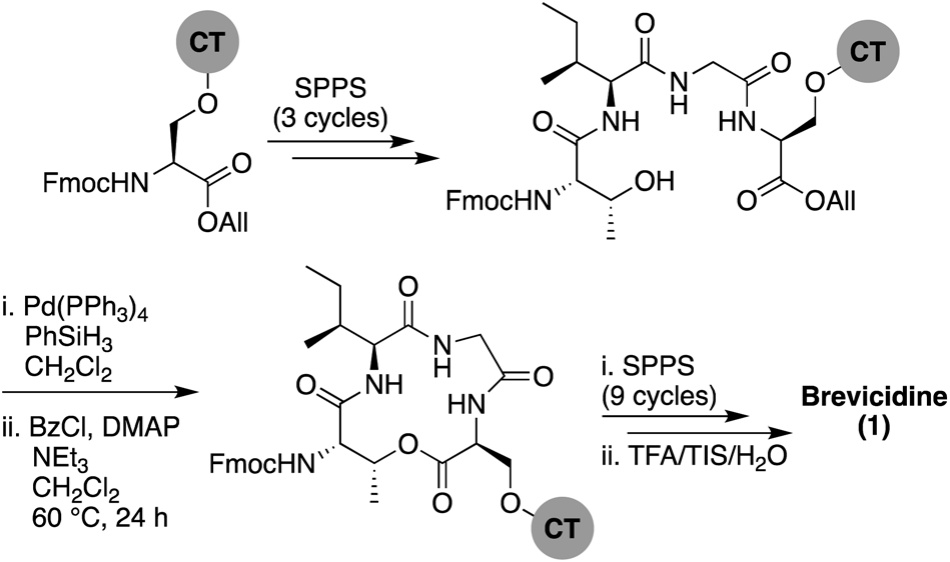
Total SPPS of brevicidine (1). CT = 2-chlorotrityl resin.

In approaching the synthesis of brevicidine, we initially investigated a strategy starting from Gly11 loaded on 2-chlorotrityl resin (CT) to generate a linear peptide that would subsequently be cyclized in solution. We envisaged installation of the required Thr9–Ser12 linkage as a preformed, ester-linked dipeptide. However, while incorporation of the Thr9–Ser12 unit was achieved, further elongation of the peptide failed due to an O → N acyl shift that occurred upon removal of the Thr9 Fmoc group (see ESI Scheme S1†). As an alternative, we next examined formation of the macrocycle at an earlier stage to assess whether the ester linkage might be more stable when contained in the more conformationally restricted ring system. To implement this approach, Fmoc-Ser-OAll was loaded on to CT resin *via* its free side-chain hydroxyl group. Notably, the initial conditions used (1 h, RT) resulted in a lower loading than required (0.07 mmol g^−1^), so the reaction time was extended to 24 h along with heating at 45 °C, resulting in an improved loading of 0.13 mmol g^−1^. Resin-bound Fmoc-Ser-OAll was then extended to the tetrapeptide using standard Fmoc-SPPS (Scheme 1). At this stage, the C-terminal allyl ester was cleanly removed on resin using Pd(PPh_3_)_4_/PhSiH_3_ in CH_2_Cl_2_. Subsequent closure of the macrolactone was then investigated using different coupling conditions. Cyclization of the on-resin tetrapeptide proved to be refractory to both the use of 1-ethyl-3-(3-dimethylaminopropyl) carbodiimide (EDC) and diisopropylcarbodiimide (DIC), with only starting material being obtained after 48 h. To circumvent this lack of reactivity, we next attempted a modified Yamaguchi esterification, utilizing benzoyl chloride as the coupling reagent, along with a 24 h reaction time run at RT. Although only a small amount of the desired cyclic product was obtained using these conditions, increasing the reaction temperature to 60 °C resulted in near complete conversion. Building from the successful formation of the resin-bound tetrapeptide macrolactone, all that remained was to extend the peptide from the N-terminal threonine residue with SPPS.

It was thought best to proceed with caution at the initial Fmoc deprotection to avoid unwanted O → N acyl migration. Therefore, less aggressive Fmoc deprotection conditions (10% piperidine in DMF) were used in the first deprotection. Gratifyingly, we did not detect any O → N acyl migration, even using standard Fmoc deprotection conditions, validating our hypothesis that preforming the C-terminal macrocycle would overcome this issue. The remainder of the peptide was constructed without incident along with coupling of the N-terminal 4-methylhexanoic acid. Following global deprotection and resin cleavage and purification by RP-HPLC, synthetic brevicidine was obtained in an overall yield of 9% over 28 steps.

**Scheme 2:**
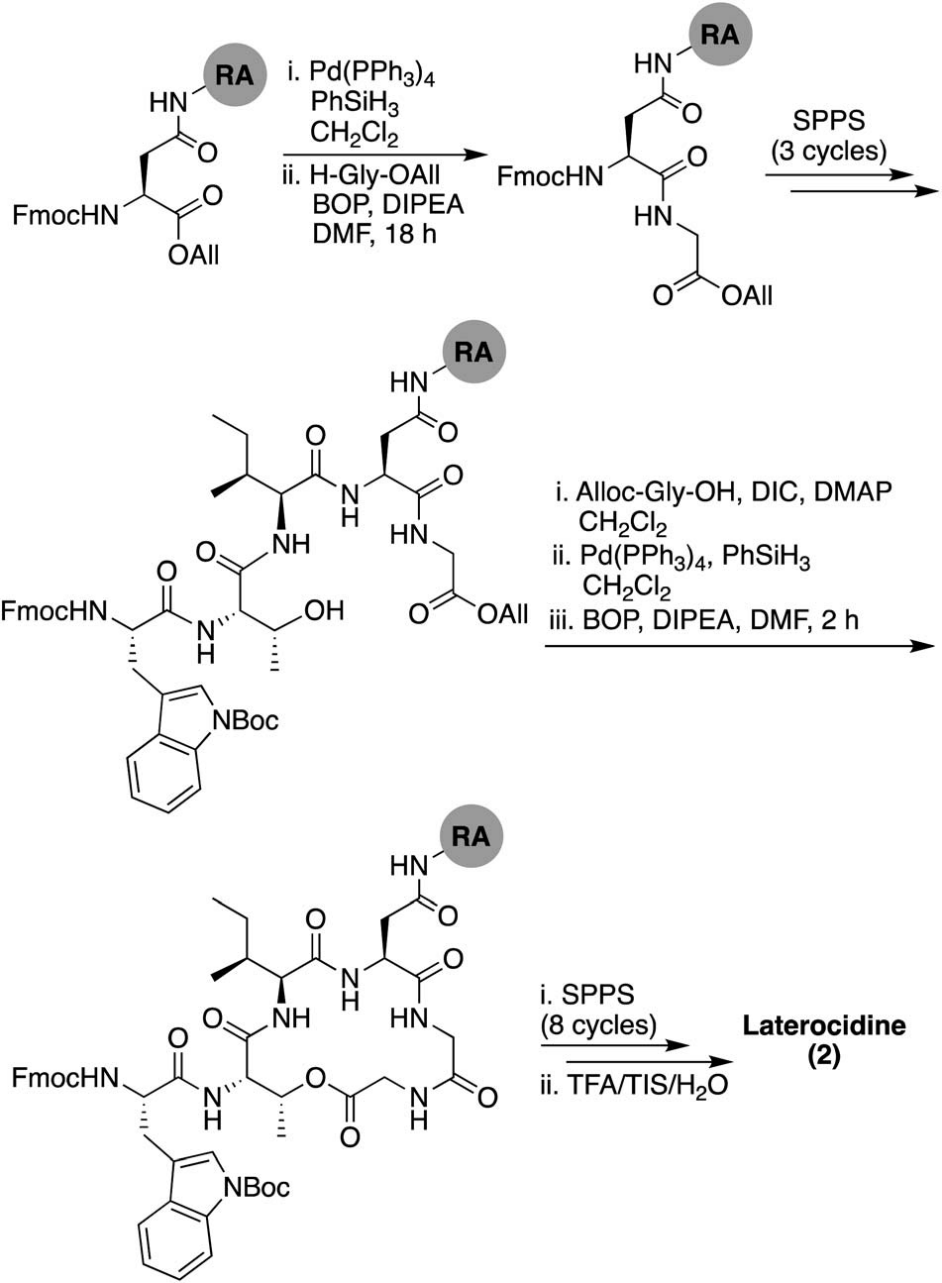
Total SPPS of laterocidine (2). RA = Rink amide resin.

The synthetic strategy initially pursued for the preparation of laterocidine was inspired by the successful route developed for brevicidine (see ESI Scheme S2†). Unfortunately, formation of the macrolactone proved refractory towards a variety of conditions, including the modified Yamaguchi esterification, with all failing to provide the desired product in appreciable yield. We ascribe this difficulty in ester formation to the larger five-amino-acid macrocycle found in laterocidine *versus* the four-amino-acid ring found in brevicidine. As an alternative, we next investigated the possibility of closing the macrocycle *via* amide bond formation between Gly12 and Gly13 (Scheme 2). Following allyl ester cleavage of Fmoc-Asp-OAll loaded Rink amide (RA) resin, an allyl ester protected Gly was coupled after which the peptide was built out to Trp8. The ester linkage between the free Thr9 side chain hydroxyl and the Gly13 C-terminal carboxylate was successfully introduced by coupling Alloc-Gly-OH using an on-resin Steglich esterification approach inspired by the Albericio group's synthesis of pipecolidepsin A.^10^ Following removal of the allyl and Alloc protecting groups, a BOP/DIPEA mediated macrolactamization resulted in the clean formation of the macrocyclic product. From there the peptide was completed using standard Fmoc-SPPS conditions, including N-terminal lipidation with isopelargonic acid. Following cleavage from resin and global deprotection, the crude lipopeptide was subsequently purified using RP-HPLC, yielding laterocidine in 2% purified yield over 29 steps.

Our route to brevicidine and laterocidine was originally disclosed in preprint form (uploaded to ChemRxiv on 10 Jan 2021).^9^ Subsequently, Hermant and co-workers reported a different synthetic strategy also providing access to brevicidine and laterocidine.^11^ As described above, our syntheses are performed entirely on solid support, wherein a side-chain residue is immobilized (Ser 12 for brevicidine and Asn11 for laterocidine) allowing for on resin formation of the peptide macrocycle. Hermant and co-workers opted for an alternate approach combining solid-phase and solution phase synthesis, wherein linear peptide precursors were assembled on resin followed by macrolactamization in solution.

**Fig. 2:**
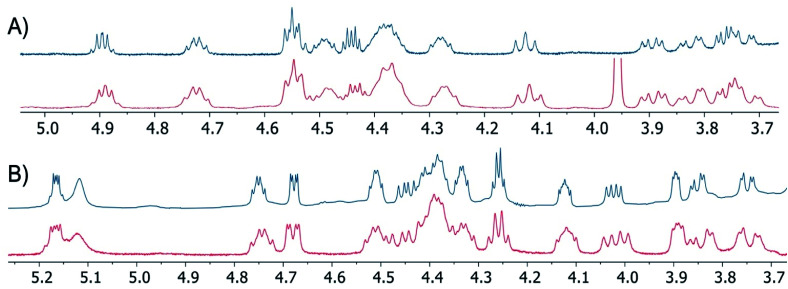
Overlaid portions of ^1^H-NMR spectra obtained for synthetic lipopeptides (blue traces) and previously published spectra (red traces) corresponding to: (A) brevicidine (1) and (B) laterocidine (2). The peak at *ca.* 3.96 ppm in the published spectrum of brevicidine is attributed to an impurity not present in the synthetic material. Spectra recorded in DMSO-*d*_6_ at RT. Full ^1^H-NMR spectra provided in the ESI.†

To confirm the equivalency of the synthetic and natural lipopeptides, their ^1^H-NMR spectra were compared with published data for the natural products, revealing them to be indistinguishable (Fig. 2, also see ESI Fig. S1–S4†). In addition, LC-MS/MS analysis of the synthetic lipopeptides and comparison to the natural products further verified their identity (see ESI Fig. S5†). Antibacterial assays were also performed against a range of Gram-negative bacteria, which confirmed that synthetic brevicidine and laterocidine possess the same activity profile as the natural products (Table 1). To confirm that the antibacterial activity of brevicidine and laterocidine is intrinsically dependent on the chirality of the molecules, we synthesized the enantiomeric forms of both lipopeptides. The syntheses of *ent*-brevicidine (*ent-*1) and *ent*-laterocidine (*ent*-2) were achieved by following the same routes developed for the natural lipopeptides, but employing the corresponding mirror image amino acid building blocks (see ESI Schemes S3 and S4†). Of note, similar mirror-image strategies have been used to characterize the stereochemical aspects in the antibacterial mechanisms of other peptide antibiotics.^12–16^ In cases where an achiral target is implicated, the enantiomeric peptide antibiotic typically shows activity on par with the natural product, as in the case of bacitracin, which targets undecaprenyl pyrophosphate (C55-PP),^12^ and laspartomycin, which targets undecaprenyl phosphate (C55-P).^13^ Conversely, in the case of tridecaptin A1, which targets the chiral cell wall precursor lipid II,^14^ thanatin, which targets the LptA and LptD proteins involved in LPS biosynthesis,^15^ and daptomycin, which targets phosphatidylglycerol,^16^ the activities of the corresponding enantiomers is significantly reduced. In their initial investigations with brevicidine and laterocidine, Li and co-workers found that addition of exogenous lipopolysaccharides (LPS) strongly antagonized the activity of both peptides, suggesting an interaction with LPS as part of the mechanism of action.^6^ Given the inherent chirality of LPS, we hypothesized that *ent*-brevicidine and *ent*-laterocidine might exhibit decreased antibacterial activities relative to the natural products in the event that stereochemically defined binding interactions with LPS, rather than nonspecific electrostatic interactions, are central to the working mechanism of the lipopeptides. As confirmation of this hypothesis, the antibacterial activities of *ent*-brevicidine and *ent*-laterocidine were found to be consistently lower than the natural products, with the MIC values ranging from 8- to 32-fold higher (Table 1). Interestingly, the antibacterial activities of both laterocidine and *ent*-laterocidine were found to be antagonized upon addition of LPS (see ESI Fig. S6†). In the presence of LPS, the activities of laterocidine and *ent*-laterocidine decrease by 8-fold or more, indicating that the interaction with LPS may be driven by nonspecific electrostatic interactions. This finding suggest another chiral biomolecular target might be involved in the mechanism of action associated with these lipopeptides. Studies are underway to identify this target.

**Table 1:** *In vitro* minimum inhibitory concentrations (MICs) of peptide analogues determined using microbroth-dilution assays^a^

	Brev (1)	*ent-*Brev (*ent*-1)	Ser9-Brev (3)	Dap9-Brev (5)	MeDap9-Brev (7)	Lat (2)	*ent-*Lat (*ent*-2)	Ser9-Lat (4)	Dap9-Lat (6)	MeDap9-Lat (8)	Col	PolyB
*Ec* ATCC 25922	1–2	16	4	8–16	16	1	8	0.5	1	1	0.25–0.5	0.5
*Ec* ATCC 25922 MCR-1	2	16	4	16	8	2	8–16	0.5	1–2	1	2–4	2–4
*Ec* MCR-1	1–2	16	2	8–16	8	0.5	8–16	0.5	1	0.5	2–4	2–4
*Ec* EQAS MCR-2	2	16–32	4–8	8–16	8–16	1	8–16	0.5–1	1	0.5	4	4
*Kp* ATCC 11228	1–2	32	2	16–32	8	2	16	1–2	2	1–2	<0.25	<0.25
*Kp* ATCC 13883	1	32	2	32	8	1	16	1–2	2	1–2	0.25	0.25
*Kp* 2048	1–2	32	4	16–32	8–16	2	16	2	2	2	0.5–1	0.5–1
*Kp* JS-123	1	8	2	8	4–8	0.5	4	0.5	0.5	0.5	<0.25	<0.25
*Ab* ATCC 17961	2–4	32	0.5–1	4	8–16	0.5	4	4	4	2	<0.25	<0.25
*Ab* ATCC 17978	4	16–32	2	16	16	0.5	4	2	4	2	<0.25	<0.25
*Ab* 2018-006	4	8–16	2	8	16	2	4	4	4	4	<0.25	<0.25
*Ab* MDR	4	16	1–2	8	16	1	4–8	2	4	4	<0.25	<0.25
*Pa* ATCC 27853	≤0.5	16	1	8	2–4	≤0.5	8	0.5–1	0.5–1	0.5	<0.25	<0.25
*Pa* PAO1	2	32	2–4	16–32	4	1	16	2	1–2	2	0.25–0.5	0.25–0.5
*Pa* NRZ-03961	2	32	4	16–32	8	1–2	16	1–2	2	2	0.25–0.5	0.25–0.5
*Pa* M-120	2–4	16	2	16	4	1	16	1	1–2	1	<0.25	<0.25
*Sa* USA300	64	>64	>64	>64	>64	64	64	>64	>64	32	>64	64

a *Ec* = *E. coli*, *Kp* = *K. pneumoniae*, *Ab* = *A. baumannii*, Pa = *P. aeruginosa*, *Sa* = *S. aureus*, Col = colistin, PolyB = polymyxin B. MICs reported in μg mL^−1^.

**Fig. 3:**
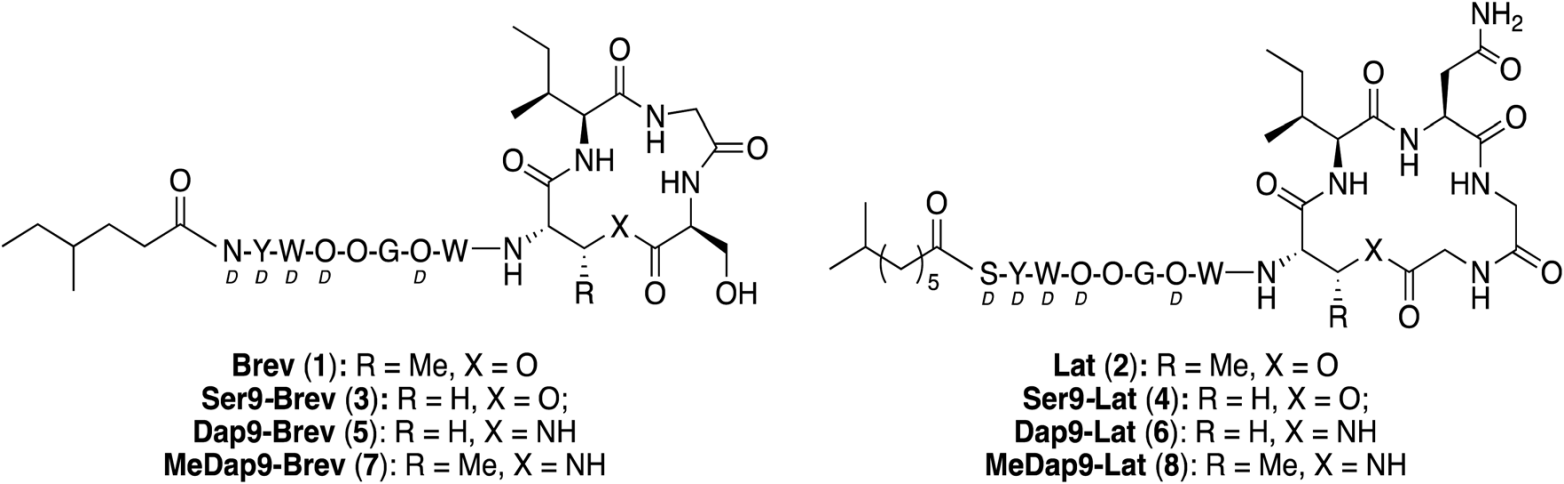
Brevicidine and laterocidine analogues containing modified macrocycles. Non-ring amino acids shown as one-letter codes. d-Amino acids labelled *d*.

To probe the role of the Thr9 side chain methyl group on the conformational requirements of the brevicidine and laterocidine macrocycles, analogues were synthesized in which the Thr9 residue was substituted for Ser (Fig. 3). The synthetic route used in preparing Ser9 analogues 3 and 4 was essentially the same as for the natural products (see ESI Schemes S5 and S6†), although it was found that more strictly anhydrous conditions were necessary to prevent product degradation during the cyclization step. The antibacterial activities of the Ser9 analogues were similar to the natural products, suggesting the β-methyl group in Thr9 does not play a crucial role in dictating the biologically active conformation of either peptide.

**Scheme 3:**
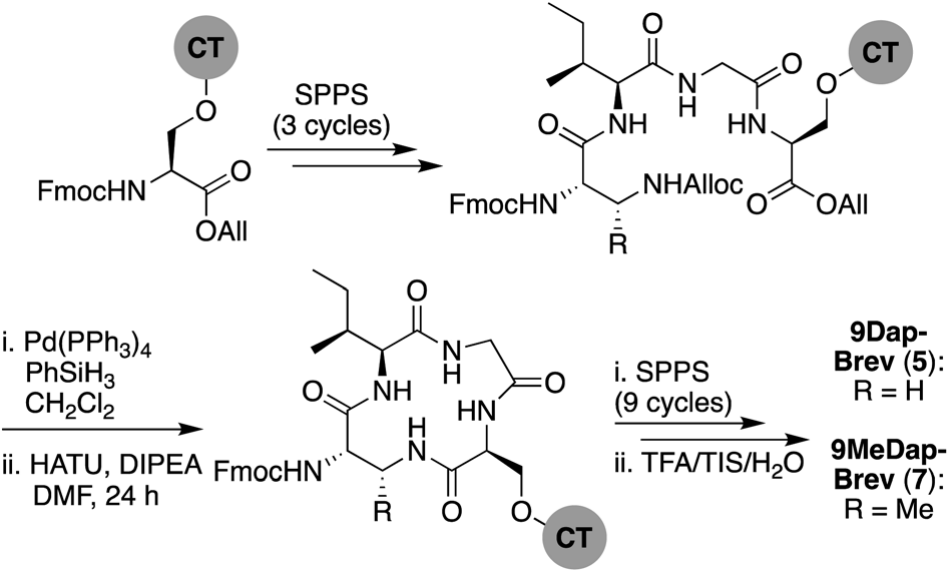
Total SPPS of Dap9-Brev (5) and MeDap9-Brev (7).

**Scheme 4:**
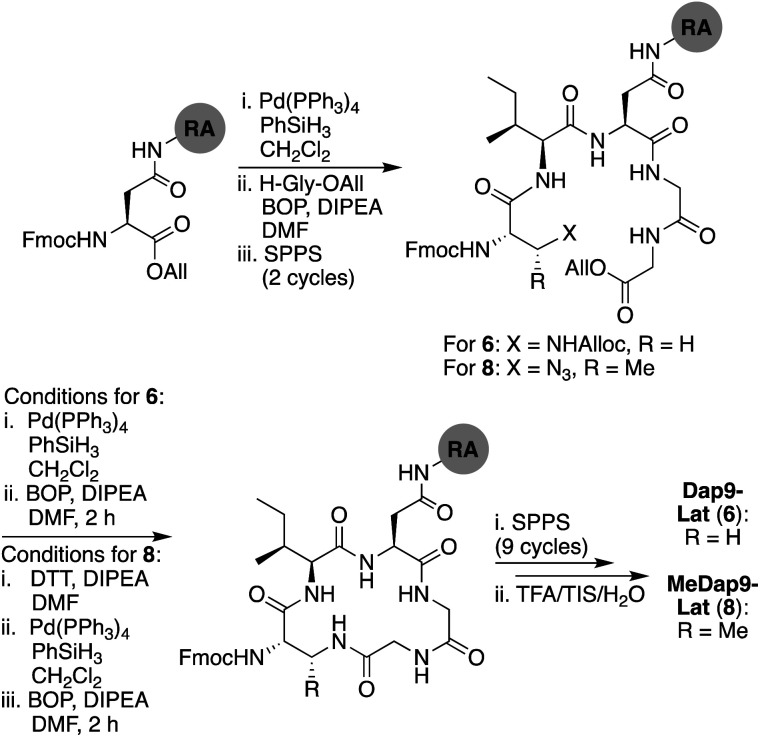
Total SPPS of Dap9-Lat (6) and MeDap9-Lat (8).

We next explored the effect of replacing the ester linkage between the Thr9 side chain and the C-terminus with the corresponding amide. For several macrocyclic depsipeptide antibiotics, it has been shown that such amide for ester substitutions can have a positive effect on hydrolytic stability, as in the case of analogues of ramoplanin,^17^ fusaricidin,^18^ daptomycin,^19^ and fengycin,^20^ Additionally, from a synthetic perspective, macrocyclic ring closure *via* formation of an amide linkage can be more facile than formation of the corresponding macrolactone due to the enhanced reactivity of amines over alcohols. To this end, amide analogues of brevicidine and laterocidine were prepared, and their hydrolytic stability and antibacterial activities assessed. For both lipopeptides, we examined the effect of replacing Thr9 with either (*S*)-2,3-diaminopropanoic acid (Dap) or (2*S*,3*R*)-2,3-diaminobutanoic acid (MeDap) to yield analogues 5–8 (Fig. 3). The synthesis of Dap9-Brev (5) started from CT resin loaded with Fmoc-Ser-OAll *via* its side-chain hydroxyl group (Scheme 3). Iterative Fmoc-SPPS was then used to construct the linear protected on-resin tetrapeptide incorporating Fmoc-Dap(Alloc)-OH. Both the allyl and Alloc groups were removed in a single deprotection with Pd(PPh_3_)_4_/PhSiH_3_ in CH_2_Cl_2_. With the amine of Dap9 and the C-terminal carboxylic acid of the peptide liberated, an overnight macrolactamization was effected using HATU/DIPEA. Fmoc-SPPS was continued to complete the linear peptide backbone, followed by capping with 4-methylhexanoic acid. Finally, a global cleavage of the peptide from resin and subsequent purification by RP-HPLC yielded Dap9-Brev (5) in a 13% yield over 28 steps. The synthesis of the MeDap9-Brev (7) followed a similar strategy, with the exception that the corresponding Fmoc-MeDap(Alloc)-OH building block was incorporated at position 9. The antibacterial activities of brevicidine amide analogues 5 and 7 were evaluated, which in both cases revealed a significant loss of activity relative to brevicidine itself (Table 1). This reduced activity may be due to a loss of flexibility in the brevicidine macrocycle, caused by the ester to amide substitution preventing it from accessing its fully active conformation. In addition, the serum stability of Dap9-Brev (5) was assessed, revealing it to be much more stable than brevicidine (see ESI Fig. S7†). Dap9-Lat (6) was prepared from Fmoc-Asp-OAll loaded Rink amide resin (Scheme 4). Treatment with Pd(PPh_3_)_4_/PhSiH_3_ in CH_2_Cl_2_, followed by activation and coupling to H_2_N-Gly-Gly-OAll, yielded an on-resin tripeptide, which was then elongated to the macrocycle precursor. After removal of the Alloc and allyl groups, on-resin macrocyclization was effected using BOP/DIPEA. The peptide was then completed using standard Fmoc-SPPS conditions, including N-terminal lipidation with isopelargonic acid. After cleavage from the resin, and global deprotection, the crude lipopeptide was purified using RP-HPLC, yielding Dap9-Lat (6) in 3% overall yield over 27 steps. MeDap9-Lat (8) was synthesized following essentially the same on-resin protocol used for 6, with the exception that the MeDap residue was installed as the corresponding azido species and reduced on-resin before the macrolactamization step (this strategy was also attempted for MeDap9-Brev (7) but the on-resin azide reduction step caused peptide decomposition).

In addition to the total SPPS of Dap9-Lat (6), we also investigated an operationally more straightforward approach, wherein the amide linked macrocycle was formed in solution at the end of the synthesis (see ESI Scheme S7†). This approach yielded Dap9-Lat (6) in an excellent overall yield of 29% (over 30 steps). Notably, this solution phase cyclization strategy was found to scale well, providing convenient access to 6 in multi-gram quantities. Assessment of the antibacterial activities of laterocidine amide analogues 6 and 8 revealed them to largely mirror the activity of laterocidine itself, with the exception of the *A. baumannii* strains tested (Table 1). This is in marked contrast to the significant loss of activity observed for the corresponding brevicidine amide analogues 5 and 7, suggesting that the larger macrocycle present in laterocidine is more amenable to the ester-to-amide substitution. The serum stability of Dap9-Lat (6) was also found to be enhanced relative to laterocidine, with nearly 60% of the amide analogue still intact after 24 h incubation with serum, *versus* 45% for laterocidine itself (see ESI Fig. S7†).

**Fig. 4:**
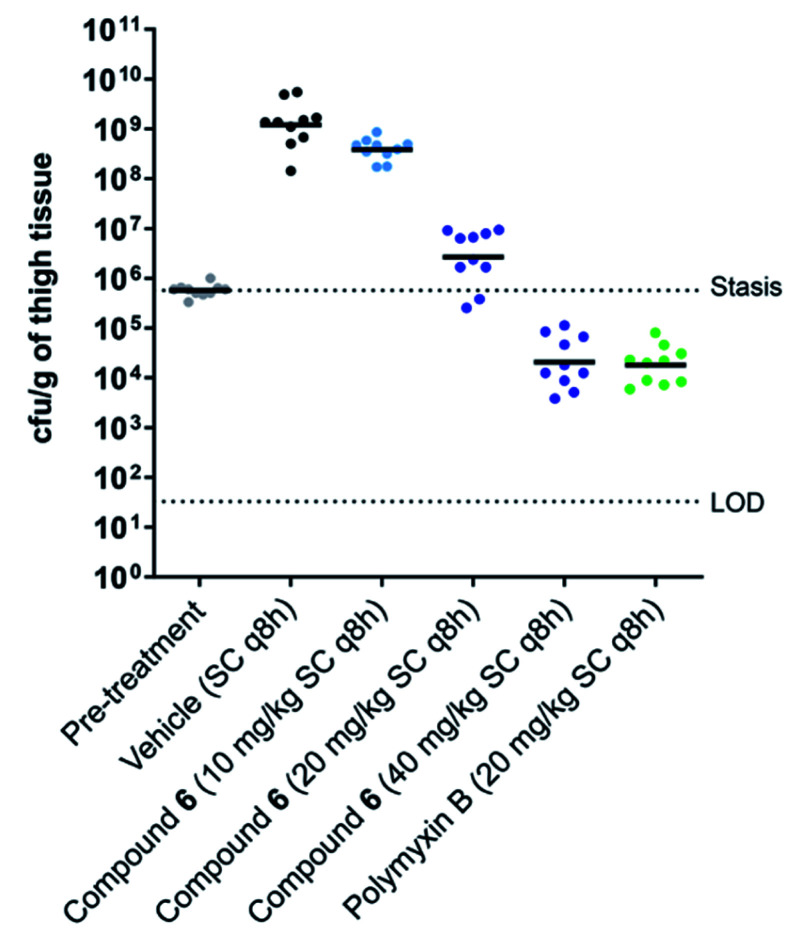
*In vivo* efficacy study. Scattergram of mouse thigh burdens (cfu g^−1^) following infection with *E. coli* ATCC 25922 and treatment with test articles, as indicated on the *x*-axis. The geometric mean burden of each treatment is indicated by the horizontal bar. LOD = limit of detection.

Building on these findings, brevicidine (1), laterocidine (2), and the synthetically more tractable amide analogues 5 and 6 were taken forward for further characterisation in cell toxicity assays. All compounds were found to be non-hemolytic up to the highest concentration tested (128 μg mL^−1^) (see ESI Fig. S8†). Brevicidine, laterocidine, and Dap9-Lat (6) were also found to be non-toxic to HepG2 cells at 128 μg mL^−1^, while Dap9-Brev (5) showed a slight indication of toxicity at the same concentration (see ESI Fig. S9†). Based on the favourable balance of antibacterial activity, stability, low cell toxicity, and synthetic accessibility, Dap9-Lat (6) was selected for further *in vivo* evaluation. To begin, the tolerability of the compound was assessed in naïve ICR mice, showing it to be well tolerated when dosed subcutaneously at 40 mg kg^−1^ every q8h over a 24 h period (total daily dose 120 mg kg^−1^). Building from this, an efficacy study was performed wherein Dap9-Lat (6) was further assessed for its capacity to reduce thigh infection in neutropenic mice infected with *E. coli* ATCC 25922. To gain an indication of dose-response, Dap9-Lat (6) was administered subcutaneously q8h at 10, 20, and 40 mg kg^−1^ and compared with groups treated with vehicle or polymyxin B as a clinical reference antibiotic administered subcutaneously q8h at 20 mg kg^−1^ (Fig. 4). A clear dose response was observed in the mice treated with Dap9-Lat (6), with the highest 40 mg kg^−1^ dose tested resulting in an approximate 5-log reduction in bacterial load relative to the untreated group, an antibacterial effect comparable to that observed for polymyxin B administered at 20 mg kg^−1^. Notably, these *in vivo* activities reflect the results of the *in vitro* activity assays, wherein Dap9-Lat (6) and polymyxin B were found to have MIC values against *E. coli* ATCC 25922 of 1.0 and 0.5 μg mL^−1^ respectively (Table 1). It is worthy of mention that in the original report describing the discovery and characterization of brevicidine and laterocidine,^6^ the quantities of material obtained from fermentation were not sufficient for comprehensive *in vivo* efficacy studies. In contrast, the convenient and scalable synthesis of laterocidine amide analogue 6 provides the opportunity for further optimization and evaluation of these promising lipopeptide antibiotics.

## Conclusions

In conclusion, we here report the total syntheses of the recently discovered lipodepsipeptide antibiotics brevicidine and laterocidine. In addition, a number of analogues of each were prepared including a particularly interesting variant of laterocidine wherein the ester linkage in the peptide macrocycle was substituted as an amide. This laterocidine analogue (6) maintains the potent anti-Gram-negative activity of the natural product *in vitro* and was also found to be efficacious *in vivo*. The *in vivo* efficacy of 6 is noteworthy given that in the original report describing the discovery of laterocidine, only modest *in vivo* activity was observed for the natural product,^6^ likely attributable to the low quantities of material available for dosing, coupled with its hydrolytic instability. In contrast, laterocidine amide analogue 6 exhibits enhanced stability, is readily synthesized on gram-scale, and exhibits a clear dose-dependent effect *in vivo*.

The routes here reported also provide reliable access to the natural products themselves. Brevicidine was obtained in 28 steps from 2-chlorotrityl resin in an overall yield of 9%, and laterocidine was obtained in 29 steps from Rink-amide resin in an overall yield of 2%. In both syntheses, formation of the macrocycle on-resin at an early stage was found to be most effective, serving to limit deleterious O → N acyl shifts at the ester linkage on Thr9, and provided the desired peptides as the major products after cleavage from resin, allowing for facile HPLC purification. Overall, both synthetic strategies are highly robust, yielding brevicidine and laterocidine in quantities that compare well with those obtained by isolation of the natural products from fermentation of the producing organisms. Synthetic brevicidine and laterocidine were shown to have identical ^1^H-NMR spectra and RP-HPLC elution profiles compared to their natural counterparts, confirming the previously reported structures. The antibacterial activities of synthetic brevicidine and laterocidine were also assessed against a panel of Gram-negative pathogens, demonstrating their potent antibacterial effect.

The methodology reported offers an efficient alternative to isolating these promising natural products from bacterial fermentation, and in doing so provides access to quantities of material suitable for further evaluation and mechanistic studies. Furthermore, the synthetic approaches we describe also provide access to novel analogues of both brevicidine and laterocidine. Of particular note is the finding that the enantiomeric forms of both brevicidine and laterocidine exhibit severely reduced antibacterial activities, a finding that supports a mechanism of action involving a stereospecific interaction with the bacterial target. Also of note is the finding that the macrocycles in brevicidine and laterocidine have varying tolerances for modification. Specifically, the ester-to-amide substitution, investigated as a means of both enhancing hydrolytic stability and increasing synthetic accessibility, was found to be detrimental to the activity of the brevicidines, while the laterocidine amide analogues largely maintain the activity of the natural product. Evaluation of the serum stability, haemolytic activity, and eukaryotic cell toxicity of the brevicidines and laterocidines here investigated in turn led the selection of laterocidine amide analogue 6 for further *in vivo* assessment. As noted above, these studies showed compound 6 to be well tolerated and capable of effectively reducing bacterial infection in a murine thigh-infection model. Also, while the clinically used polymyxins were found to be consistently more active (generally 2-to-4-fold lower MICs) than the brevicidines and laterocidines here studied, it is notable that in the case of mcr-positive polymyxin resistant strains, the brevicidines and laterocidines maintained their antibacterial activity. Given the “last-resort” status of the polymyxins, it is imperative that new antibacterial agents capable of overcoming polymyxin resistance be pursued.

In light of the increasing occurrence of Gram-negative pathogens with resistance to conventional antibacterial therapies, the brevicidine and laterocidine family of lipopeptide antibiotics represent promising leads for further development. In this regard, the recent discovery of the relacidines,^21^ which show high structural similarity to laterocidine, indicates that these lipopeptide antibiotics may be widespread in nature. The encouraging results we here report pave the way for future investigations aimed at further developing these promising lipopeptides with an eye to more fully characterizing their therapeutic potential. To this end, the synthetic approaches here described provide a convenient means to access structurally diverse analogues. In addition, mechanistic studies into how the brevicidines and laterocidines prevent the growth of polymyxin resistant strains are ongoing and will be reported in due course.

## Author contributions

KA, RDB, MH, and SJB synthesized peptides. KA, CMJW, ATB, NCB, and MvdS performed biological assays. ZZ and YXL performed LCMS analysis. SAC and NIM designed and lead the study. KA, RDB, SAC, and NIM prepared the manuscript.

## Conflicts of interest

A United Kingdom priority patent application has been jointly filed by Queen's University Belfast and Leiden University, covering the synthetic routes used for the preparation of brevicidine, laterocidine, as well as all novel analogues for use in the treatment of bacterial infection.

## Supplementary Material

SC-013-D2SC00143H-s001
