# Supplementary material for: Synthetic studies with the brevicidine and laterocidine lipopeptide antibiotics including analogues with enhanced properties and in vivo efficacy
Source: Chem Sci. 2022 Feb 23;13(12):3563–70. doi: 10.1039/d2sc00143h (PMC8943889; doi:10.1039/d2sc00143h)
Supplement: SC-013-D2SC00143H-s001 [file SC-013-D2SC00143H-s001.pdf]

Electronic Supplementary Material (ESI) for Chemical Science.
This journal is © The Royal Society of Chemistry 2022

**Supporting Information**

# Synthetic Studies with the Brevicidine and Laterocidine Lipopeptide Antibiotics Including Analogues with Enhanced Properties and *in vivo* Efficacy

Karol Al Ayed,[a,‡] Ross D. Ballantine,[b,‡] Michael Hoekstra,[a] Samantha J. Bann,[b] Charlotte M. J. Wesseling,[a] Alexander T. Bakker,[c] Zheng Zhong,[d] Yong-Xin Li,[d] Nora C. Brüchle,[a] Mario van der Stelt,[c] Stephen A. Cochrane,[b,]* Nathaniel I. Martin[a,]*

[a]*Biological Chemistry Group, Institute of Biology Leiden, Leiden University, Sylviusweg 72, 2333 BE Leiden, The Netherlands.*

[b]*School of Chemistry and Chemical Engineering, Queen's University Belfast, David Keir Building, Stranmillis Road, Belfast, BT9 5AG, UK.*

[c]*Molecular Physiology Group, Leiden Institute of Chemistry, Leiden University, Einsteinweg 55, 2333 CC Leiden, The Netherlands.*

[d]*Department of Chemistry, The University of Hong Kong, Pokfulam Road, Hong Kong, China*

[‡]These authors contributed equally.

**Corresponding authors email:* s.cochrane@qub.ac.uk, n.i.martin@biology.leidenuniv.nl

**Table of Contents**

| | |
|---|---|
| S2-10 | Supplemental Figures |
| S11-S17 | Supplemental Schemes |
| S18-S29 | Synthetic procedures |
| S30 | MIC determinations protocol |
| S31-32 | *In vivo* tolerability and efficacy studies |
| S33-S38 | Analytical RP-HPLC |
| S39-S58 | NMR characterization of compounds |
| S59 | References |

## *Supplemental Figures*

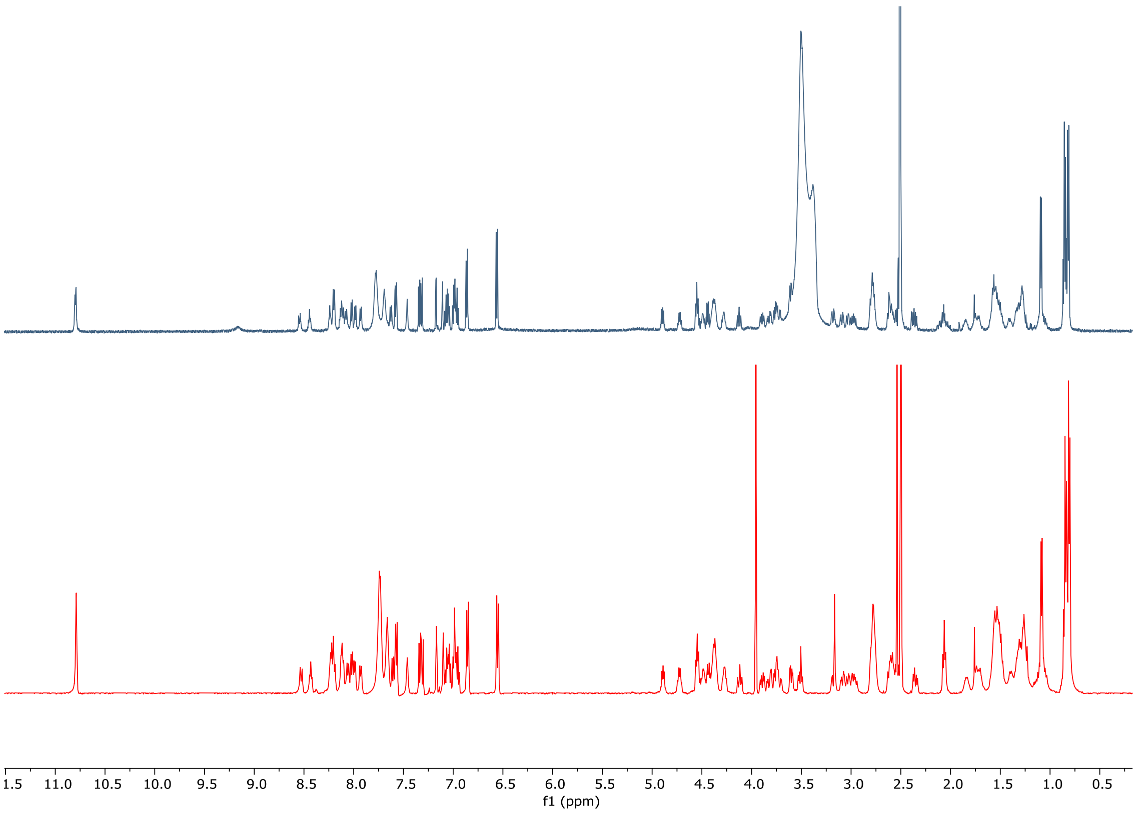


**Figure S1.** *[1]H NMR (600 MHz, DMSO-$d_6$) of synthetic brevicidine (top) overlaid with the previously published [1]H NMR (500 MHz, DMSO-$d_6$) spectrum of natural brevicidine (bottom) isolated from fermentation of the producing organism. Spectra were recorded at room temperature. The spectrum for the synthetic material contains broad signals between 3.30-3.65 ppm due to $H_20$/HDO present in the NMR solvent. The peak at ca. 3.96 ppm in the published spectrum of brevicidine is attributed to an impurity not present in the synthetic material.*

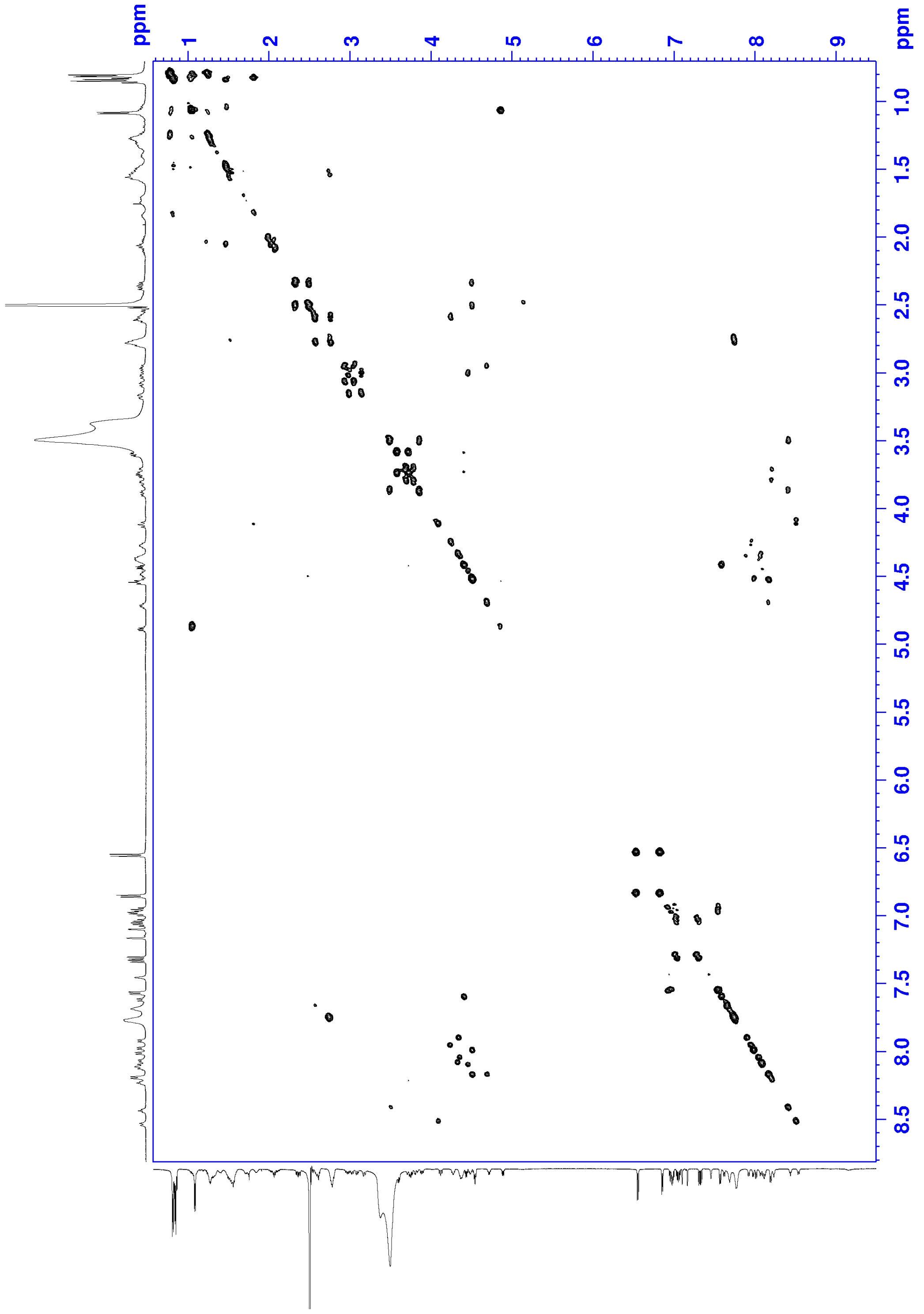

**Figure S2.** *2D COSY NMR (600 MHz, DMSO-$d_6$) of synthetic brevicidine*

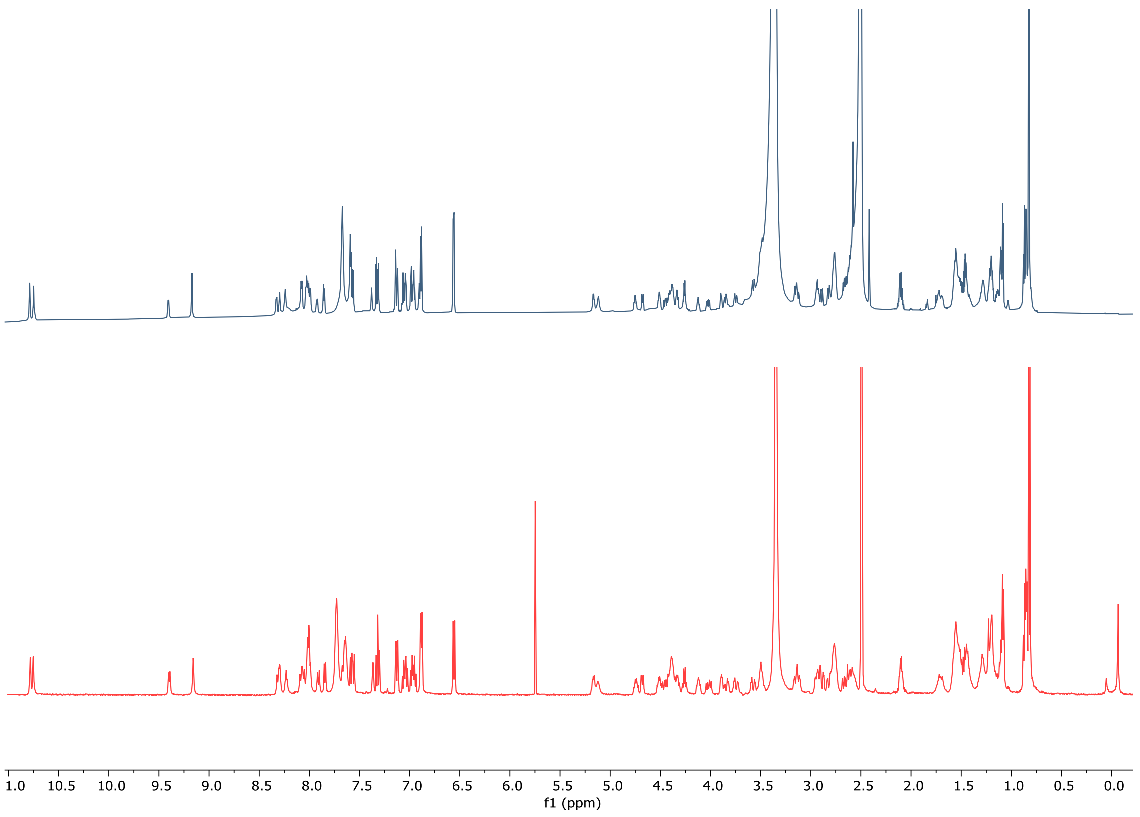


**Figure S3.** *[1]H NMR (850 MHz, DMSO-$d_6$) of synthetic laterocidine (top) overlaid with the previously published [1]H NMR (500 MHz, DMSO-$d_6$) spectrum of natural laterocidine (bottom) isolated from fermentation of the producing organism. Spectra were recorded at room temperature.*

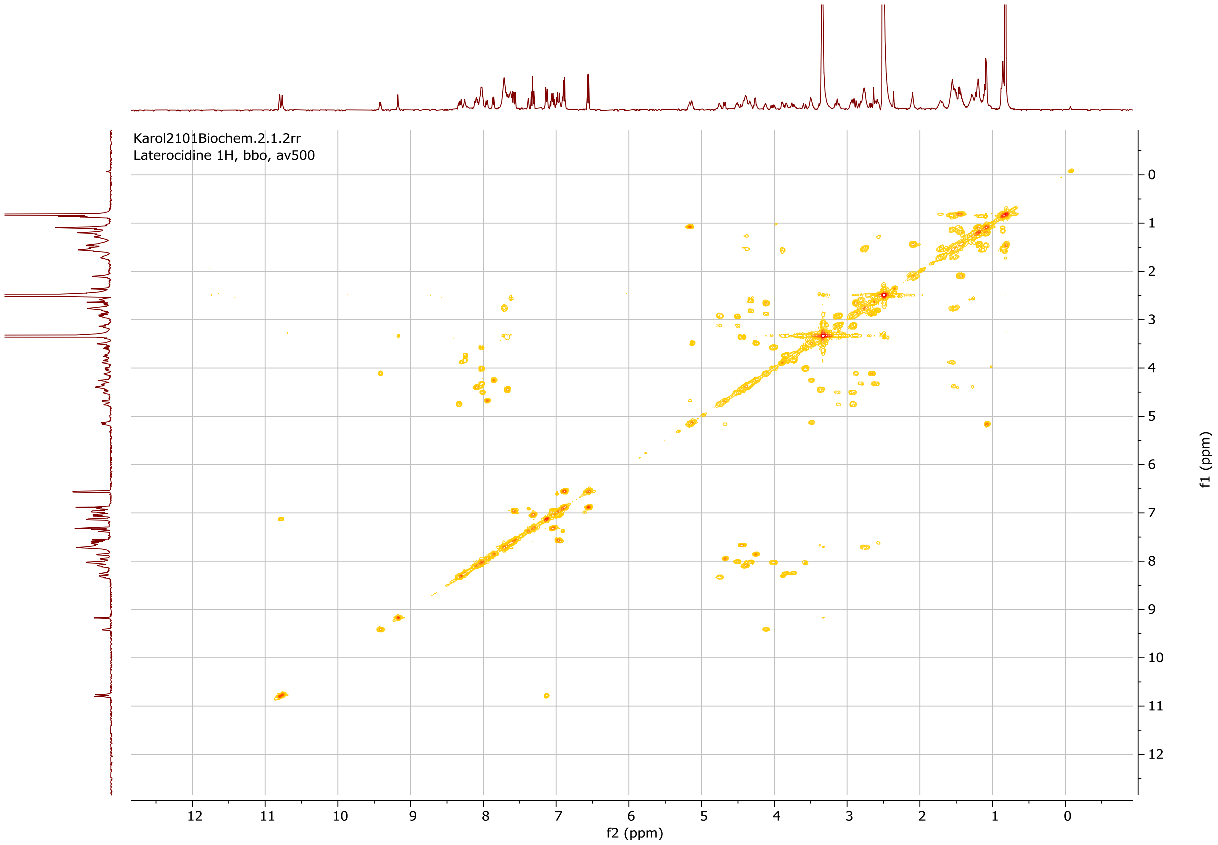


**Figure S4.** *2D COSY NMR (500 MHz, DMSO-d$_6$) of synthetic laterocidine*

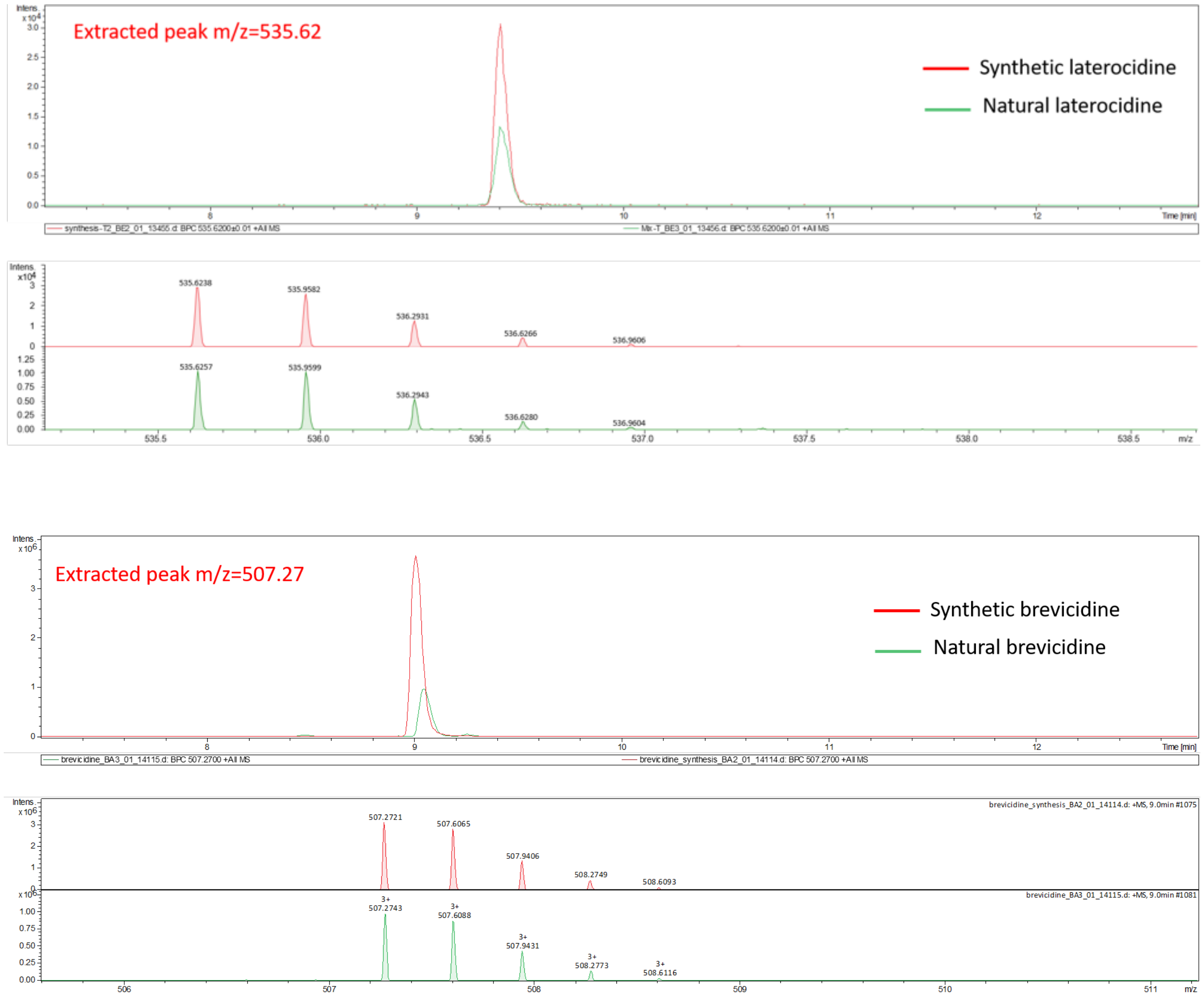


**Figure S5.** Comparison of synthetic lipopeptides with their natural counterparts by LC-MS/MS. **LC-MS/MS acquisition:** UltiMate 3000 UHPLC Systems coupled to Bruker impact™II Mass Spectrometer (TOF). **Column:** Waters Acquity UPLC BEH C18 column (1.7 µm, 130 Å, 2.1 × 150 mm). **LC method:** The column was maintained at 40˚C and run at a flow rate of 0.2 mL/min, using 0.1% formic acid in $H_2O$ as solvent A and 0.1% formic acid in acetonitrile as solvent B. A gradient was employed for chromatographic separation starting at 5% B for 2 min, then 5% to 95% B for 15 min, and finally held at 95% for 4 min, The column was re-equilibrated to 5% B for 1 min before the next run was started. **MS method:** The MS system was tuned using standard sodium formate solution. The same solution was used to calibrate the system before starting. All the samples were analyzed in positive polarity, using data-dependent acquisition mode. Detection range: 100-1500 m/z.

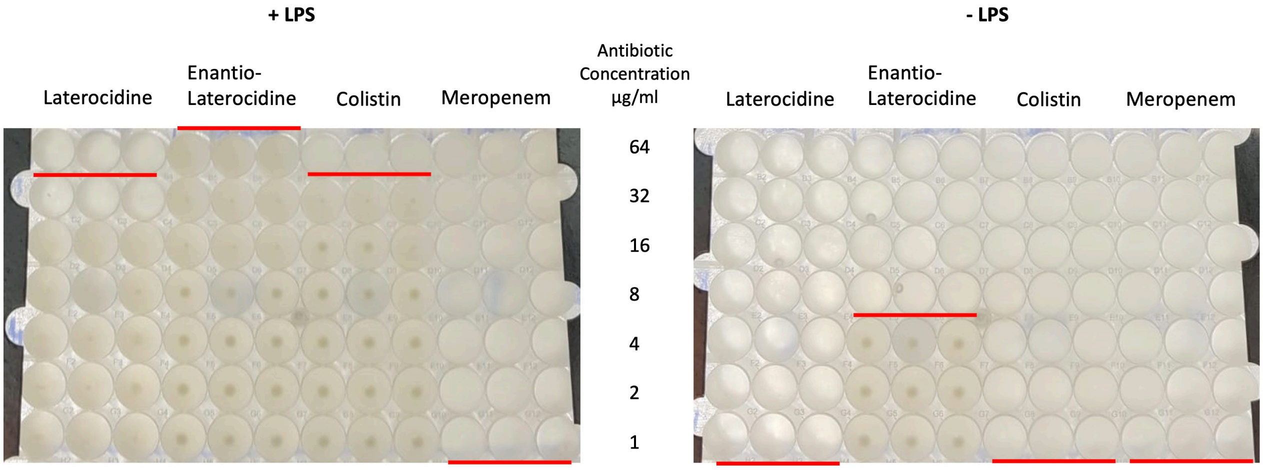


**Figure S6.** LPS antagonization assay. A blood agar plate was inoculated with a glycerol stock of *E. coli* ATCC 25922. The inoculated agar plate was then incubated for 16 h at 37 °C. An individually grown colony was subsequently used to inoculate 3 mL of TSB that was then incubated at 37 °C with shaking at 220 rpm. In parallel, the compounds to be assessed were serially diluted with Mueller-Hinton broth (MHB) in polypropylene 96-well plates (50 µL in each well). Once the OD600 of the bacterial suspensions reached 0.5, the bacteria were diluted with MHB (final concentration $2 \times 10^5$ CFU $mL^{-1}$). The media were then either supplemented with 1 mg/mL of LPS (lipopolysaccharides from *E. coli* O55:B5, Sigma-Aldrich) or added directly to the microplates containing the test compounds (50 µL to each well, final volume: 100 µL). The well-plates were sealed with an adhesive membrane and after 16 h of incubation at 37°C with shaking at 220 rpm. The wells were visually inspected for bacterial growth.

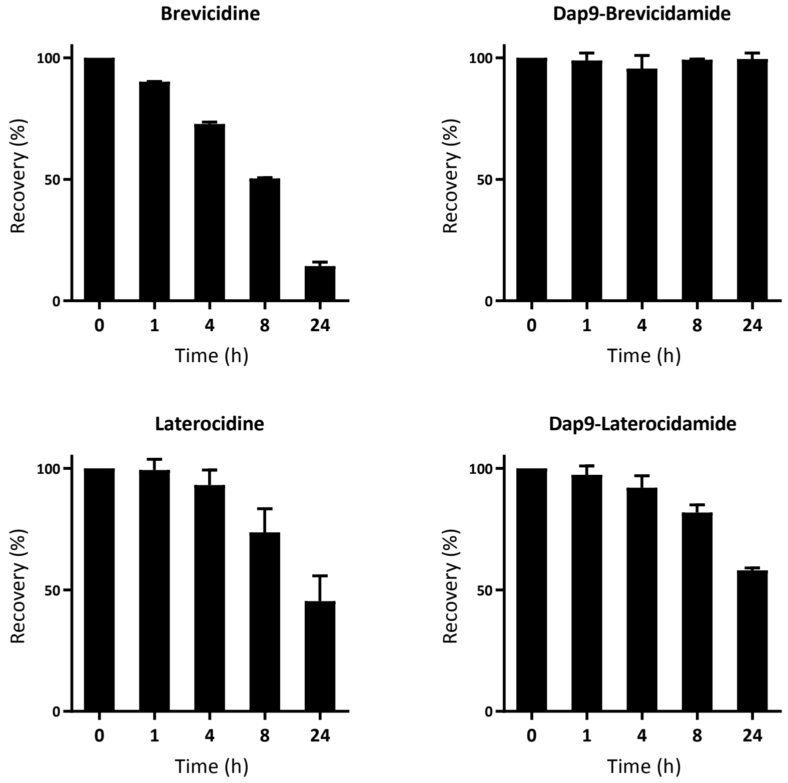


**Figure S7.** Serum stability assays comparing natural lipopeptides to synthetic amide analogues. 10 mg/mL peptide solutions were prepared in in Milli-Q water. Samples were prepared with 42 µL peptide solution and 518 µL human serum (obtained from Sigma Aldrich, product number: H4522) and incubated at 37 °C. Samples were taken at t = 0, 1, 4, 8 and 24 h. To 100 µL of serum, 100 µL of 6% TCA in ACN (containing 0.2 µg/mL D-Phenylalanine as internal standard) was added to precipitate the proteins. The samples were vortexed, left for 15 min at room temperature and stored at -20 °C. Before analysis the samples were centrifuged for 5 min at 13 000 rpm. The supernatant was analyzed by RP-HPLC using a Shimadzu Prominence-i LC-2030 system with a Dr. Maisch ReproSil Gold 120 C18 column (4.6 × 250 mm, 5 µm) at 30 °C and equipped with a UV detector monitoring at 220 nm and 254 nm. The following solvent system, at a flow rate of 1 mL/min, was used: solvent A, 0.1 % TFA in water/acetonitrile 95/5; solvent B, 0.1 % TFA in water/acetonitrile 5/95. Gradient elution was as follows: 100:0 (A/B) for 2 min, 100:0 to 50:50 (A/B) over 45 min, 50:50 (A/B) to 0:100 (A/B) over 1 min, 0:100 (A/B) for 6 min then reversion back to 100:0 (A/B) over 1 min, 100:0 (A/B) for 5 min. The peaks were integrated and normalized to the internal standard. Recovery of the peptides at t=0 was compared to control samples without serum and was within the 85%-115% range (data not shown). The t=0 value was then set at 100% for each analogue and all time-points were calculated as a percentage of t=0. Biological duplicates of the experiment were performed.

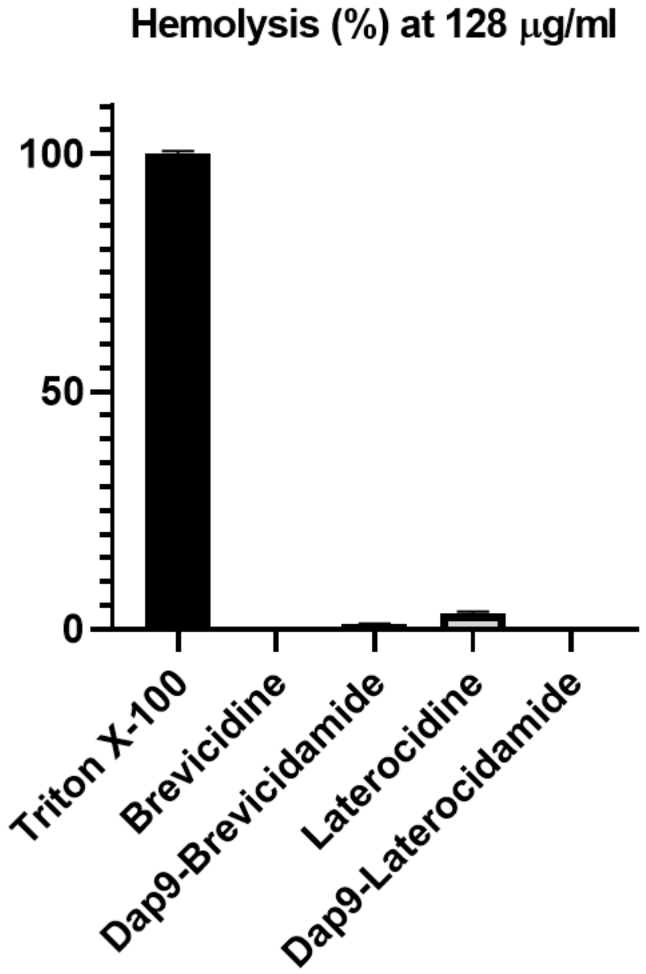


**Figure S8.** Hemolytic activity of selected analogues against sheep red blood cells. Experiments were performed in triplicate and Triton X-100 used as a positive control. Red blood cells from defibrinated sheep blood obtained from Thermo Fisher were centrifuged (400 g for 15 min at 4°C) and washed with Phosphate-Buffered Saline (PBS) containing 0.002% Tween20 (buffer) for five times. Then, the red blood cells were normalized to obtain a positive control read-out between 2.5 and 3.0 at 415 nm to stay within the linear range with the maximum sensitivity. A serial dilution of the compounds (200 – 6.25 µg/mL, 75 µL) was prepared in a 96-well plate. The outer border of the plate was filled with 75 µL buffer. Each plate contained a positive control (0.1% Triton-X final concentration, 75 µL) and a negative control (buffer, 75 µL) in triplicate. The normalized blood cells (75 µL) were added and the plates were incubated at 37 °C for 20 h while shaking at 500 rpm. A flat-bottom plate of polystyrene with 100 µL buffer in each well was prepared. After incubation, the plates were centrifuged (800 g for 5 min at room temperature) and 25 µL of the supernatant was transferred to their respective wells in the flat-bottom plate. The values obtained from a read-out at 415 nm were corrected for background (negative control) and transformed to a percentage relative to the positive control.

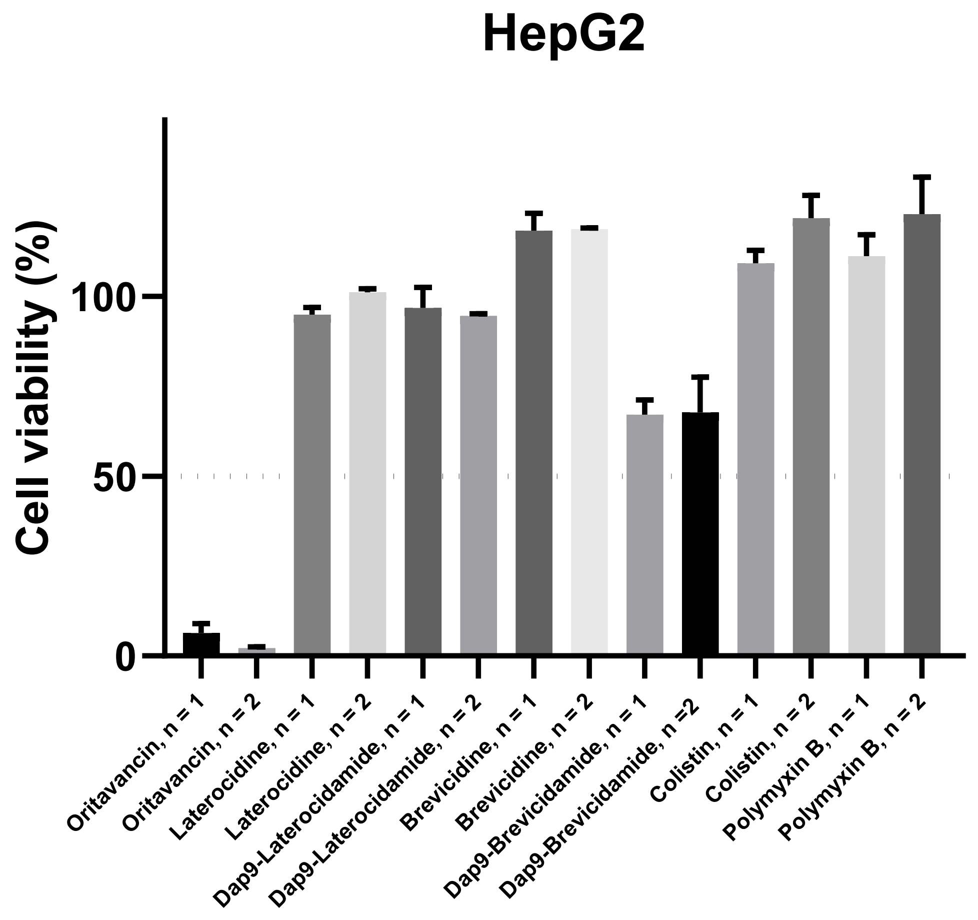


**Figure S9.** Cytotoxicity of selected brevicidine and laterocidine analogues against HepG2 cells compared to oritavancin, colistin and polymyxin B using a (3-(4,5-dimethylthiazol-2-yl)-2,5-diphenyltetrazolium bromide (MTT) assay. HepG2 cells were seeded at a density of $1.5 \times 10^4$ cells per well in a clear 96-well tissue culture treated plate in a final volume of 100 µL of Dulbecco's Modified Eagle Medium (DMEM), supplemented with Fetal Bovine Serum (1%), Glutamax and Pen/Strep. Cells were incubated for 24 h at 37°C, 7% $CO_2$ to allow cells to attach to the plates. In addition to a single vehicle control, compounds (diluted from DMSO stock) were added to each well to obtain in a final 128 µg/mL concentration (max. final DMSO concentration 0.5%) for all compounds except for oritavancin which was administered at 50 µM. Incubation was done for 24 h at 37 °C, 7% $CO_2$. After the incubation, MTT was added to each well at a final concentration of 0.40 mg/mL. The plates were then incubated for 2 h at 37 °C, 7% $CO_2$. Medium was carefully removed via suction, and purple formazan crystals were resuspended in 100 µL DMSO. Absorbance was read at 570 nm using a Clariostar plate reader. The data was then analysed with GraphPad Prism software. Technical triplicates for each condition were used, along with biological duplicates.

## *Supplemental Schemes*

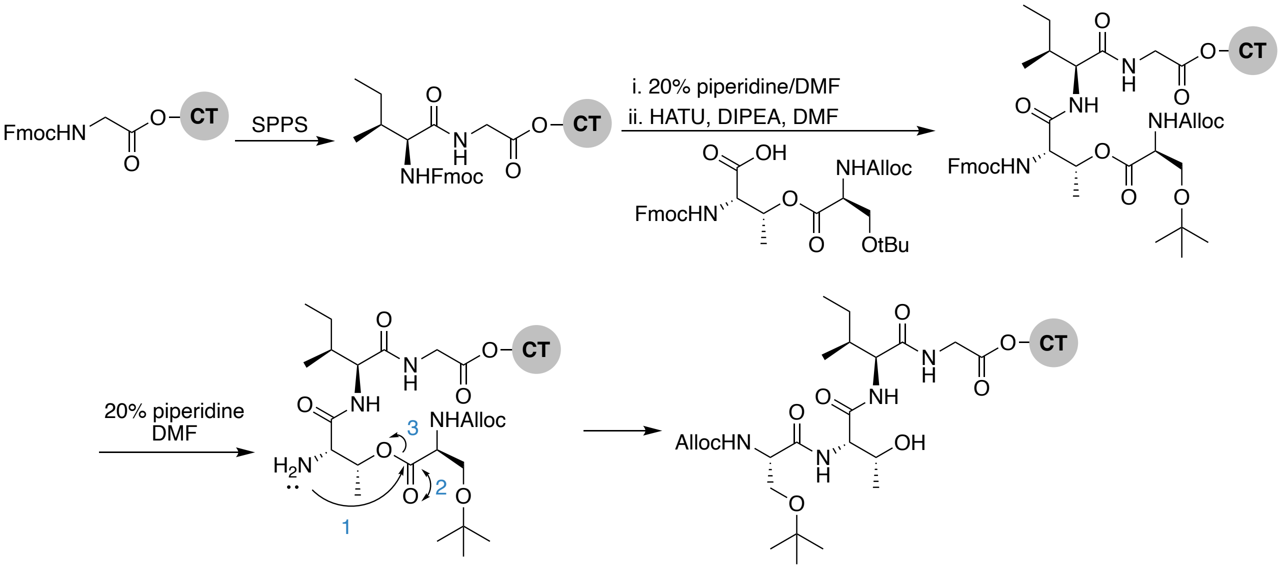


**Scheme S1.** Attempted synthesis of brevicidine failed due to deleterious *O*→*N* acyl shift. CT = 2-chlorotrityl resin.

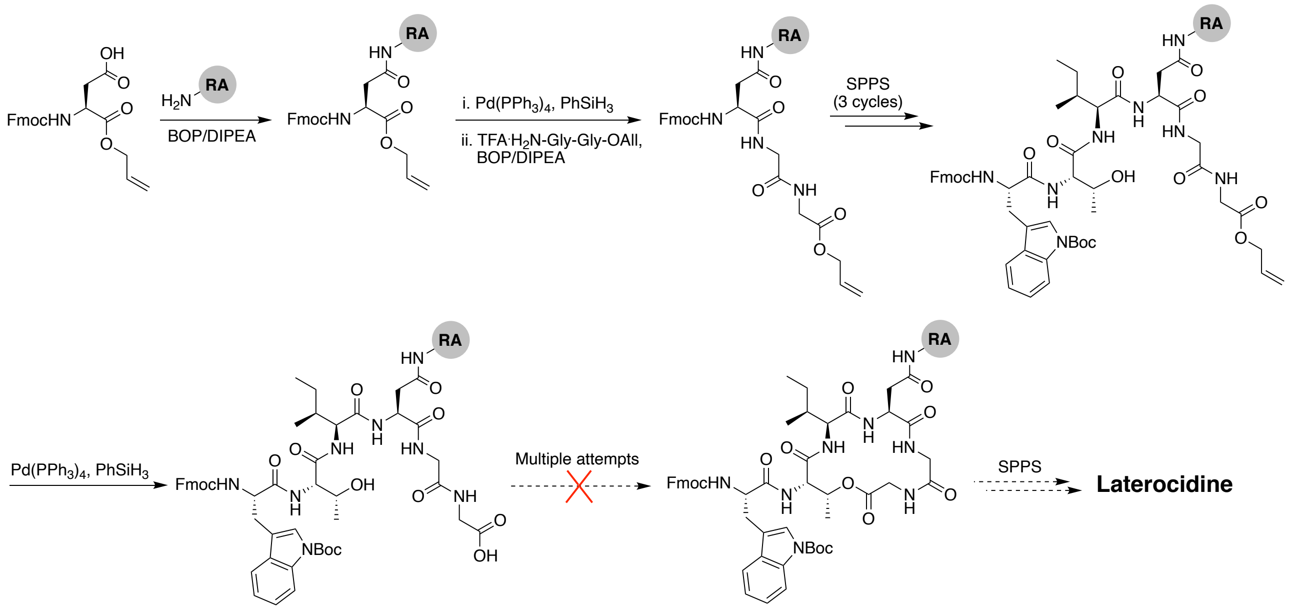


**Scheme S2.** Attempted synthesis of laterocidine failed at macrolactonization stage. RA = Rink amide resin.

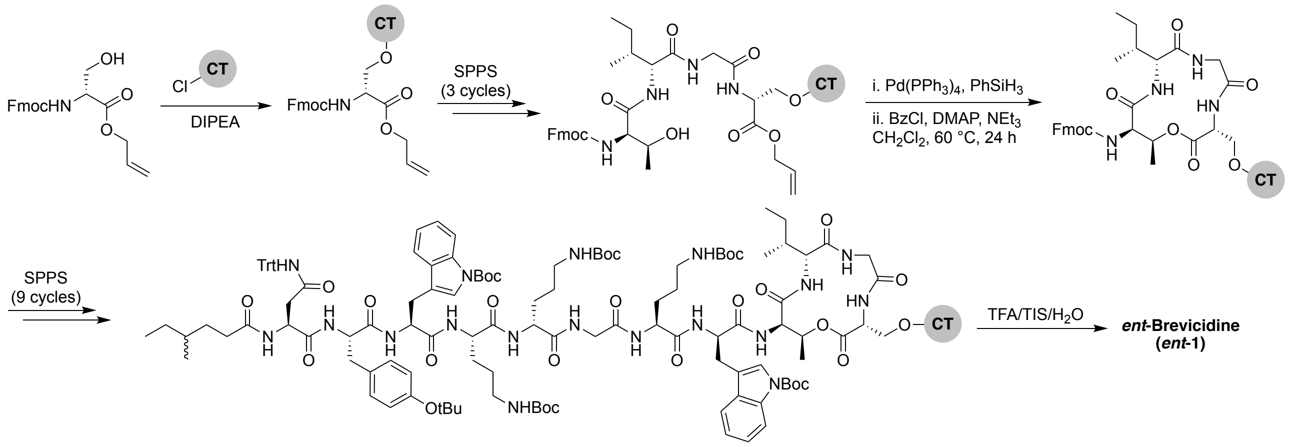


**Scheme S3.** Total SPPS of *ent*-brevicidine (***ent-1***). CT = 2-chlorotrityl resin.

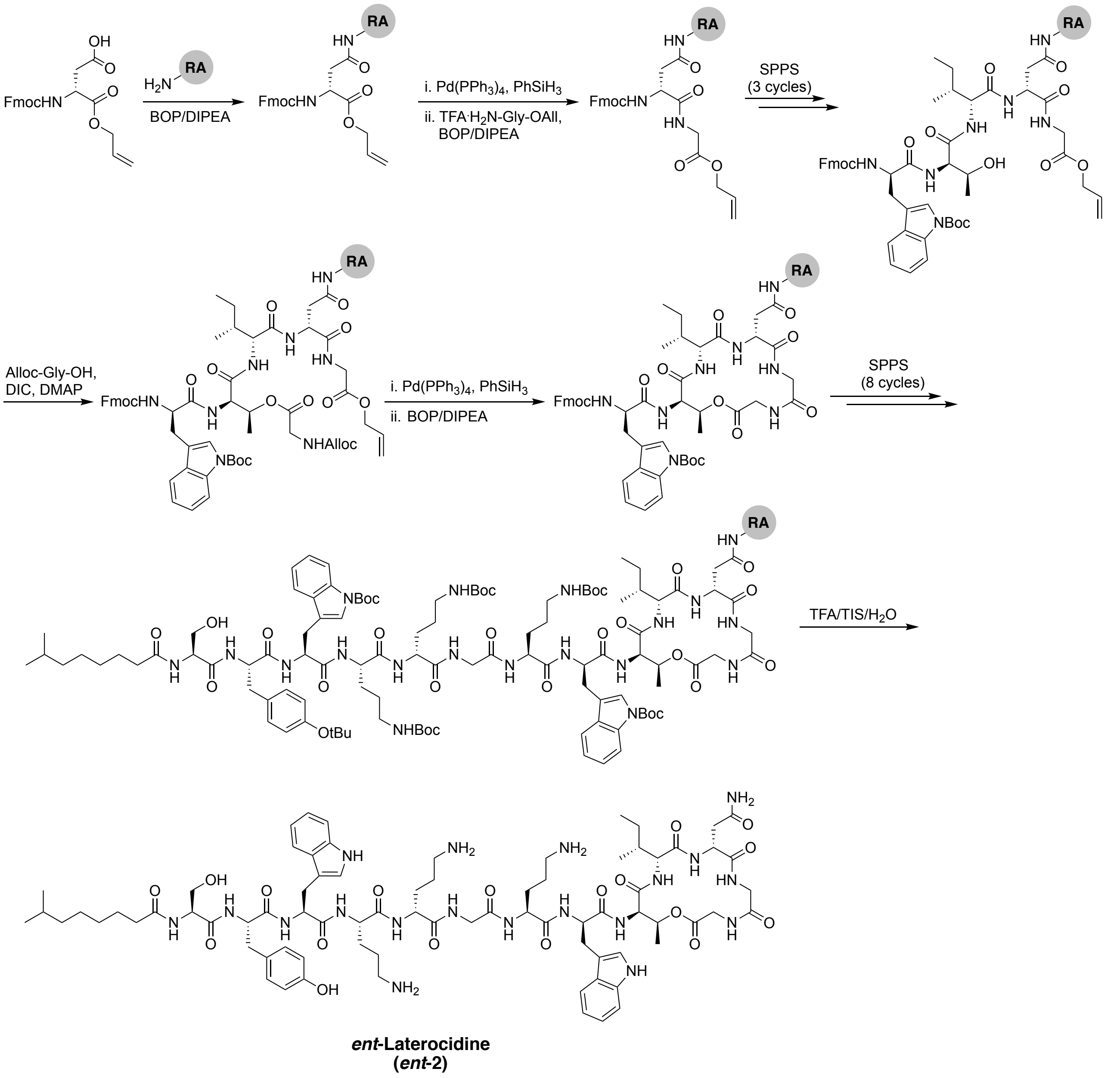


**Scheme S4.** Total SPPS of *ent*-laterocidine (***ent-2***). RA = Rink amide resin.

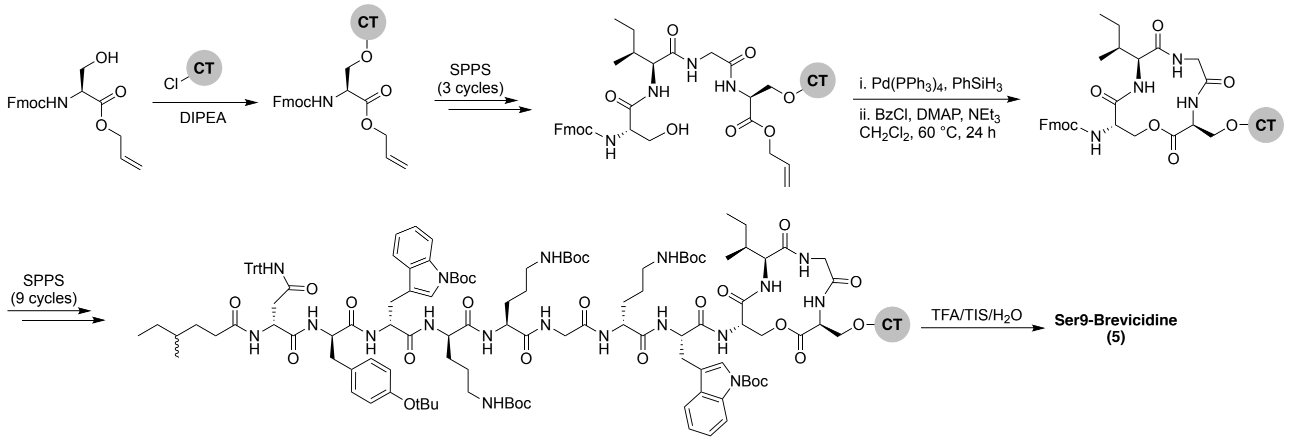


**Scheme S5.** Total SPPS of Ser9-brevicidine (**3**). CT = 2-chlorotrityl resin.

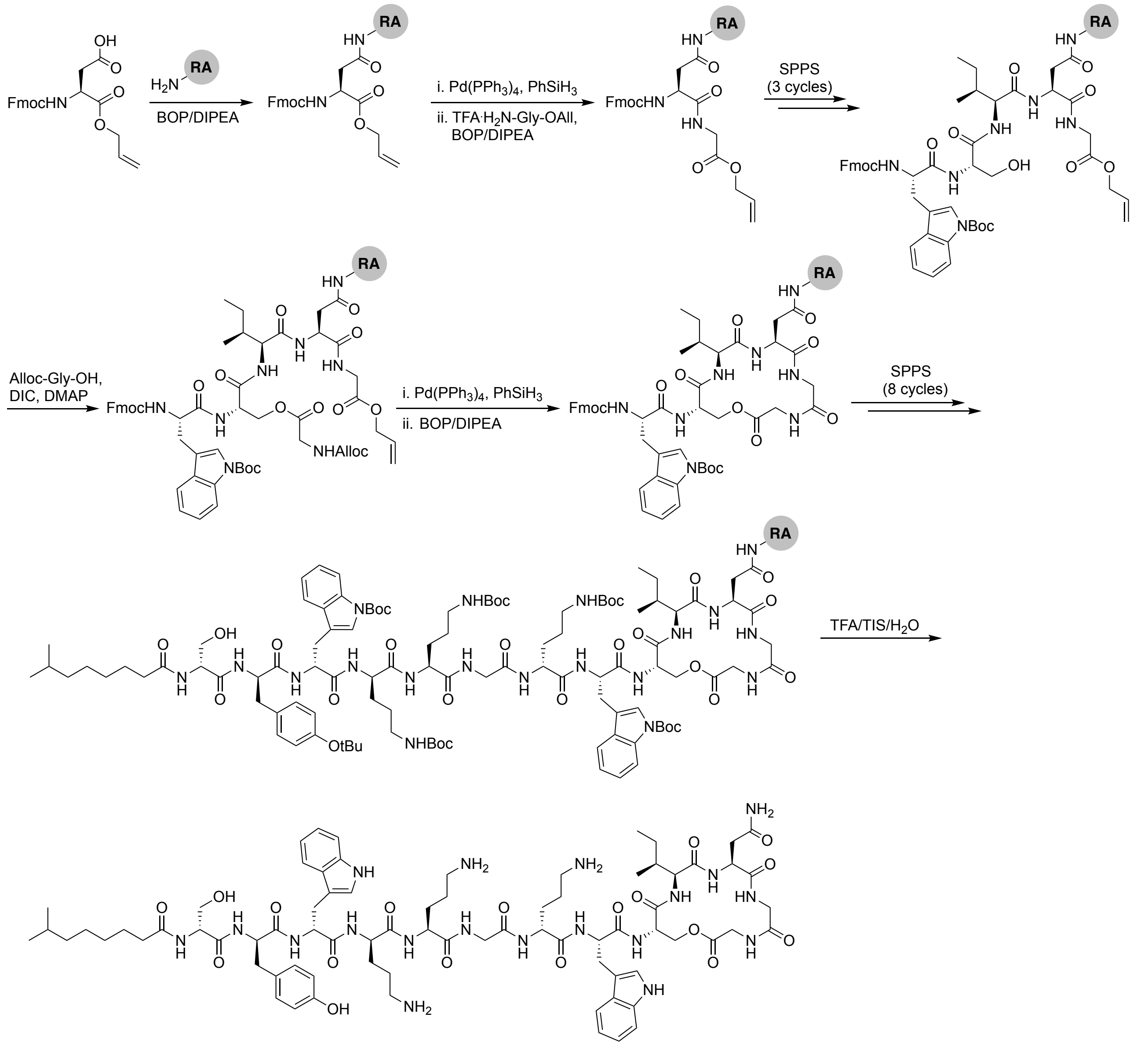


**Ser9-Laterocidine (4)**

**Scheme S6.** Total SPPS of Ser9-laterocidine (**4**). RA = Rink amide resin.

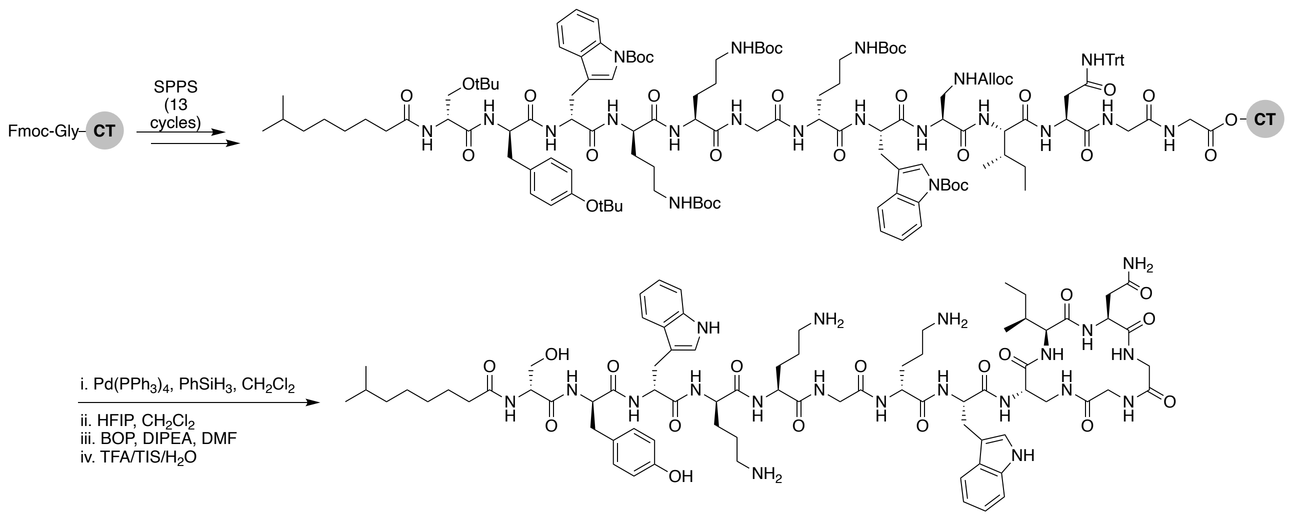


**Scheme S7**. Combination synthesis of Dap9-laterocidine (**6**) where the entire protected linear peptide is assembled by SPPS, and cyclized and deprotected in solution. CT = 2-chlorotrityl resin.

***Synthetic Procedures***

***Reagents and general methods***

All reagents employed were of American Chemical Society (ACS) grade or higher and were used without further purification unless otherwise stated. Fmoc-Ser-OAll,[1] Fmoc-Asp-OAll,[2] TFA·$NH_2$-Gly-OAll,[3] TFA·$NH_2$-Gly-Gly-OAll[4] and (2S,3R)-2-((((9H-fluoren-9-yl)methoxy)carbonyl)amino)-3-azidobutanoic acid[5] were synthesized according to referenced literature procedures. The NMR characterization of brevicidine in DMSO-$d_6$ was obtained using a Bruker Ascend 600 (600 MHz). The NMR characterization of laterocidine in DMSO-$d_6$ was obtained on a Bruker AV850 spectrometer (850 MHz). LC-MS analyses were performed on a Shimadzu LC-20AD system with a Shimadzu Shim-Pack GISS-HP C18 column (3.0 x 150 mm, 3 μm) at 30°C and equipped with a UV detector. The following solvent system, at a flow rate of 0.5 mL/min, was used: solvent A, 0.1 % formic acid in water; solvent B, acetonitrile. Gradient elution was as follows: 95:5 (A/B) for 2 min, 95:5 to 0:100 (A/B) over 13 min, 0:100 (A/B) for 2 min, then reversion back to 95:5 (A/B) over 1 min, 95:5 (A/B) for 2 min. This system was connected to a Shimadzu 8040 triple quadrupole mass spectrometer (ESI ionisation). The NMR characterisation of brevicidine and derivatives in DMSO-$d_6$ was obtained using a Bruker Ascend 600 (600 MHz). LC-MS analyses were conducted on an Agilent 1260 HPLC (equipped with an Infinity II quaternary pump, vial sampler, integrated column compartment and a variable wavelength detector) and MSD single quadropole mass spectrometer. Samples were analysed using an Agilent Infinitylab poroshell 120 column (2.1 x 150 mm, 2.7 μm) under acetonitrile/water gradient with 0.1% formic acid as an additive. The following solvent system, at a flow rate of 0.3 mL/min, was used: solvent a, 0.1 % formic acid in water, solvent B, 0.1 % formic acid in acetonitrile. Gradient elution was as follows: 95:5 to 0:100 (A/B) over 5 min, 0:100 (A/B) for 1 min, then reversion back to 95:5 (A/B) over 0.1 min.

***HPLC purification of synthetic peptides***

Brevicidine and analogues were purified using a Perkin Elmer HPLC system composed of a 200 series binary pump, UV/Vis detector monitoring at 220 nm, vacuum degasser and Rheodyne 7725i injector. **Method A (Preparative):** Phenomenex Luna C18 column (21.2 x 250 mm, 5 μm) with a 2 mL injection loop. The following solvent system, at a flow rate of 10 mL/min, was used: solvent A, 0.1 % TFA in water; solvent B, acetonitrile. Gradient elution was as follows: 80:20 (A/B) for 5 min, 80:20 to 45:55 (A/B) over 30 min, 45:55 to 0:95 (A/B) over 3 min, 0:95 (A/B) for 3 min then reversion back to 80:20 (A/B) over 2 min, 80:20 (A/B) for 5 min. **Method B (Analytical):** Phenomenex C18 Luna column (4.6 x 150 mm, 5 μm) with a 200 μL injection loop. The following solvent system, at a flow rate of 2 mL/min, was used: solvent A, 0.1 % TFA in water; solvent B,

acetonitrile. Gradient elution was as follows: 95:5 (A/B) for 2 min, 95:5 to 5:95 (A/B) over 18 min then reversion back to 95:5 (A/B) over 0.1 min, 95:5 (A/B) for 3.9 min. Laterocidine and analogues were purified using the following methods. **Method C (Preparative):** BESTA-Technik system equipped with a ECOM Flash UV detector monitoring at 214 nm and 254 nm with a Dr. Maisch Reprosil Gold 120 C18 column (25 × 250 mm, 10 µm). The following solvent system, at a flow rate of 12 mL/min, was used: solvent A, 0.1 % TFA in water/acetonitrile 95/5; solvent B, 0.1 % TFA in water/acetonitrile 5/95. Gradient elution was as follows: 100:0 (A/B) for 5 min, 100:0 to 50:50 (A/B) over 50 min, 50:50 to 0:100 (A/B) for 3min, then reversion back to 100:0 (A/B) over 1 min, 100:0 (A/B) for 5 min. **Method D (Analytical):** Shimadzu Prominence-i LC-2030 system with a Dr. Maisch ReproSil Gold 120 C18 column (4.6 × 250 mm, 5 µm) at 30 °C and equipped with a UV detector monitoring 214 nm and 254 nm. The following solvent system, at a flow rate of 1 mL/min, was used: solvent A, 0.1 % TFA in water/acetonitrile 95/5; solvent B, 0.1 % TFA in water/acetonitrile 5/95. Gradient elution was as follows: 100:0 (A/B) for 2 min, 100:0 to 50:50 (A/B) over 45 min, 50:50 (A/B) to 0:100 (A/B) over 1 min, 0:100 (A/B) for 6 min then reversion back to 100:0 (A/B) over 1min, 100:0 (A/B) for 5min.

***Synthesis of Brevicidine (1)***

To a flame dried 25 mL round bottom flask was added Fmoc-Ser-OAll (110 mg, 0.300 mmol) and dry dichloromethane (DCM) (10.0 mL). 2-Chlorotrityl chloride resin (CT) (1.00 g, 0.81 mmol $g^{-1}$) and DIPEA (210 µL, 1.20 mmol) were added. The suspension was stirred under argon for 48 h, after which the resin was filtered through a manual SPPS vessel and washed with DCM (4 x 5 mL). The resin was then capped by adding a solution of methanol, DIPEA and DCM (3 mL, 10 : 5 : 85) and bubbled with argon for 1 h. The solution was discharged and the resin was washed with DCM (3 x 5 mL) before being dried under a stream of argon. A small portion of resin was then used to ascertain the loading. Estimation of loading level of first residue onto resin (0.15 mmol $g^{-1}$) was calculated via an Fmoc loading test, as described by Gude *et al.*[6]

Standard Fmoc SPPS protocol was used to extend the peptide to the linear Fmoc-Thr-Ile-Gly-Ser stage. Specifically, resin (670 mg, 0.1 mmol) was added to a manual SPPS vessel and bubbled in DMF (3 mL) to swell. The solvent was discharged and the resin was bubbled in an Fmoc deprotection solution of 20% piperidine in DMF (3 x 3 mL, 2 x 1 min then 1 x 5 min) with argon. The resin was washed with DMF (3 x 3 mL) and a coupling solution of amino acid (6 equiv), HATU (6 equiv) and DIPEA (12 equiv) in DMF (3 mL) was added. The solution was then bubbled with argon for 1 h, before the solution was discharged and the resin washed with DMF (3 x 3 mL). This process was repeated to obtain on-resin linear Fmoc-tetrapeptide. At this stage the resin was

split an a portion of this on-resin allyl-protected tetrapeptide (78.0 mg. 0.01 mmol) was added to a manual SPPS vessel and bubbled in DCM (3 mL) with argon for 15 min. The solvent was discharged and an allyl deprotection solution of tetrakis(triphenylphosphine) palladium (231 mg, 0.200 mmol) and phenylsilane (123 µL, 0.998 mmol) in DCM and DMF (1:1, 2 mL) was added. The solution was bubbled with argon for 2 h in darkness, after which the deprotection solution was discharged and the resin was washed with DMF (3 x 3 mL), 0.5 % sodium diethyldithiocarbamate solution in DMF (4 x 5 mL), DMF (4 x 5 mL) and DCM (4 x 5 mL). The resin was dried under argon, then added to a 5 mL flame dried round bottom flask under argon. Dry dichloromethane (3 mL) was added and the suspension stirred for 15 min. Benzoyl chloride (13.0 µL, 0.112 mmol), triethylamine (3.00 µL, 22.0 µmol) and catalytic DMAP (1 crystal) were added and the reaction mixture was stirred overnight at 60 °C. The resin was then filtered through a manual SPPS vessel and washed with DMF (3 x 5 mL) and DCM (3 x 5 mL) before being dried under argon. To ascertain reaction progress, a small sample was cleaved using a 2 % TFA solution in DCM (1 mL). The cleavage cocktail was gently agitated for 1 h and filtered through a glass wool plug. The filtrate was concentrated with a gentle stream of argon before being analysed by LC-MS. Desired cyclic product was identified ($[M+H]^+$ calculated for $C_{30}H_{36}N_4O_8$ 581.2, found (LC-MS) 581.5) Following this modified Yamaguchi esterification, the synthesis of brevicidine was completed using standard Fmoc SPPS protocols as described above, after which 4-methylhexanoic acid was coupled to the N-terminus. The dried resin was then added to a cleavage cocktail of TFA, TIPS and distilled water (10 mL, 95 : 2.5 : 2.5) and heated to 37°C for 1 h. The suspension was filtered through a glass wool plug and the filtrate concentrated under vacuum. Diethyl ether was used to precipitate the crude peptide, which was then centrifuged and washed with additional diethyl ether. The suspension was centrifuged and the pellet dissolved in a minimal amount of 1:1 acetonitrile and water solution with 0.1% TFA. The crude mixture was subsequently purified by RP-HPLC (See HPLC purification of synthetic peptides).Fractions were assessed by LC-MS and product-containing fractions were pooled, frozen and lyophilized to yield brevicidine as a white powder. Yield: 13 mg, 9% over 28 steps. HPLC retention time 23.3 min (Method A); $[M+3H]^{3+}$ calculated for $C_{74}H_{106}N_{18}O_{17}$ 507.2734, found (HR-MS) 507.2688.

***Synthesis of ent-Brevicidine (ent-1)***

To a flame dried 25 mL round bottom flask was added Fmoc-D-Ser-OAll (112 mg, 0.305 mmol) and dry dichloromethane (10.0 mL). 2-Chlorotrityl chloride resin (CT) (1.00 g, 0.81 mmol $g^{-1}$) and DIPEA (210 µL, 1.20 mmol) were added. The suspension was stirred under argon for 48 h at 45 °C, after which the resin was filtered through a manual SPPS vessel and washed with DCM (4 x 5 mL).

The resin was then capped and loading level of the first residue onto resin (0.25 mmol $g^{-1}$) was calculated as per the synthesis of brevicidine.

Standard Fmoc SPPS protocol was used to extend the peptide to the linear Fmoc-D-Thr-D-Ile-Gly-D-Ser peptide on a 0.1 mmol scale (400 mg) similar to the synthesis of brevicidine. Following SPPS of the tetrapeptide, an allyl deprotection solution of tetrakis (triphenylphosphine) palladium (231 mg, 0.200 mmol) and phenylsilane (123 µL, 0.998 mmol) in DCM and DMF (1:1, 2 mL) was added. The solution was bubbled with argon for 2 h in darkness, after which the deprotection solution was discharged and the resin was washed with DMF (3 x 3 mL), 0.5 % sodium diethyldithiocarbamate solution in DMF (4 x 5 mL), DMF (4 x 5 mL) and DCM (4 x 5 mL). The resin was dried under argon, then added to a 5 mL flame dried round bottom flask under argon. Dry dichloromethane (3 mL) was added and the suspension stirred for 15 min. Benzoyl chloride (13.0 µL, 0.112 mmol), triethylamine (3.00 µL, 22.0 µmol) and catalytic DMAP (1 crystal) were added and the reaction mixture was stirred overnight at 60°C. The resin was then filtered through a manual SPPS vessel and washed with DMF (3 x 5 mL) and DCM (3 x 5 mL) before being dried under argon. A small sample was cleaved using a 2 % TFA solution in DCM (1 mL). The cleavage cocktail was gently agitated for 1 h and filtered through a glass wool plug. The filtrate was concentrated with a gentle stream of argon before being analysed by LC-MS ($[M+Na]^+$ calculated for $C_{30}H_{36}N_4O_8$ 603.2, found (LC-MS) 603.5). Following this modified Yamaguchi esterification, the synthesis of *ent*-brevicidine was completed using standard Fmoc SPPS protocols as described above after which 4-methylhexanoic acid was coupled to the N-terminus. The peptide was cleaved from resin, precipitated and purified by RP-HPLC (See HPLC purification of synthetic peptides) following the procedure for synthesising brevicidine. Yield: 3 mg, 2% over 28 steps. HPLC retention time 22.9 min; $[M-H]^-$ calculated for $C_{74}H_{106}N_{18}O_{17}$ 1517.7910, found (HR-MS) 1517.7943.

***Synthesis of Ser9-Brevicidine (3)***

The desired Fmoc-tetrapeptide was synthesized from Fmoc-Ser-OAll as described above, with Fmoc-Ser used in place of Fmoc-Thr. This resin-bound tetrapeptide (0.065 mmol, 0.14 mmol/g) was added to a manual SPPS vessel and bubbled with DMF (5 mL) for 15 min then the solvent was discharged. An allyl deprotection solution of tetrakis (triphenylphosphine) palladium (150 mg, 0.130 mmol) and phenylsilane (80.0 µL, 0.649 mmol) in DCM and DMF (1:1, 2 mL) was added. The solution was bubbled with argon for 2 h in darkness, after which the deprotection solution was discharged and the resin was washed with DMF (3 x 3 mL), 0.5 % sodium diethyldithiocarbamate solution in DMF (4 x 5 mL), DMF (4 x 5 mL) and DCM (4 x 5 mL). The resin was dried under

argon, then added to a 5 mL flame dried round bottom flask under argon. Dry dichloromethane (3 mL) was added and the suspension stirred for 15 min. Benzoyl chloride (8.00 µL, 68.9 µmol), triethylamine (20.0 µL, 0.143 mmol) and catalytic DMAP (1 crystal) were added and the reaction mixture was stirred overnight at 60°C. The resin was then filtered through a manual SPPS vessel and washed with DMF (3 x 5 mL) and DCM (3 x 5 mL) before being dried under argon. A small sample was cleaved using a 2 % TFA solution in DCM (1 mL). The cleavage cocktail was gently agitated for 1 h and filtered through a glass wool plug. The filtrate was concentrated with a gentle stream of argon before being analysed by LC-MS ($[M+H]^+$ calculated for $C_{30}H_{36}N_4O_8$ 567.2, found (LC-MS) 567.5). Following this modified Yamaguchi esterification, the synthesis of Ser9-brevicidine was completed using standard Fmoc SPPS protocols as described above. The peptide was cleaved from resin, precipitated and purified by RP-HPLC (See HPLC purification of synthetic peptides) following the procedure for synthesising brevicidine. Yield: 13 mg, 13% over 28 steps. HPLC retention time 22.1 min; $[M+2H]^{2+}$ calculated for $C_{73}H_{104}N_{18}O_{17}$ 753.3986, found (HR-MS) 753.3980

***Synthesis of Dap9-Brevicidine (5)***

The desired Fmoc-tetrapeptide was synthesized from Fmoc-Ser-OAll as described above, with Fmoc-Dap(Alloc) used in place of Fmoc-Thr. This resin-bound tetrapeptide (0.1 mmol, 0.13 mmol/g) was added to a manual SPPS vessel and bubbled with DMF (5 mL) for 15 min then the solvent was discharged. An allyl deprotection solution of tetrakis (triphenylphosphine) palladium (462 mg, 0.400 mmol) and phenylsilane (246 µL, 2.00 mmol) in DCM and DMF (1:1, 8 mL) was added. The solution was bubbled with argon for 2 h in darkness, after which the deprotection solution was discharged and the resin was washed with DMF (3 x 3 mL), 0.5 % sodium diethyldithiocarbamate solution in DMF (6 x 10 mL), DMF (4 x 5 mL) and DCM (4 x 5 mL). A solution of HATU (76.0 mg, 0.200 mmol) and DIPEA (70.0 µL, 0.402 mmol) in DMF (3 mL) and bubbled with argon overnight. The coupling solution was discharged, and the resin was washed with DMF (3 x 5 mL) then DCM (3 x 5 mL) and dried under a stream of argon. The synthesis of Dap9-brevicidine was completed using standard Fmoc SPPS protocols as described above. The peptide was cleaved from resin, precipitated and purified by RP-HPLC (See HPLC purification of synthetic peptides) following the procedure for synthesising brevicidine. Yield: 20 mg, 13% over 28 steps. HPLC retention time 21.6 min; $[M+2H]^{2+}$ calculated for $C_{73}H_{105}N_{19}O_{16}$ 752.9066, found (HR-MS) 752.9368.

***Synthesis of MeDap9-Brevicidine (7)***

The desired Fmoc-tetrapeptide was synthesized from Fmoc-Ser-OAll as described above, with Fmoc-MeDap(Alloc)-OH (IUPAC name: (2S,3R)-2-[[(9H-fluoren-9-ylmethoxy)carbonyl]amino]-3-[[(2-propen-1-yloxy)carbonyl]amino]-butanoic acid) used in place of Fmoc-Thr. The synthesis of Fmoc-MeDap(Alloc)-OH was adapted from a previously reported literature precedent (R. Moran Ramallal, R. Liz & V. Gotor, *J. Org. Chem.*, 2010, 75, 19, 6614-6624) - To a flame dried 25 mL round bottom flask was added Fmoc-L-Abu(3*R*-$N_3$)-OH (146 mg, 0.398 mmol) and 10% Pd/C (63 mg, 59.2 μmol). The flask was evacuated and a balloon of hydrogen was attached. Methanol (10 mL) was added and the suspension was stirred for 30 min before being filtered through a celite plug. The filtrate was concentrated under vacuum and re-dissolved in dichloromethane (10 mL). The solution was stirred at 0 °C then allyl chloroformate (51 μL, 0.478 mmol) and DIPEA (83 μL, 0.478 mmol) were added. The reaction was stirred at 0 °C for 3 h and concentrated under vacuum. The crude solid was subsequently purified by flash chromatography (1 % MeOH in DCM with 1% acetic acid). The fractions containing product were pooled, concentrated and co-evaporated with toluene (3 x 10 mL) then chloroform (3 x 10 mL) to yield a white solid (70 mg, 41%); TLC: $R_f$ 0.44 (10 % MeOH in DCM); $[\alpha]_D^{20}$: +31.9 (0.6, $CHCl_3$); $^1H$ NMR (400 MHz, $CDCl_3$): δ 7.74 (2H, d, $J$ = 7.5 Hz, Fmoc), 7.58 (2H, t, $J$ = 6.7 Hz, Fmoc), 7.38 (2H, t, $J$ = 7.3 Hz, Fmoc), 7.28 (2H, t, $J$ = 7.3 Hz, Fmoc), 6.09 (1H, d, $J$ = 7.7 Hz, -N**H**), 5.84 (1H, br, -$OCH_2C$**H**=$CH_2$), 5.32 (1H, d, $J$ = 6.7 Hz, -CHCH($CH_3$)N**H**-) 5.23 (1H, d, $J$ = 17.1 Hz, -$OCH_2CH$=C**$H_2$**), 5.15 (1H, d, $J$ = 9.9 Hz, -$OCH_2CH$=C**$H_2$**), 4.52 – 4.35 (6H, m, -C**H**CH($CH_3$)NH-, -CHC**H**($CH_3$)NH-, Fmoc-CHC**$H_2$**), 4.19 (1H, t, $J$ = 6.9 Hz, Fmoc-C**H**$CH_2$), 1.23 (3H, br, -CHCH(C**$H_3$**)NH-); $[M+H]^+$ calculated for, $C_{23}H_{24}N_2O_6$ 425.1707, found (LC-MS) 425.4.

This resin-bound tetrapeptide (0.05 mmol, 0.14 mmol/g) was added to a manual SPPS vessel and bubbled with DMF (5 mL) for 15 min then the solvent was discharged. An allyl deprotection solution of tetrakis (triphenylphosphine) palladium (231 mg, 0.200 mmol) and phenylsilane (123 μL, 0.998 mmol) in DCM and DMF (1:1, 6 mL) was added. The solution was bubbled with argon for 2 h in darkness, after which the deprotection solution was discharged and the resin was washed with DMF (3 x 3 mL), 0.5 % sodium diethyldithiocarbamate solution in DMF (6 x 10 mL), DMF (4 x 5 mL) and DCM (4 x 5 mL). A solution of HATU (95.0 mg, 0.250 mmol) and DIPEA (87.0 μL, 0.499 mmol) in DMF (3 mL) and bubbled with argon overnight at 50 °C. The coupling solution was discharged, and the resin was washed with DMF (3 x 5 mL) then DCM (3 x 5 mL) and dried under a stream of argon. A small sample was cleaved using a 2 % TFA solution in DCM (1 mL). The cleavage cocktail was gently agitated for 1 h and filtered through a glass wool plug. The filtrate was concentrated with a gentle stream of argon before being analysed by LC-MS ($[M+H]^+$ calculated for $C_{30}H_{36}N_4O_8$ 580.3, found (LC-MS) 580.6). The synthesis of MeDap9-brevicidine was completed

using standard Fmoc SPPS protocols as described above. The peptide was cleaved from resin, precipitated and purified by RP-HPLC (See HPLC purification of synthetic peptides) following the procedure for synthesising brevicidine. Yield: 3 mg, 2% over 28 steps. HPLC retention time 21.2 min; $[M+H]^+$ calculated for $C_{74}H_{107}N_{19}O_{16}$ 1518.8216, found (HR-MS) 1518.4032.

***Synthesis of Laterocidine (2)***

Rink Amide MBHA resin (5.0 g, 0.67 mmol $g^{-1}$) was loaded by overnight coupling via the free sidechain carboxylate of Fmoc-Asp-OAll (2.65 g, 6.70 mmol, 2 eq.) with BOP (2.96 g, 6.70 mmol, 2 eq.) and DiPEA (2.33 mL, 13.4 mmol, 4 eq.) in 150 mL of DMF. After capping with $AcO_2$ : pyridine (3 : 2, v/v) for 30 min the resin loading was determined to be 0.37 mmol $g^{-1}$ . The loaded resin (680 mg, 0.25 mmol) was treated with $Pd(PPh_3)_4$ (75 mg, 0.075 mmol, 0.3 eq.) and $PhSiH_3$ (0.75 mL, 7.5 mmol, 30 eq.) in DCM (ca. 15 mL) under nitrogen for 1 h. The resin was subsequently washed with DCM (5 × 10 mL), followed by a solution of diethyldithiocarbamic acid trihydrate sodium salt (5 mg $mL^{-1}$ in DMF, 5 × 10 mL), and DMF (5 × 10 mL). TFA·$H_2N$-Gly-OAll (115 mg, 0.5 mmol, 2 eq.) was then coupled using BOP (221 mg, 0.5 mmol, 2 eq.) and DiPEA (174 µL, 1.0 mmol, 4 eq.) under nitrogen flow for 1 h. The next 3 amino acids (Ile10, Thr9, Trp8) were coupled manually (1 h) under nitrogen flow via standard Fmoc solid-phase peptide synthesis (SPPS) (resin : Fmoc-AA : BOP : DiPEA, 1 : 4 : 4 : 8 molar eq.). DMF (5 mL) was used as solvent and Fmoc deprotections (2 min then 10 min) were carried out with 5 mL piperidine : DMF (1 : 4, v/v). The following Fmoc amino acids were used: Fmoc-Ile-OH, Fmoc-Thr-OH (used without side chain protection), and Fmoc-Trp(Boc)-OH. After coupling of Fmoc-Trp(Boc)-OH esterification of the Thr side chain was achieved by treating the resin-bound peptide with Alloc-Gly-OH (596 mg, 3.75 mmol, 15 eq.), DIC (0.59 mL, 3.75 mmol, 15 eq. ) and DMAP (15 mg, 0.13 mmol, 0.5 eq. ) in 8 mL DCM : DMF (3 : 1, v/v) for 18 h under nitrogen. The resin was treated with $Pd(PPh_3)_4$ (75 mg, 0.075 mmol) and $PhSiH_3$ (0.75 mL, 7.5 mmol) in DCM (ca. 15 mL) under nitrogen for 2 h before being washed with DCM (5 × 10 mL), followed by a solution of diethyldithiocarbamic acid trihydrate sodium salt (5 mg $mL^{-1}$ in DMF, 5 × 10 mL), and DMF (5 × 10 mL). The peptide was then cyclized using BOP (442 mg, 1.0 mmol, 4 eq. ) and DiPEA (0.35 mL, 2.0 mmol, 8 eq.) for 2 h in 5 mL DMF under nitrogen flow. The remaining linear N-terminal section of the peptide was then synthesized using the standard SPPS protocol mentioned above. The following Fmoc amino acids were used: Fmoc-D-Ser(tBu)-OH, Fmoc-D-Tyr(tBu)-OH, Fmoc-D-Trp(Boc)-OH, Fmoc-D-Orn(Boc)-OH, Fmoc-L-Orn(Boc)-OH, Fmoc-Gly-OH. Following the final Fmoc removal step, isopelargonic acid (79 mg, 0.5 mmol, 2 eq.) was coupled using BOP (221 mg, 0.5 mmol, 2 eq.) and

DiPEA (174 μL, 1.0 mmol, 4 eq.) in 5 mL of DMF overnight, under nitrogen flow. Final deprotection was carried out by treating the resin with TFA : TIS : $H_2O$ (95 : 2.5 : 2.5, 10 mL) for 90 min. The reaction mixture was filtered through cotton, the filtrate precipitated in MTBE : petroleum ether (1 : 1), and the resulting precipitate washed once more with MTBE : petroleum ether (1 : 1). The crude cyclic peptide was lyophilized from tBuOH : $H_2O$ (1 : 1) and purified with reverse phase HPLC (See HPLC purification of synthetic peptides). Pure fractions were pooled and lyophilized to yield laterocidine in >95% purity as a white powder. Yield: 8 mg, 2% over 29 steps. $[M+2H]^{2+}$ calculated for, $C_{78}H_{113}N_{19}O_{18}$ 802,9329, found (HR-MS) 802,9326.

***Synthesis of ent-Laterocidine (ent-2)***

Rink Amide MBHA resin (5.0 g, 0.67 mmol $g^{-1}$) was loaded by overnight coupling via the free sidechain carboxylate of Fmoc-D-Asp-OAll (2.65 g, 6.70 mmol, 2 eq.) with BOP (2.96 g, 6.70 mmol, 2 eq.) and DiPEA (2.33 mL, 13.4 mmol, 4 eq.) in 150 mL of DMF. After capping with $AcO_2$ : pyridine (3 : 2, v/v) for 30 min the resin loading was determined to be 0.37 mmol $g^{-1}$ . The loaded resin (675 mg, 0.25 mmol) was treated with $Pd(PPh_3)_4$ (75 mg, 0.075 mmol, 0.3 eq.) and $PhSiH_3$ (0.75 mL, 7.5 mmol, 30 eq.) in DCM (ca. 15 mL) under nitrogen for 1 h. The resin was subsequently washed with DCM (5 × 10 mL), followed by a solution of diethyldithiocarbamic acid trihydrate sodium salt (5 mg $mL^{-1}$ in DMF, 5 × 10 mL), and DMF (5 × 10 mL). TFA·$H_2N$-Gly-OAll (115 mg, 0.5 mmol, 2 eq.) was then coupled using BOP (221 mg, 0.5 mmol, 2 eq.) and DiPEA (174 μL, 1.0 mmol, 4 eq.) under nitrogen flow for 1 h. The next 3 amino acids (D-Ile10, D-Thr9, D-Trp8) were coupled manually (1 h) under nitrogen flow via standard Fmoc solid-phase peptide synthesis (SPPS) (resin : Fmoc-AA : BOP : DiPEA, 1 : 4 : 4 : 8 molar eq.). DMF (5 mL) was used as solvent and Fmoc deprotections (2 min then 10 min) were carried out with 5 mL piperidine : DMF (1 : 4, v/v). The following Fmoc amino acids were used: Fmoc-D-Ile-OH, Fmoc-D-Thr-OH (used without side chain protection), and Fmoc-D-Trp(Boc)-OH. After coupling of Fmoc-D-Trp(Boc)-OH esterification of the D-Thr side chain was achieved by treating the resin-bound peptide with Alloc-Gly-OH (596 mg, 3.75 mmol, 15 eq.), DIC (0.59 mL, 3.75 mmol, 15 eq. ) and DMAP (15 mg, 0.13 mmol, 0.5 eq. ) in 8 mL DCM : DMF (3 : 1, v/v) for 18 h under nitrogen. The resin was treated with $Pd(PPh_3)_4$ (75 mg, 0.075 mmol) and $PhSiH_3$ (0.75 mL, 7.5 mmol) in DCM (ca. 15 mL) under nitrogen for 2 h before being washed with DCM (5 × 10 mL), followed by a solution of diethyldithiocarbamic acid trihydrate sodium salt (5 mg $mL^{-1}$ in DMF, 5 × 10 mL), and DMF (5 × 10 mL). The peptide was then cyclized using BOP (442 mg, 1.0 mmol, 4 eq. ) and DiPEA (0.35 mL, 2.0 mmol, 8 eq.) for 2 h in 5 mL DMF under nitrogen flow. The remaining linear N-terminal section of the peptide was then synthesized using the standard SPPS protocol mentioned above. The following Fmoc amino acids were used: Fmoc-L-Ser(tBu)-OH, Fmoc-L-Tyr(tBu)-OH,

Fmoc-L-Trp(Boc)-OH, Fmoc-L-Orn(Boc)-OH, Fmoc-D-Orn(Boc)-OH, Fmoc-Gly-OH. Following the final Fmoc removal step, isopelargonic acid (79 mg, 0.5 mmol, 2 eq.) was coupled using BOP (221 mg, 0.5 mmol, 2 eq.) and DiPEA (174 µL, 1.0 mmol, 4 eq.) in 5 mL of DMF overnight, under nitrogen flow. Final deprotection was carried out by treating the resin with TFA : TIS : $H_2O$ (95 : 2.5 : 2.5, 10 mL) for 90 min. The reaction mixture was filtered through cotton, the filtrate precipitated in MTBE : petroleum ether (1 : 1), and the resulting precipitate washed once more with MTBE : petroleum ether (1 : 1). The crude cyclic peptide was lyophilized from tBuOH : $H_2O$ (1 : 1) and purified with reverse phase HPLC (See <u>HPLC purification of synthetic peptides).</u> Pure fractions were pooled and lyophilized to yield *ent*-laterocidine in >95% purity as a white powder. Yield: 30 mg, 7.4% over 29 steps. $[M+2H]^{2+}$ calculated for, $C_{78}H_{113}N_{19}O_{18}$ 802,9329, found (HR-MS) 802,9327.

***Synthesis of Ser9-Laterocidine (4)***

The loaded resin (274 mg, 0.1 mmol) was treated with $Pd(PPh_3)_4$ (30 mg, 0.03 mmol, 0.3 eq.) and $PhSiH_3$ (0.30 mL, 3.0 mmol, 30 eq.) in DCM (ca. 7 mL) under nitrogen for 1 h. The resin was subsequently washed with DCM (5 × 10 mL), followed by a solution of diethyldithiocarbamic acid trihydrate sodium salt (5 mg $mL^{-1}$ in DMF, 5 × 10 mL), and DMF (5 × 10 mL). TFA·$H_2N$-Gly-OAll (115 mg, 0.5 mmol, 2 eq.) was then coupled using BOP (88 mg, 0.2 mmol, 2 eq.) and DiPEA (87 µL, 0.4 mmol, 4 eq.) under nitrogen flow for 2 h. The next 3 amino acids (Ile10, Ser9, Trp8) were coupled manually (1 h) under nitrogen flow via standard Fmoc solid-phase peptide synthesis (SPPS) (resin : Fmoc-AA : BOP : DiPEA, 1 : 4 : 4 : 8 molar eq.). Dry DMF (3 mL) was used as solvent and Fmoc deprotections (2 min then 10 min) were carried out with 3 mL piperidine : DMF (1 : 4, v/v). The following Fmoc amino acids were used: Fmoc-Ile-OH, Fmoc-Ser-OH (used without side chain protection), and Fmoc-Trp(Boc)-OH. After coupling of Fmoc-Trp(Boc)-OH esterification of the Ser side chain was achieved by treating the resin-bound peptide with Alloc-Gly-OH (238 mg, 1.5 mmol, 15 eq.), DIC (0.24 mL, 1.5 mmol, 15 eq. ) and DMAP (6 mg, 0.05 mmol, 0.5 eq. ) in 3 mL DCM : DMF (3 : 1, v/v) for 18 h under nitrogen. The resin was treated with $PhSiH_3$ (0.30 mL, 3.0 mmol, 30 eq.) in DCM (ca. 7 mL) under nitrogen for 2 h before being washed with DCM (5 × 10 mL), followed by a solution of diethyldithiocarbamic acid trihydrate sodium salt (5 mg $mL^{-1}$ in DMF, 5 × 10 mL), and DMF (5 × 10 mL). The peptide was then cyclized using BOP (177 mg, 0.4 mmol, 4 eq. ) and DiPEA (0.14 mL, 0.8 mmol, 8 eq.) for 2 h in 3 mL DMF under nitrogen flow. The remaining linear N-terminal section of the peptide was then synthesized using the standard SPPS protocol mentioned above. The following Fmoc amino acids were used: Fmoc-D-Ser(tBu)-OH, Fmoc-D-Tyr(tBu)-OH, Fmoc-D-Trp(Boc)-OH, Fmoc-D-Orn(Boc)-OH, Fmoc-L-Orn(Boc)-OH, Fmoc-Gly-OH. Following the final Fmoc removal step, isopelargonic acid (79 mg,

0.5 mmol, 2 eq.) was coupled using BOP (88 mg, 0.2 mmol, 2 eq.) and DiPEA (87 μL, 0.4 mmol, 4 eq.) in 3 mL of DMF overnight, under nitrogen flow. Final deprotection was carried out by treating the resin with TFA : TIS : $H_2O$ (95 : 2.5 : 2.5, 5 mL) for 90 min. The reaction mixture was filtered through cotton, the filtrate precipitated in MTBE : petroleum ether (1 : 1), and the resulting precipitate washed once more with MTBE : petroleum ether (1 : 1). The crude cyclic peptide was lyophilized from tBuOH : $H_2O$ (1 : 1) and purified with reverse phase HPLC (See <u>HPLC purification of synthetic peptides</u>). Pure fractions were pooled and lyophilized to yield laterocidine in >95% purity as a white powder. Yield: 4 mg, 2% over 29 steps $[M+2H]^{2+}$ calculated for, $C_{78}H_{113}N_{19}O_{18}$ 795.9250, found (HR-MS) 795,9249.

***Synthesis of Dap9-Laterocidine (6)***

The loaded resin (680 mg, 0.25 mmol) was treated with $Pd(PPh_3)_4$ (75 mg, 0.075 mmol, 0.3 eq.) and $PhSiH_3$ (0.75 mL, 7.5 mmol, 30 eq.) in DCM (ca. 15 mL) under nitrogen for 1 h. The resin was subsequently washed with $CH_2Cl_2$ (5 × 10 mL), followed by a solution of diethyldithiocarbamic acid trihydrate sodium salt (5 mg $mL^{-1}$ in DMF, 5 × 10 mL), and DMF (5 × 10 mL). TFA□$H_2N$-Gly-Gly-OAll (143 mg, 0.5 mmol, 2 eq.) was then coupled using BOP (221 mg, 0.5 mmol, 2 eq.) and DiPEA (174 μL, 1.0 mmol, 4 eq.) under nitrogen flow for 1 h. The next two amino acids (Ile and Dap) were coupled manually (1 h) under nitrogen flow via standard Fmoc solid-phase peptide synthesis (SPPS) (resin : Fmoc-AA : BOP : DiPEA, 1 : 4 : 4 : 8 molar eq.). DMF (5 mL) was used as solvent and Fmoc deprotections (2 min then 10 min) were carried out with 5 mL piperidine : DMF (1 : 4, v/v). The following Fmoc amino acids were used: Fmoc-Ile-OH and Fmoc-Dap(Alloc)-OH. The resin was then treated two times with $Pd(PPh_3)_4$ (75 mg, 0.075 mmol) and $PhSiH_3$ (0.75 mL, 7.5 mmol) in DCM (ca. 15 mL) under nitrogen for 3 h with washing in between with DCM (5 × 10 mL), followed by a solution of diethyldithiocarbamic acid trihydrate sodium salt (5 mg $mL^{-1}$ in DMF, 5 × 10 mL), and DMF (5 × 10 mL). The macrocycle was then closed by treatment with BOP (442 mg, 1.0 mmol, 4 eq. ) and DiPEA (0.35 mL, 2.0 mmol, 8 eq.) for 2 h in 5 mL DMF under nitrogen flow. Following cyclization the remaining linear N-terminal section of the peptide was added using a CEM Liberty Blue automated peptide synthesizer with microwave irradiation, on standard settings (resin : Fmoc-AA : DIC : Oxyma, 1 : 5 : 5 : 5 molar eq.). DMF was used as solvent and Fmoc deprotections were carried out with piperidine : DMF (1 : 4, v/v). The following Fmoc amino acids were used: Fmoc-D-Ser(tBu)-OH, Fmoc-D-Tyr(tBu)-OH, Fmoc-D-Trp(Boc)-OH, Fmoc-L-Orn(Boc)-OH, Fmoc-L-Orn(Boc)-OH, Fmoc-Gly-OH and Fmoc-Trp(Boc)-OH. Following the final Fmoc removal step, the resin was removed from the CEM Liberty Blue and washed with DCM and DMF before isopelargonic acid (79 mg, 0.5 mmol, 2 eq.) was coupled manually using BOP (221 mg, 0.5 mmol, 2 eq.) and DiPEA (174 μL, 1.0 mmol, 4 eq.) in 5 mL of

DMF overnight, under nitrogen flow. Final deprotection was carried out by treating the resin with TFA : TIS : $H_2O$ (95 : 2.5 : 2.5, 10 mL) for 90 min. The reaction mixture was filtered through cotton, the filtrate was precipitated in MTBE : petroleum ether (1 : 1) and the resulting precipitate washed once more with MTBE : petroleum ether (1 : 1). The crude cyclic peptide was lyophilized from tBuOH : $H_2O$ (1 : 1) and purified with reverse phase HPLC (See HPLC purification of synthetic peptides). Pure fractions were pooled and lyophilized to yield Dap9-laterocidamide in >95% purity as a white powder. Yield 10 mg, 3 % yield over 27 steps. $[M+2H]^{2+}$ calculated for, $C_{77}H_{112}N_{20}O_{17}$ 795,4330 found (HR-MS) 795,43

***Synthesis of Dap9-Laterocidine (6) via Solution-Phase Cyclization.***

2-Chlorotrityl resin (5.0 g, 1.60 mmol g−1 ) was loaded with Fmoc-Gly-OH. Resin loading was determined to be 0.67 mmol $g^{-1}$. The linear peptide was assembled manually on a 3 mmol scale under nitrogen flow via standard Fmoc solid-phase peptide synthesis (SPPS) (1 h couplings, resin : Fmoc-AA : BOP : DiPEA, 1 : 4 : 4 : 8 molar eq.). DMF (60 mL) was used as solvent and Fmoc deprotections (2 min then 10 min) were carried out with 60 mL piperidine : DMF (1 : 4, v/v). The following Fmoc amino acids were used: Fmoc-D-Ser(tBu)-OH, Fmoc-D-Tyr(tBu)-OH, Fmoc-D-Trp(Boc)-OH, Fmoc-L-Orn(Boc)-OH, Fmoc-L-Orn(Boc)-OH, Fmoc-Gly-OH and Fmoc-Trp(Boc)-OH, Fmoc-Dap(Alloc)-OH and Fmoc-Ile-OH. Following the final Fmoc removal step, isopelargonic acid (0.95 g, 6 mmol, 2 eq.) was coupled using BOP (2.65 g, 6 mmol, 2 eq.) and DiPEA (2.1 mL, 12 mmol, 4 eq.) in 60 mL of DMF overnight, under nitrogen flow. The resin was then treated three times with $Pd(PPh_3)_4$ (0.9 g, 0.075 mmol) and $PhSiH_3$ (9.0 mL, 7.5 mmol) in $CH_2Cl_2$ (ca. 180 mL) under nitrogen for 2 h with washing in between with $CH_2Cl_2$ (5 × 120 mL), followed by a solution of diethyldithiocarbamic acid trihydrate sodium salt (5 mg $mL^{-1}$ in DMF, 5 × 120 mL), and DMF (5 × 120 mL). The peptide was cleaved off the resin by treating it with HFIP : DCM (1 : 4, v/v, 240 mL) for 1 h and rinsed with additional HFIP : DCM and DCM. The combined washings were then evaporated to yield the linear protected peptide with a free C-terminus and Dap amino sidechain. The partially protected peptide was dissolved in DCM (1.8 L), treated with BOP (2.65 g, 6 mmol, 2 eq.) and DiPEA (2.1 mL, 12 mmol, 4 eq.) and the solution was stirred overnight under nitrogen atmosphere. The reaction mixture was concentrated and directly treated with TFA : TIS : $H_2O$ (95 : 2.5 : 2.5, 120 mL) for 90 min. The reaction mixture was subsequently filtered through cotton, the filtrate was precipitated in MTBE : petroleum ether (1 : 1) and the resulting precipitate washed once more with MTBE : petroleum ether (1 : 1). The crude cyclic peptide was lyophilized from tBuOH : $H_2O$ (1 : 1) and purified using a Buchi Pure C-815 Flash system with a Buchi FlashPure ID C18-WP 20 µm irregular 80 g reverse-phase column. The purification was performed while doing 1 g injections of crude peptide. The following solvent system, at a flow rate of 40 mL/min, was used: solvent A, 0.1 % TFA in water; solvent B, 0.1 % TFA in acetonitrile.

Gradient elution was as follows: 100:0 to 50:50 (A/B) over 60 min. Pure fractions were pooled and lyophilized to yield Dap9-laterocidamide in >95% purity as a white powder. Yield 1.4 g, 29 % yield over 30 steps. $[M+2H]^{2+}$ calculated for, $C_{77}H_{112}N_{20}O_{17}$ 795,4330 found (HR-MS) 795,4327.

***Synthesis of MeDap9-Laterocidine (8)***

Rink amide MBHA resin loaded with Fmoc-Asp-OAll (680 mg, 0.25 mmol) was was treated with $Pd(PPh_3)_4$ (75 mg, 0.075 mmol) and $PhSiH_3$ (0.75 mL, 7.5 mmol) in DCM (ca. 15 mL) under nitrogen for 2 h before being washed with DCM (5 × 10 mL), followed by a solution of diethyldithiocarbamic acid trihydrate sodium salt (5 mg $mL^{-1}$ in DMF, 5 × 10 mL), and DMF (5 × 10 mL). TFA·$H_2N$-Gly-OAll (115 mg, 0.5 mmol, 2 eq.) was then coupled using BOP (221 mg, 0.5 mmol, 2 eq.) and DiPEA (174 µL, 1.0 mmol, 4 eq.) under nitrogen flow for 1 h. The next 2 amino acids Fmoc-Ile-OH and (2S,3R)-Fmoc-azido-aminobutyric acid were coupled manually (1 h) under nitrogen flow via standard Fmoc solid-phase peptide synthesis (SPPS) (resin : Fmoc-AA : BOP : DiPEA, 1 : 4 : 4 : 8 molar eq.). DMF (5 mL) was used as solvent and Fmoc deprotections (2 min then 10 min) were carried out with 5 mL piperidine : DMF (1 : 4, v/v). The azide was then reduced using a DTT (2M) and DiPEA (1M) in DMF (ca. 15 mL) for 2H under $N_2$ followed by washings with DMF (5×10 mL). The Allyl protecting group on the C-terminus was then removed using $Pd(PPh_3)_4$ (75 mg, 0.075 mmol) and $PhSiH_3$ (0.75 mL, 7.5 mmol) in DCM (ca. 15 mL) under nitrogen for 2 h before being washed with DCM (5 × 10 mL), followed by a solution of diethyldithiocarbamic acid trihydrate sodium salt (5 mg $mL^{-1}$ in DMF, 5 × 10 mL), and DMF (5 × 10 mL). The peptide was then cyclized using BOP (442 mg, 1.0 mmol, 4 eq. ) and DiPEA (0.35 mL, 2.0 mmol, 8 eq.) for 2 h in 5 mL DMF under nitrogen flow. The remaining linear N-terminal section of the peptide was then synthesized using the standard SPPS protocol mentioned above. The following Fmoc amino acids were used: Fmoc-D-Ser(tBu)-OH, Fmoc-D-Tyr(tBu)-OH, Fmoc-D-Trp(Boc)-OH, Fmoc-D-Orn(Boc)-OH, Fmoc-L-Orn(Boc)-OH, Fmoc-Gly-OH. Fmoc-L-Trp(Boc)-OH. Following the final Fmoc removal step, isopelargonic acid (79 mg, 0.5 mmol, 2 eq.) was coupled using BOP (221 mg, 0.5 mmol, 2 eq.) and DiPEA (174 µL, 1.0 mmol, 4 eq.) in 5 mL of DMF overnight, under nitrogen flow. Final deprotection was carried out by treating the resin with TFA : TIS : $H_2O$ (95 : 2.5 : 2.5, 10 mL) for 90 min. The reaction mixture was filtered through cotton, the filtrate precipitated in MTBE : petroleum ether (1 : 1), and the resulting precipitate washed once more with MTBE : petroleum ether (1 : 1). The crude cyclic peptide was lyophilized from tBuOH : $H_2O$ (1 : 1) and purified with reverse phase HPLC (See HPLC purification of synthetic peptides). Pure fractions were pooled and lyophilized to yield MeDap9-laterocidine in (9.7 mg, 2.4% over 29 steps, purity >95%) $[M+2H]^{2+}$ calculated for, $C_{78}H_{114}N_{20}O_{17}$ 802.4408, found (HRMS) 802.4412.

***MIC determinations***

Minimum inhibitory concentrations (MICs) were determined by broth microdilution according to CLSI guidelines in triplicate. Blood agar plates were inoculated with glycerol stocks of *E. coli* ATCC 25922, *E. coli* MCR-1 (clinical isolate from Utrecht Medical Centre, NL), *E. coli* EQAS MCR-2 (clinical isolate from Wageningen University and Research, NL), *K. pneumoniae* ATCC 11228, *K. pneumoniae* ATCC 13883, *K. pneumonia* 2048 (clnical isolate from Vrije Universiteit Amsterdam Medical Centre, NL), *K. pneumonia* JS-123 (clinical isolate from from Utrecht Medical Centre, NL), *A. baumannii* ATCC 17961, *A. baumannii* ATCC 17978, *A. baumannii* 2018-006 (clinical isolate from Rijksinstituut voor Volksgezondheid en Milieu, NL), *A. baumannii* MDR (clinical isolate from Vrije Universiteit Amsterdam Medical Centre, NL), *P. aeruginosa* ATCC 27853, *P. aeruginosa* PAO1, *P. aeruginosa* NRZ-03961 (Reference strain from Das Nationale Referenzzentrum für gramnegative Krankenhauserreger, DE), *P. aeruginosa* M-120 (clinical isolate from Leiden University Medical Centre, NL) and *S. aureus* USA300 (clinical isolate from Texas Children's Hospital, USA). *E. coli* 25922 MCR1 was grown on LB agar supplemented with kanamycin. The inoculated agar plates were then incubated for 16 h at 37°C. Individually grown colonies were subsequently used to inoculate 3 mL aliquots of TSB that were then incubated at 37°C with shaking at 220 rpm. In parallel, the lipopeptide antibiotics to be assessed were serially diluted with Mueller-Hinton broth (MHB) in polypropylene 96-well plates (50 µL in each well). Once the $OD_{600}$ of the bacterial suspensions reached 0.5, the bacteria were diluted with MHB (final concentration $2 \times 10^5$ CFU $mL^{-1}$) and added to the microplates containing the test compounds (50 µL to each well, final volume: 100 µL). The well-plates were sealed with an adhesive membrane and after 16 h of incubation at 37°C with shaking at 220 rpm the wells were visually inspected for bacterial growth. MIC values reported are based on three technical replicates and defined as the lowest concentration of the compound that prevented visible growth of bacteria.

***In vivo tolerability and efficacy studies***

**Ethical Issues.** Animal experiments were performed under UK Home Office Licences P89653310 (tolerability and PK) and PA67E0BAA (thigh efficacy), with local ethical committee clearance.

**Animal Strain.** Mice used in these studies were supplied by Charles River (Margate UK) and were specific pathogen free. The strain of mice used was ICR (also known as CD1 Mice) which is a well characterized outbred murine strain. Mice (male) were 11-15 g on receipt and were allowed to acclimatise for at least 7 days.

**Animal Housing.** Mice were housed in sterilised individual ventilated cages exposing the mice at all times to HEPA filtered sterile air. Mice had free access to food and water and had aspen chip bedding (changed at least once weekly). The room temperature was 22°C +/- 1°C, with a relative humidity of 60% and maximum background noise of 56 dB. Mice were exposed to 12 h light/dark cycles.

**Test compounds.** Compound **6** was dissolved in water for injection in which it formed a clear colourless solution. Polymyxin B was dissolved in saline for injection to produce a clear colourless solution .

**Tolerability study.** The tolerability of compound **6** was assessed in the same mouse strain used for the efficacy studies. Compound 6 was administered via subcutaneous administration route at 3 8-h intervals indicating good tolerability up to 40 mg/kg. The mice used in the tolerability study were naïve and were not immunosuppressed or infected.

**Efficacy study.** The in *vivo* efficacy of compound **6** was assessed in a mouse thigh abscess model where both thighs of each mouse were infected with *E. coli* ATCC 25922

**Immunosuppression.** Mice were rendered neutropenic with subcutaneous injections of cyclophosphamide at 150 mg/kg 4 days before infection and 100 mg/kg 1 day before infection. The immunosuppression regime leads to neutropenia starting 24 h post administration of the first injection, which continues throughout the study.

**Infection.** The bacterial strain used was *E. coli* ATCC 25922. An aliquot of a previously prepared frozen stock of the strain was thawed and diluted in sterile PBS to the desired inoculum just prior to infection. Mice were infected with 0.05 mL of the bacterial strain suspensions by intramuscular (IM) injection under temporary inhaled anaesthesia (2.5% isofluorane for 3-4 min) into both thighs. The inoculum was 6 x $10^6$ cfu/mL, 3 x $10^5$ cfu/thigh.

**Analgesia.** At the time of thigh infection, buprenorphine analgesia was administered at 0.03mg/kg subcutaneously using a 0.015mg/mL solution delivered at 2 mL/kg. The same dose was administered again 9 and 17 h post-infection.

**Treatment.** Compound **6** was administered SC every 8 h starting 1 h post-infection at does of 10, 20, and 40 mg/kg. Additional control groups comprising an infected pre-treatment group, which was euthanised 1 h after infection, a vehicle (WFI) treated group and a group that received comparator Polymyxin B SC every 8 h dosed at 20 mg/kg were included.

**Endpoints.** One h and 20.5 (planned 25) h post-infection, the clinical condition of all animals was assessed prior to humane euthanasia using pentobarbitone overdose, and the thighs were removed and weighed. Thigh samples were homogenized in 3mL ice cold sterile PBS; the homogenates were quantitatively cultured onto CLED agar and incubated at 37°C for 18 - 24 h before colonies were counted.

**Data analysis.** The data from the culture burdens were analysed using appropriate non-parametric statistical models (Kruskal-Wallis using Conover-Inman to make all pairwise comparisons between groups) with StatsDirect software v. 3.3.5, and compared to vehicle control. For all calculations the thighs from each animal were treated as two separate data points.

## *Analytical RP-HPLC Data*

Analytical HPLC traces of brevicidine and analogues were obtained using RP-HPLC Method **B**. The crude trace of brevicidine was obtained using RP-HPLC Method **A**.

### Brevicidine (1)

*Crude*

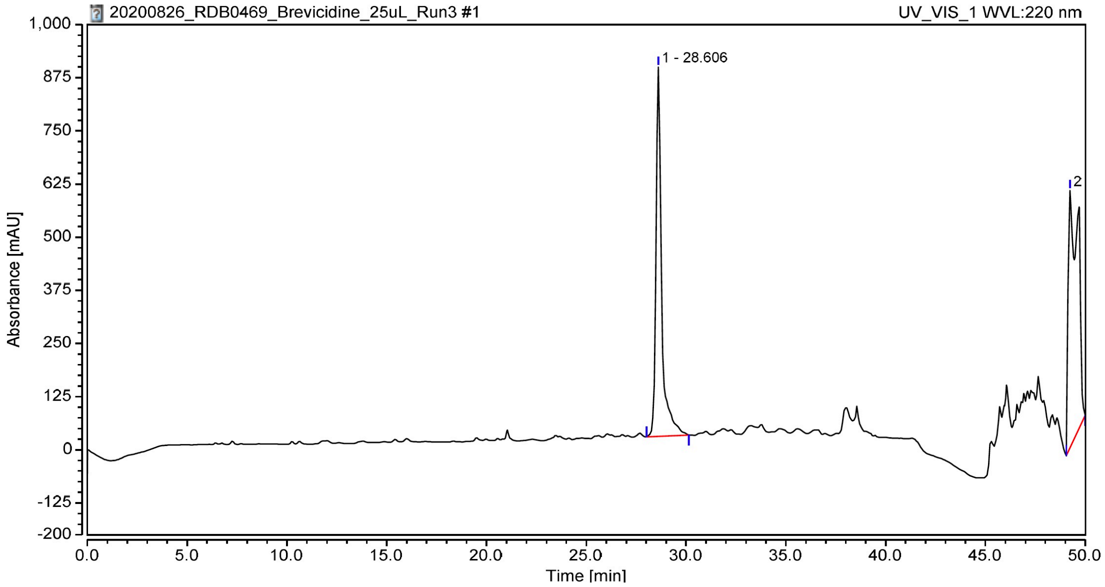


*Purified*

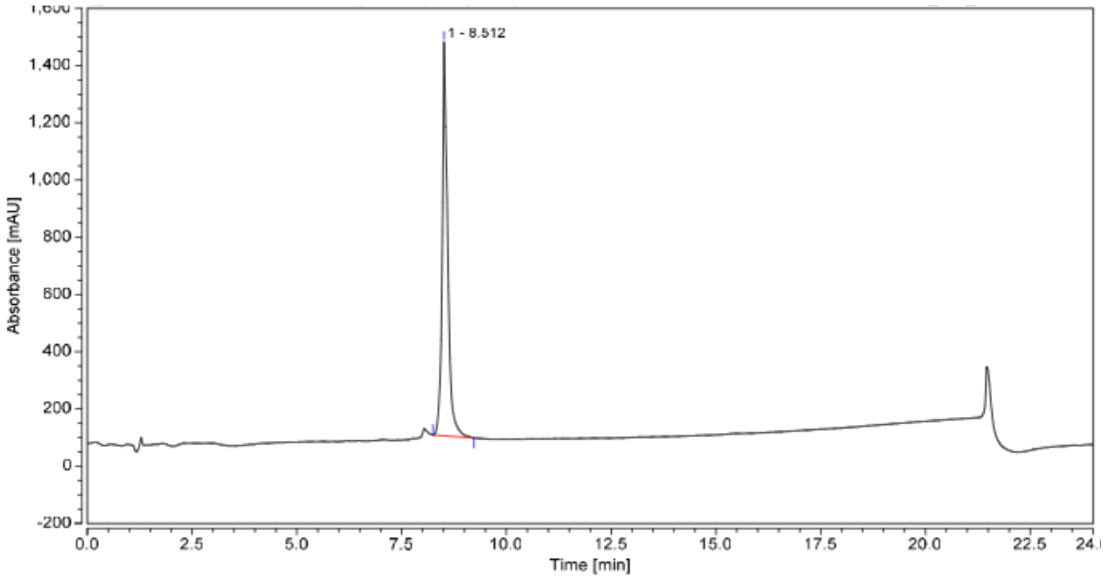

**ent-Brevicidine (ent-1)**

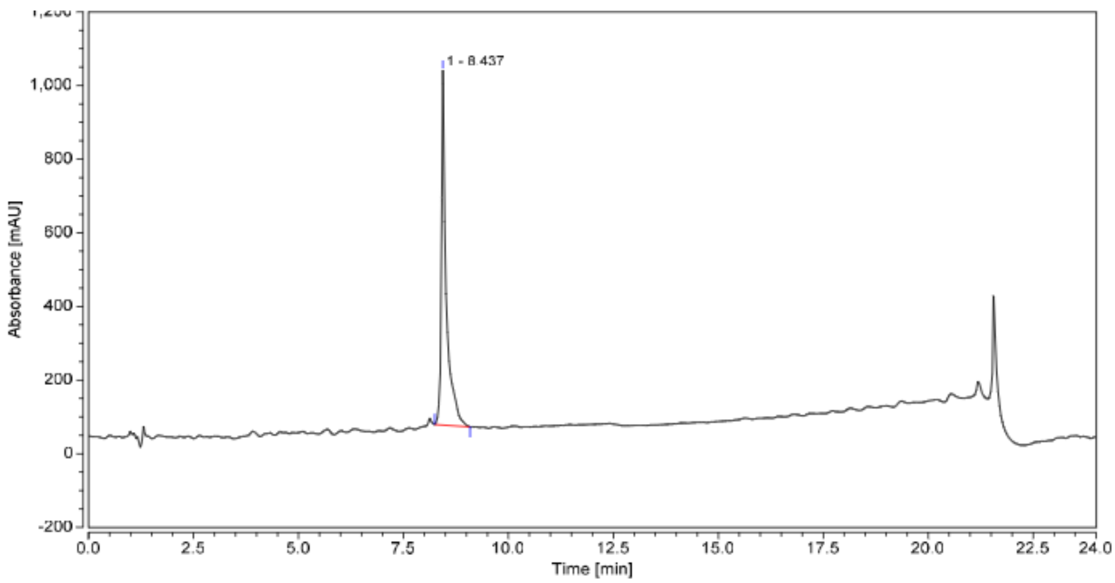


**Ser9-Brevicidine (3)**

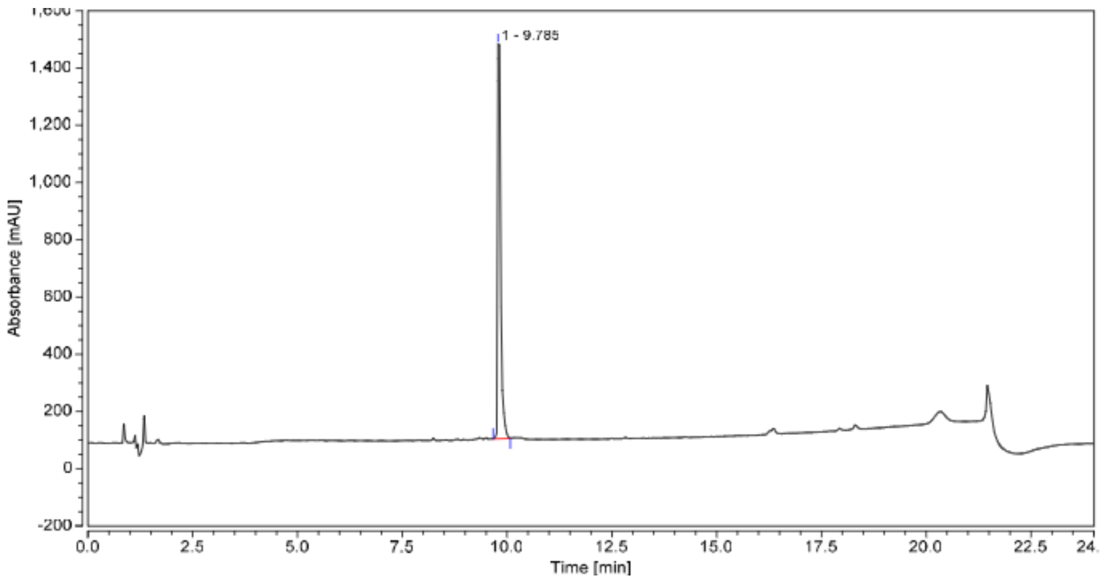

**Dap9-Brevicidine (5)**

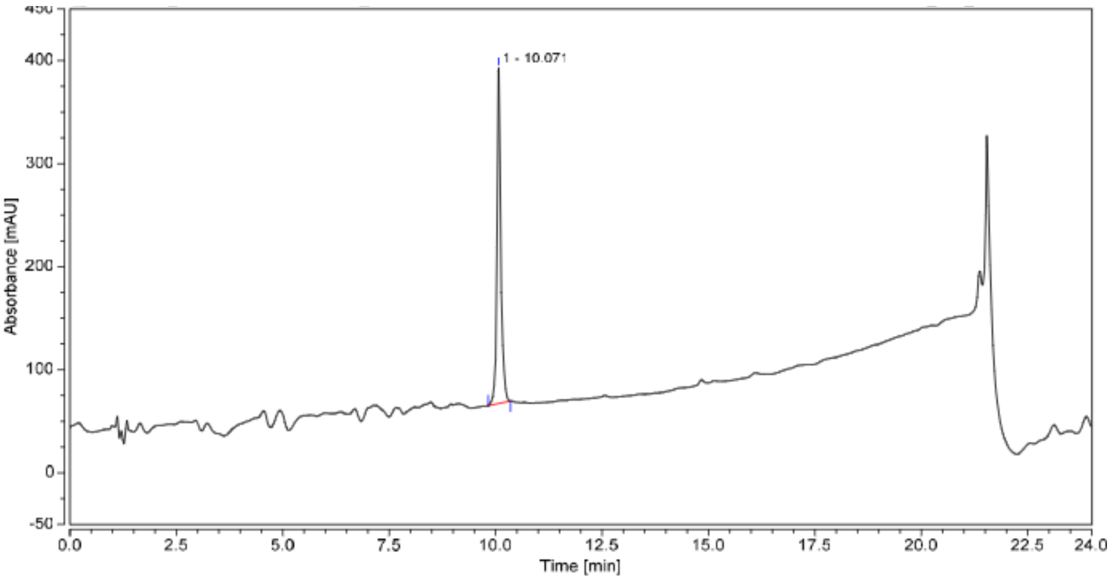


**MeDap9-Brevicidine (7)**

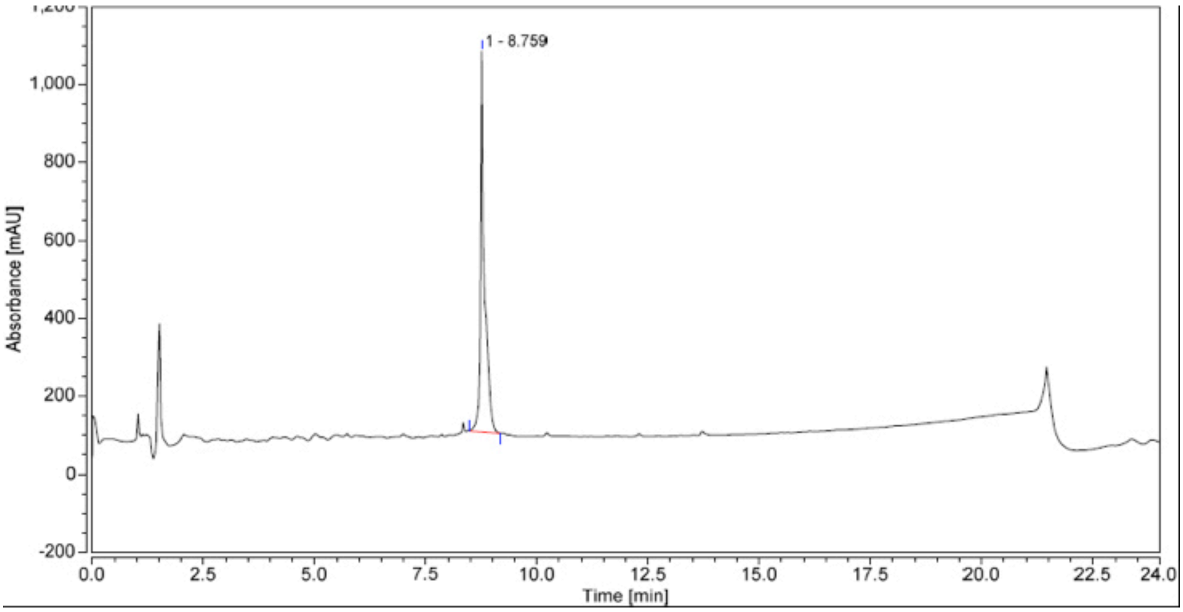

Analytical HPLC traces of laterocidine and analogues were obtained using RP-HPLC Method **D**. The crude trace of brevicidine was obtained using RP-HPLC Method **C**.

**Laterocidine (2)**

*Crude*

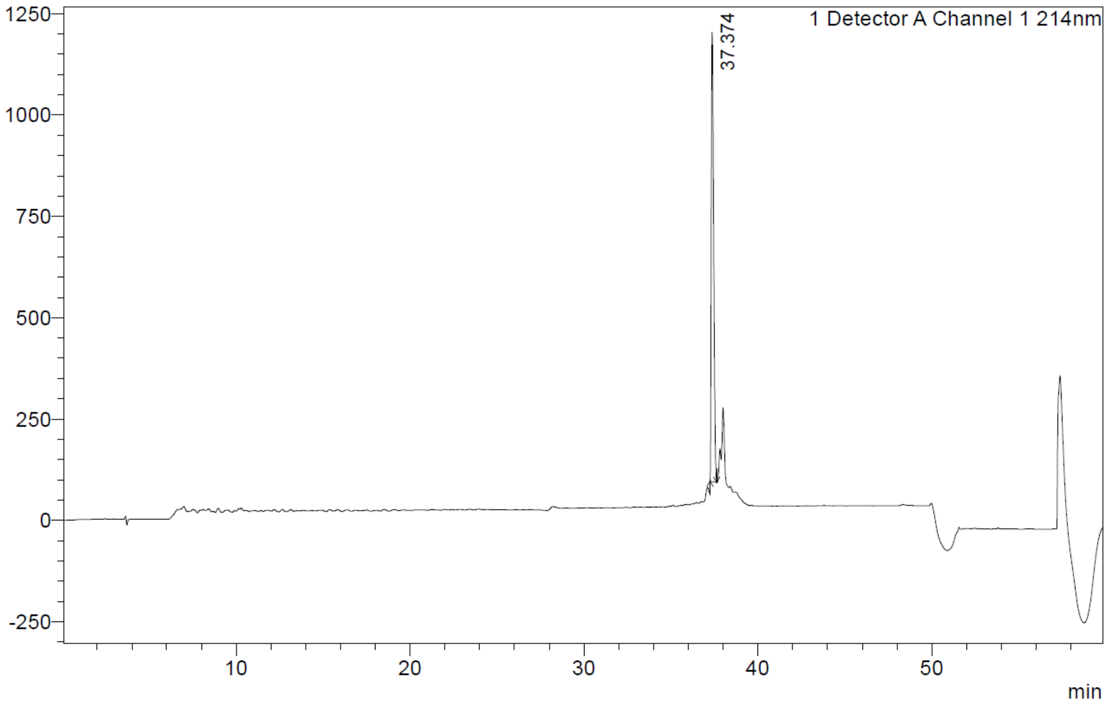


*Purified*

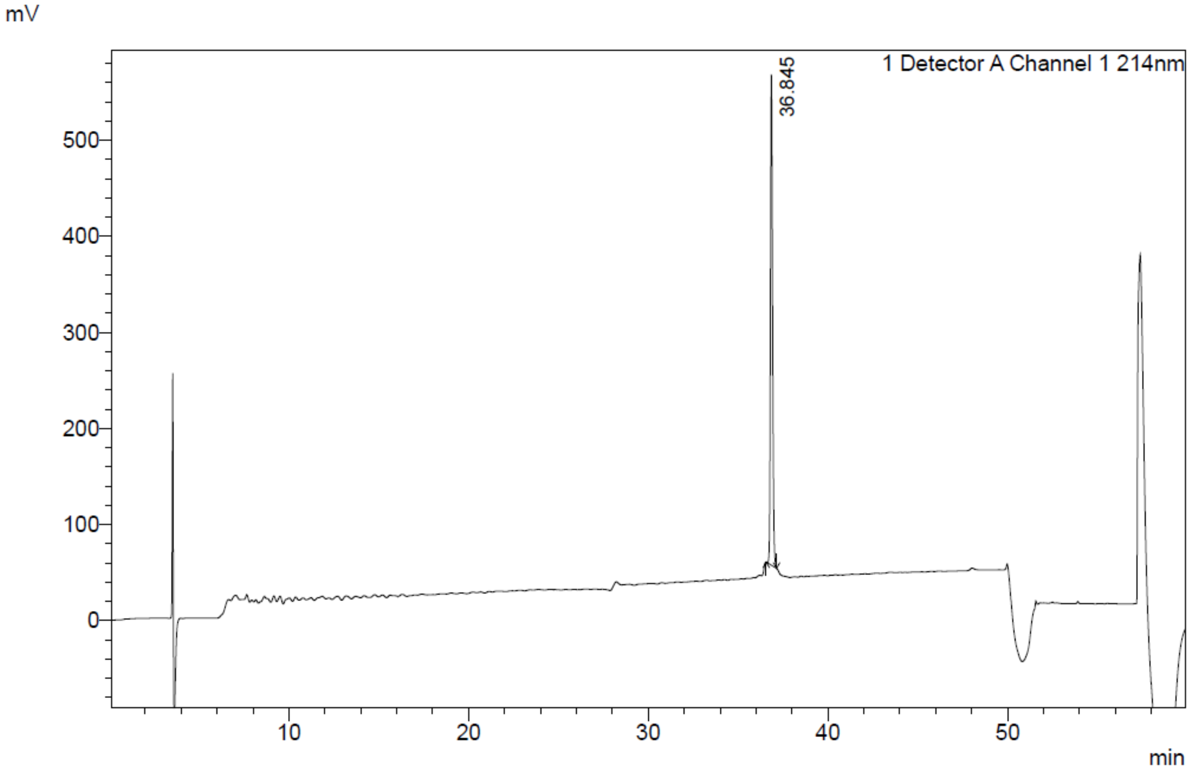

*ent*-**Laterocidine (*ent*-2)**

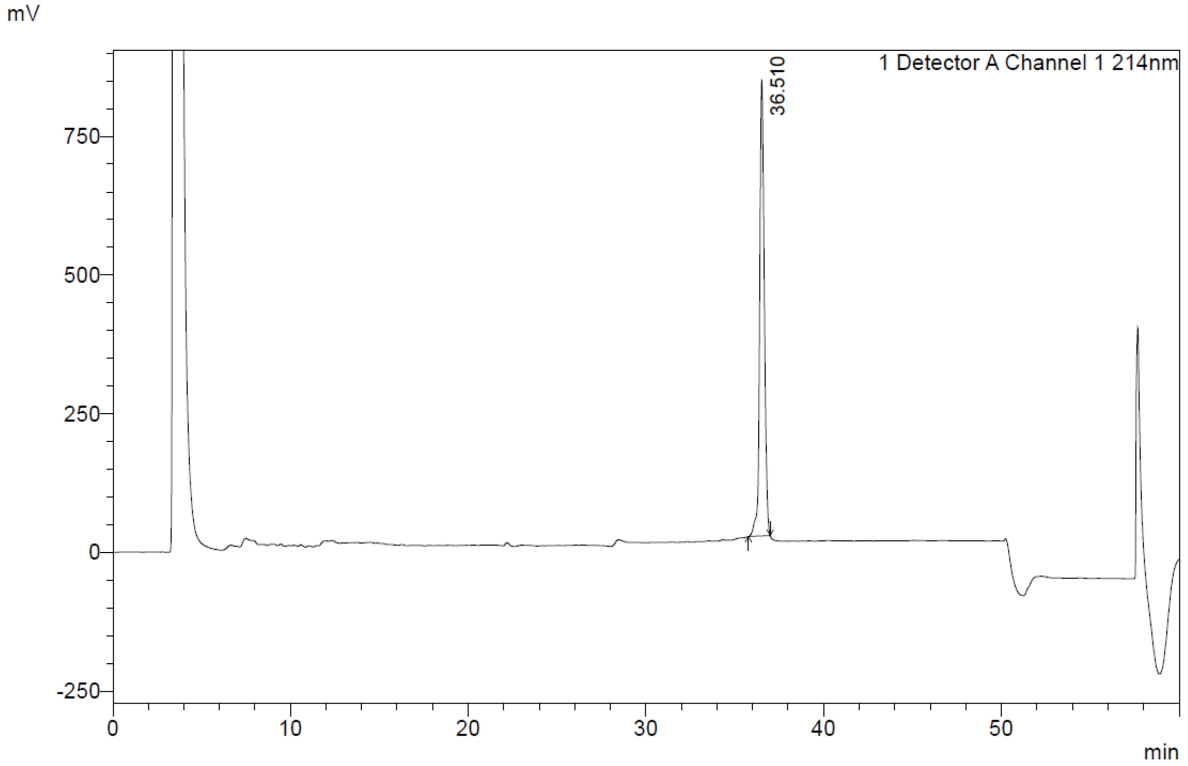


**Ser9-Laterocidine (4)**

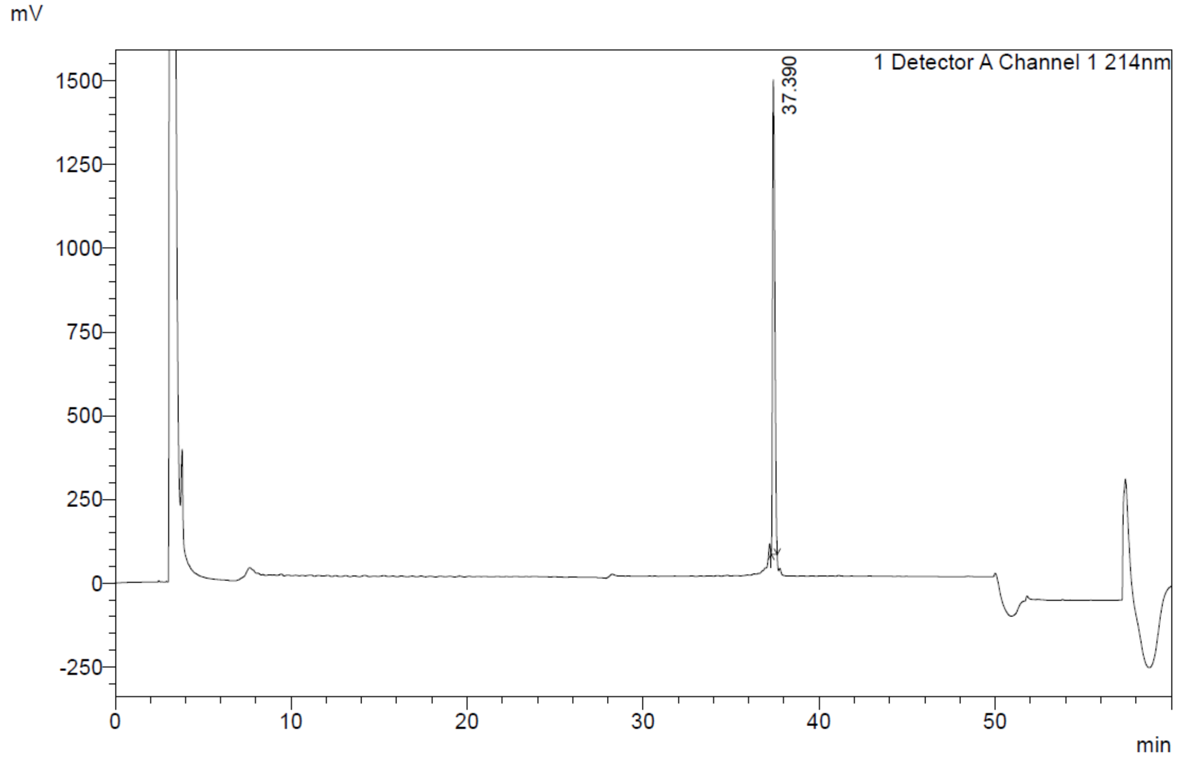

**Dap9-Laterocidine (6)**

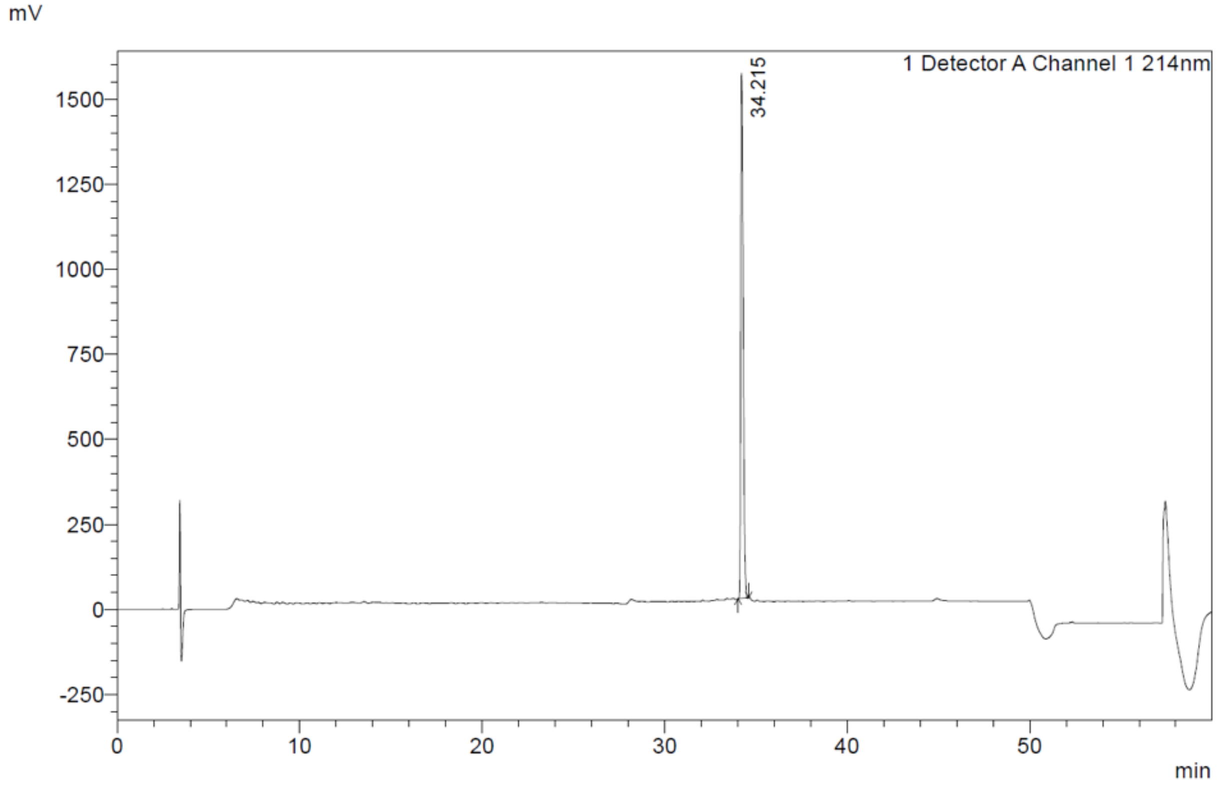


**MeDap9-Laterocidine (8)**

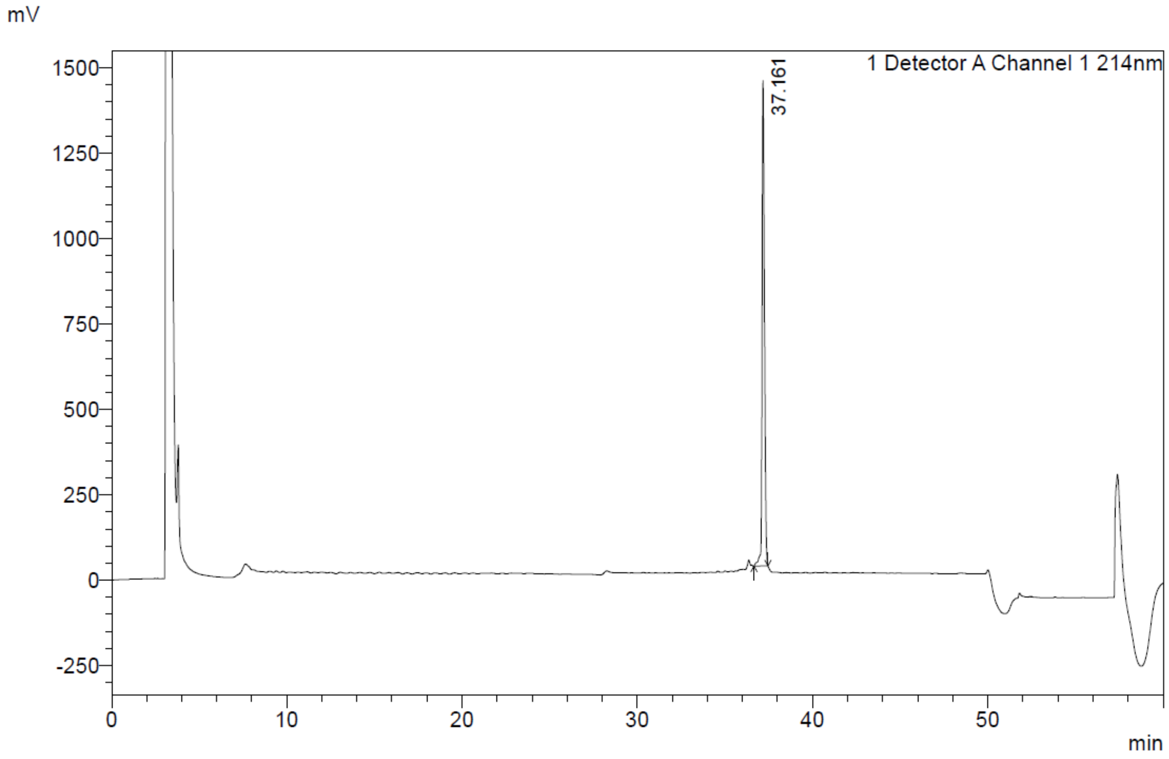

# *NMR characterization of compounds*

## Synthetic brevicidine (1)

| Residue | -NH | Hα | Hβ | Hγ | Hδ | Hε |
|---|---|---|---|---|---|---|
| D-Asn1 | 8.01 (1H, d, *J* = 7.8 Hz) | 4.54 (1H, m) | 2.53 (1H, under DMSO peak) & 2.35 (1H, dd, *J* = 15.7 & 6.8 Hz) | | | |
| D-Tyr2 | 7.98 (1H, d, *J* = 7.3 Hz) | 4.27 (1H, m) | 2.78 (1H, m) & 2.58 (1H, m) | Aromatic: 6.85 (2H, d, J = 8.6 Hz) & 6.55 (2H, d, *J* = 8.6 Hz) | | |
| D-Trp3 | 8.11 (1H, m) | 4.48 (1H, m) | 3.17 (1H, dd, *J* = 14.6 & 4.3 Hz) & 3.02 (1H, dd, *J* = 14.8 & 9.9 Hz) | Indole: 10.89 (2H, br d, *J* = 6.5 Hz), 7.57 (2H, app dd, *J* = 8.2 & 2.2 Hz), 7.32 (2H, app dd, *J* = 14.2 & 8.2 Hz), 7.16 (1H, d, *J* = 2.2 Hz), 7.05 (2H, m) & 6.97 (3H, m) | | |
| D-Orn4 | 8.07 (1H, d, *J* = 8.6 Hz) | 4.37 (1H, m) | 1.73 (1H, m) & 1.53 (1H, m) | 1.53 (2H, m) | 2.78 (2H, m) | 7.76 (2H, br) |
| Orn5 | 7.92 (1H, d, *J* = 8.2 Hz) | 4.37 (1H, m) | 1.73 (1H, m) & 1.53 (1H, m) | 1.53 (2H, m) | 2.78 (2H, m) | 7.76 (2H, br) |
| Gly6 | 8.23 (1H, t, *J* = 5.4 Hz) | 3.82 (1H, dd, *J* = 16.6 & 6.1 Hz) & 3.73 (1H, dd, *J* = 18.4 & 7.8 Hz) | | | | |
| D-Orn7 | 8.11 (1H, m) | 4.37 (1H, m) | 1.40 (1H, m) & 1.29 (1H, m) | 1.29 (2H, m) | 2.58 (2H, m) | 7.68 (2H, br) |
| Trp8 | 8.19 (1H, m) | 4.72 (1H, app q, *J* = 7.8 Hz) | 3.08 (1H, dd, *J* = 14.6 & 5.6 Hz) & 2.96 (1H, dd, *J* = 14.4 & 8.6 Hz) | Indole: 10.89 (2H, app d, *J* = 6.5 Hz), 7.57 (2H, app dd, *J* = 8.2 & 2.2 Hz), 7.32 (2H, app dd, *J* = 14.2 & 8.2 Hz), 7.10 (1H, d, *J* = 2.6 Hz), 7.05 (2H, app q, *J* = 7.8 Hz) & 6.97 (3H, m) | | |
| Thr9 | 8.19 (1H, m) | 4.54 (1H, m) | 4.88 (1H, m) | 1.08 (3H, d, *J* = 6.5 Hz) | | |
| Ile10 | 8.54 (1H, d, *J* = 9.6 Hz) | 4.12 (1H, t, J = 10.8 Hz) | 1.84 (1H, br m) | 0.84 (3H, m), 1.51 (1H, m) & 1.06 (1H, m) | 0.84 (3H, m) | |
| Gly11 | 8.44 (1H, t, *J* = 6.3 Hz) | 3.89 (1H, dd, *J* = 15.5 & 6.5 Hz) & 3.55 (under $H_2O$) | | | | |
| Ser12 | 7.62 (1H, d, *J* = 9.1 Hz) | 4.43 (1H, dt, *J* = 9.0 & 4.7 Hz) | 3.76 (1H, dd, *J* = 11.2 & 4.7 Hz) & 3.60 (1H, dd, *J* = 10.8 & 4.7 Hz) | | | |
| Lipid | 2.06 (2H, m, O=CC**H₂**-), 1.53 (2H, O=CCH₂C**H₂**-), 1.29 (5H, -C**H₂**C**H**(CH₃)C**H₂**CH₃), 1.07 (2H, -C**H₂**CH₃) & 0.81 (6H, -CH(C**H₃**)CH₂C**H₃**) | | | | | |

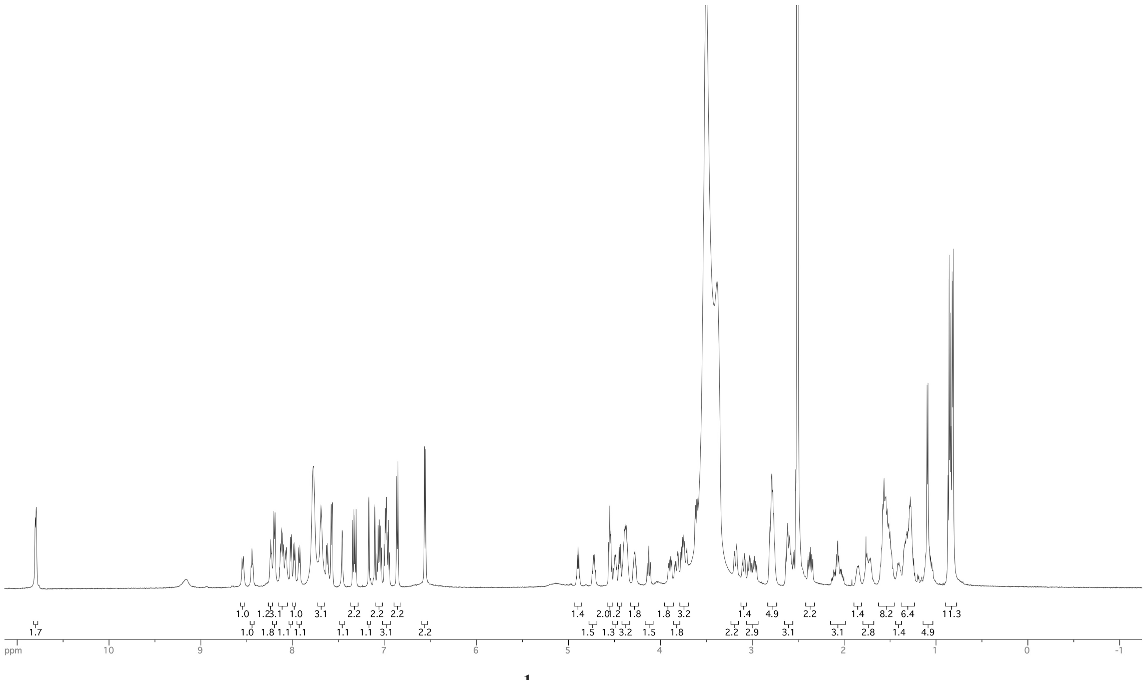


**$^1$H NMR**

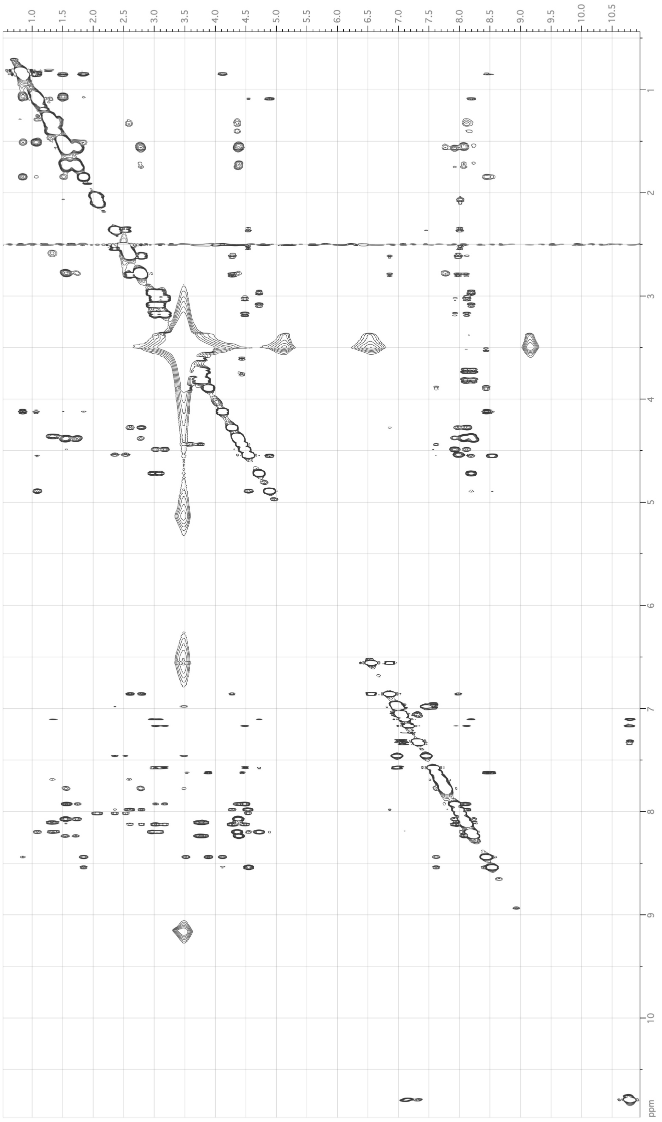

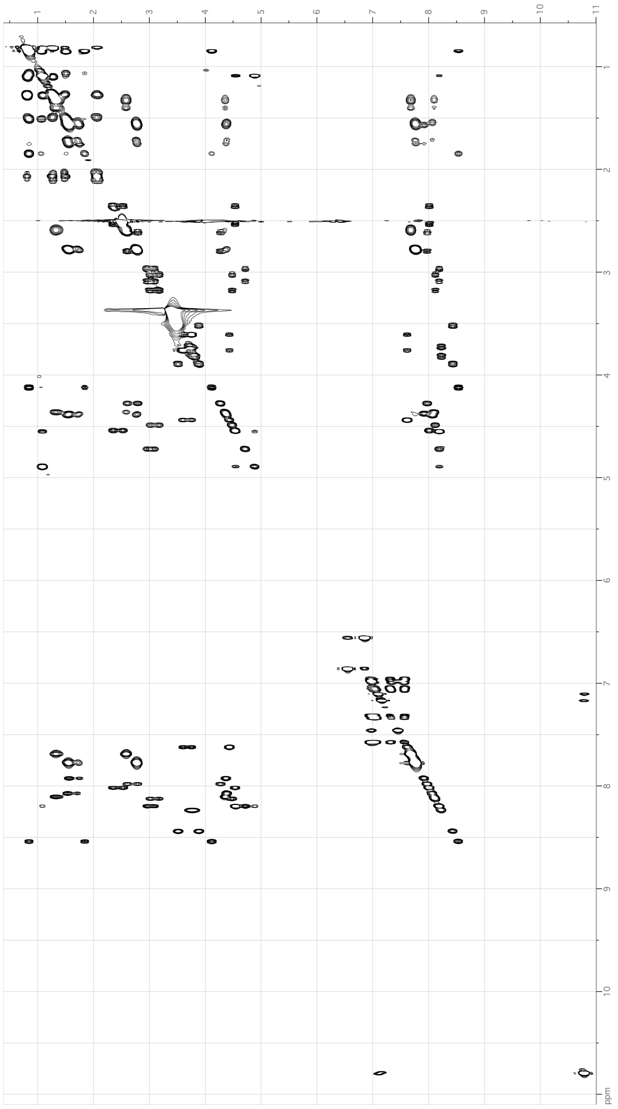

**NOESY** **TOCSY**

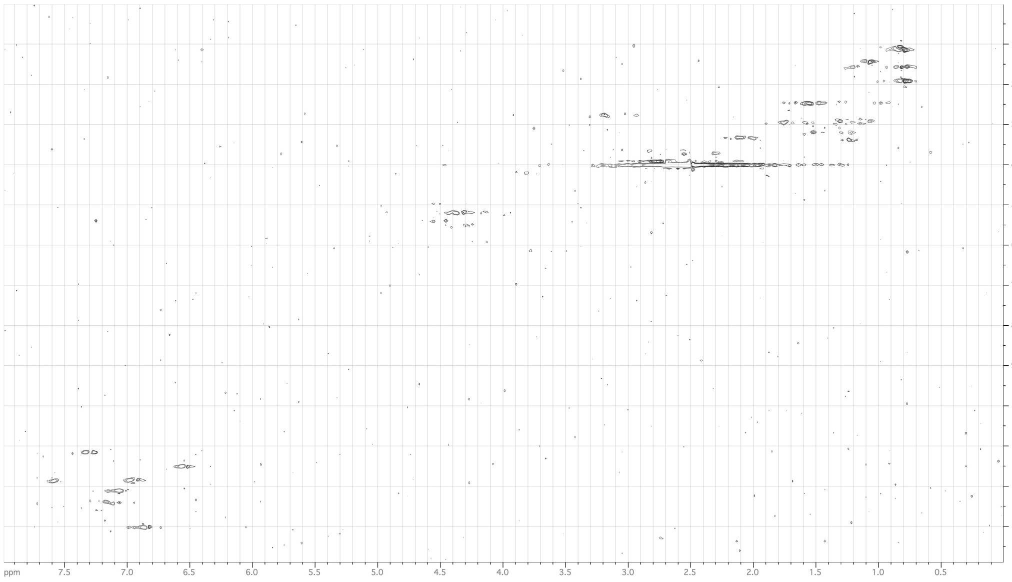

**HSQC**

## *ent*-Brevicidine (*ent*-1)

| Residue | -NH | Hα | Hβ | Hγ | Hδ | Hε |
|---|---|---|---|---|---|---|
| Asn1 | 8.02 (1H, d, *J* = 7.9 Hz) | 4.54 (1H, m) | 2.54 (1H, m) & 2.35 (1H, dd, *J* = 15.2 & 7.0 Hz) | | | |
| Tyr2 | 7.98 (1H, d, *J* = 7.5 Hz) | 4.49 (1H, m) | 2.79 (1H, m) & 2.62 (1H, m) | 6.86 (2H, d, *J* = 8.5 Hz), 6.56 (2H, d, *J* = 8.5 Hz) | | |
| Trp3 | 8.13 (1H, d, *J* = 8.2 Hz) | 4.27 (1H, br) | 3.17 (1H, dd, *J* = 14.6 & 3.4 Hz), 3.02 (1H, dd, *J* = 15.1 & 10.1 Hz) | Indole: 10.80 (2H, app d, *J* = 12.2 Hz), 7.57 (2H, app dd, *J* = 7.8 & 2.2 Hz), 7.32 (2H, app dd, *J* = 12.7 & 8.1 Hz), 7.17 (1H, d, *J* = 2.1 Hz), 7.05 (2H, m), 6.97 (2H, m) | | |
| Orn4 | 8.11 (1H, m) | 4.39 (1H, m) | 1.72 (1H, m) & 1.56 (1H, m) | 1.56 (2H, m) | 2.78 (2H, m) | 7.82 (2H, br m) |
| D-Orn5 | 7.92 (1H, d, *J* = 8.1 Hz) | 4.38 (1H, m) | 1.76 (1H, m) & 1.56 (1H, m) | 1.56 (2H, m) | 2.78 (2H, m) | 7.82 (2H, br m) |
| D-Gly6 | 8.25 (1H, t, *J* = 5.4 Hz) | 3.81 (1H, dd, *J* = 17.0 & 6.6 Hz), 3.73 (m) | | | | |
| Orn7 | 8.09 (1H, m) | 4.36 (1H, m) | 1.41 (1H, br m) & 1.32 (1H, m) | 1.32 (2H, m) | 2.59 (2H, br m) | 7.72 (2H, br) |
| D-Trp8 | 8.20 (1H, d, *J* = 8.6 Hz) | 4,71 (1H, app q, *J* = 8.0 Hz) | 3.09 (1H, dd, *J* = 13.8 & 4.9 Hz), 2.96 (1H, dd, *J* = 15.1 & 8.3 Hz) | Indole: 10.80 (2H, app d, *J* = 12.2 Hz), 7.57 (2H, app dd, *J* = 7.8 & 2.2 Hz), 7.32 (2H, app dd, *J* = 12.7 & 8.1 Hz), 7.11 (1H. d, *J* = 2.3 Hz), 7.05 (2H, m), 6.97 (2H, m) | | |
| D-Thr9 | 8.19 (1H, d, *J* = 9.0 Hz) | 4.55 (1H, m) | 4.89 (1H, m) | 1.09 (3H, d, *J* = 6.4 Hz) | | |
| D-Ile10 | 8.58 (1H, d, *J* = 9.7 Hz) | 4.12 (1H, app t, *J* = 10.5 Hz) | 1.86 (1H, br m) | 0.84 (3H, m), 1.51 (1H, m), 1.07 (1H, m) | 0.84 (3H, m) | |
| D-Gly11 | 8.45 (1H, t, *J* = 6.3 Hz) | 3.87 (1H, dd, *J* = 14.8 & 5.6 Hz), 3.54 (1H, dd, *J* = 15.2 & 6.1 Hz) | | | | |
| D-Ser12 | 7.68 (1H, d, *J* = 8.8 Hz) | 4.44 (1H, dt, *J* = 8.9 & 4.4 Hz) | 3.78 (1H, m) & 3.62 (1H, br m) | | | |
| Lipid | 2.06 (2H, m, O=CC$\mathbf{H_2}$), 1.50 (2H, O=CCH$_2$C$\mathbf{H_2}$), 1.27 (5H, -$\mathbf{CH_2CH}$(CH)$_3$$\mathbf{CH_2}$CH$_3$), 1.09 (2H, -$\mathbf{CH_2}$CH$_3$), 0.82 (6H, -CH(C$\mathbf{H_3}$)CH$_2$C$\mathbf{H_3}$) | | | | | |

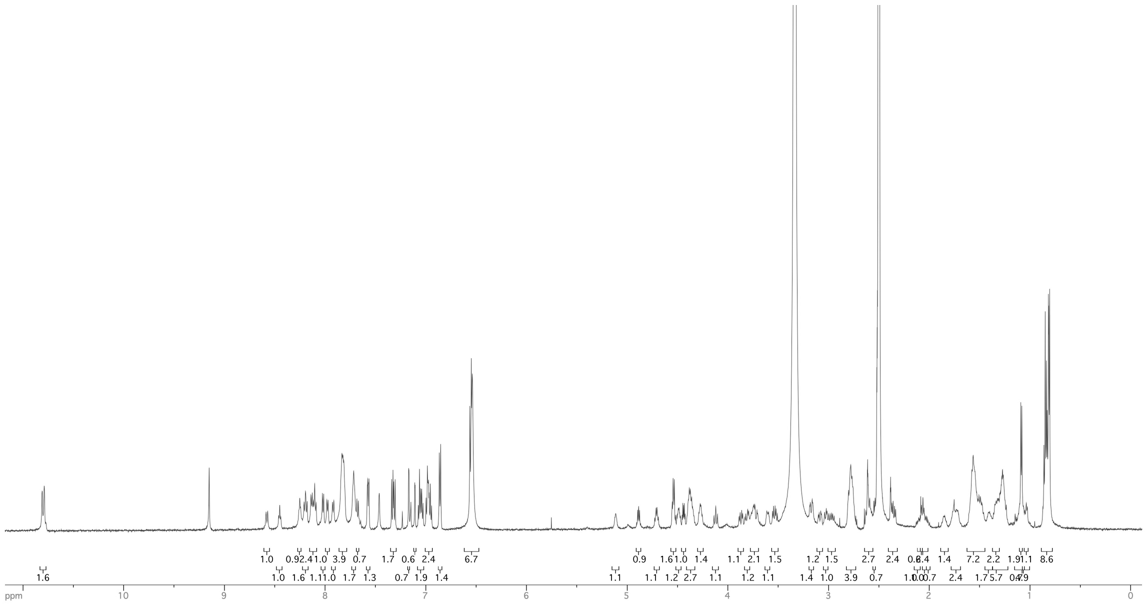


**$^1$H NMR**

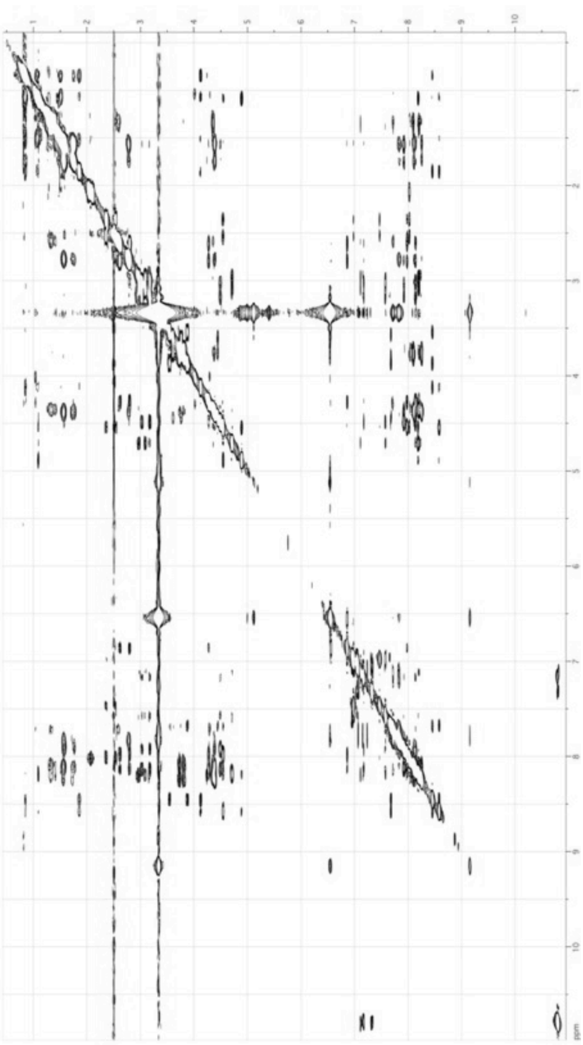

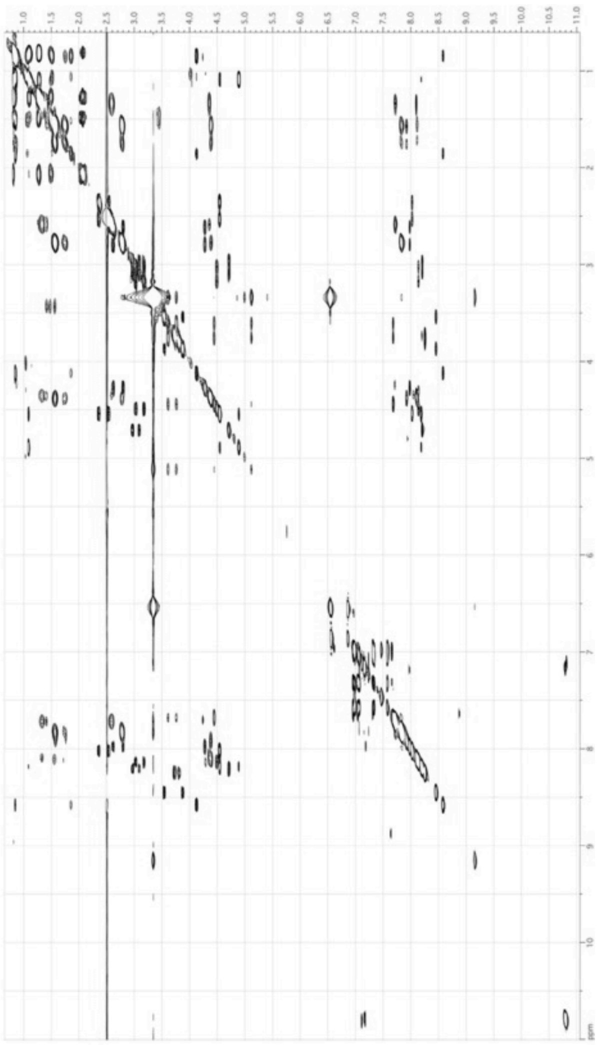

**NOESY** **TOCSY**

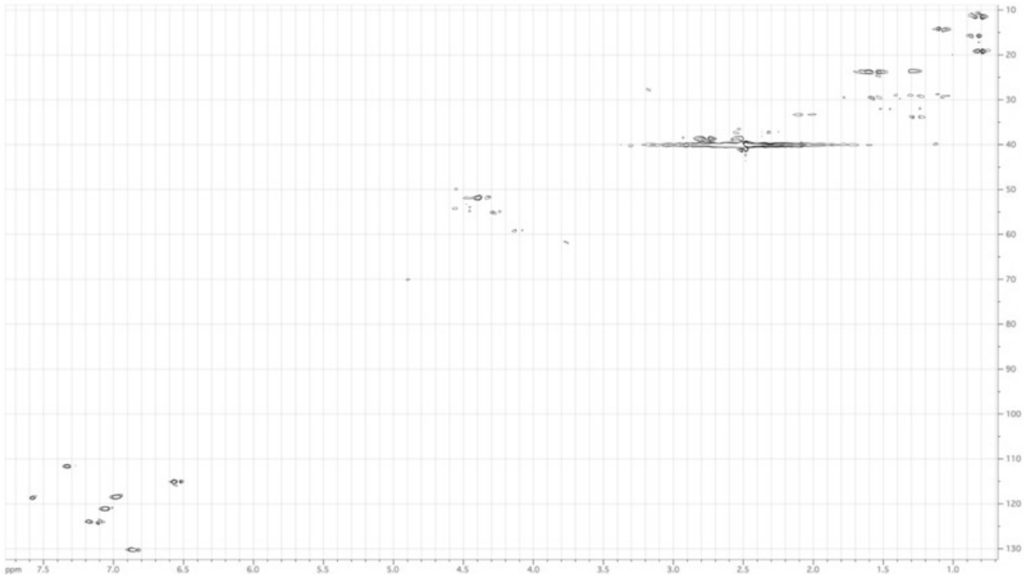

**HSQC**

# Ser9-Brevicidine (3)

| Residue | -NH | Hα | Hβ | Hγ | Hδ | Hε |
|---|---|---|---|---|---|---|
| D-Asn1 | 8.02 (1H, d, *J* = 7.6 Hz) | 4.54 (1H, app q, *J* = 7.1 Hz) | 2.53 (1H, under DMSO peak) & 2.35 (1H, dd, *J* = 15.3 & 6.7 Hz) | | | |
| D-Tyr2 | 7.98 (1H, d, *J* = 7.2 Hz) | 4.27 (1H, m) | 2.79 (1H, m) & 2.62 (1H, m) | Aromatic: 6.86 (2H, d, J = 8.2 Hz) & 6.55 (2, d, *J* = 8.2 Hz) | | |
| D-Trp3 | 8.13 (1H, d, *J* = 7.2 Hz) | 4.49 (1H, br m) | 3.17 (1H, br m) & 3.02 (1H, dd, *J* = 14.4 & 9.8 Hz) | Indole: 10.80 (2H, s), 7.58 (2H, app dd, *J* = 18.9 & 7.8 Hz), 7.32 (2H, app dd, *J* = 14.9 & 8.1 Hz), 7.15 (1H, m), 7.05 (2H, m) & 6.97 (3H, m) | | |
| D-Orn4 | 8.08 (1H, br d, *J* = 7.8 Hz) | 4.38 (1H, m) | 1.72 (1H, m) & 1.55 (1H, m) | 1.55 (2H, m) | 2.78 (6H, m) | 7.78 (2H, br) |
| Orn5 | 7.93 (1H, d, *J* = 8.1 Hz) | 4.37 (1H, m) | 1.72 (1H, m) & 1.55 (1H, m) | 1.55 (2H, m) | 2.78 (6H, m) | 7.78 (2H, br) |
| Gly6 | 8.22 (1H, m) | 3.81 (1H, m) & 3.73 (1H, m) | | | | |
| D-Orn7 | 8.05 (1H, d, *J* = 8.3 Hz) | 4.34 (1H, m) | 1.38 (1H, m) & 1.33 (1H, m) | 1.33 (2H, m) | 2.59 (2H, m) | 7.72 (2H, br) |
| Trp8 | 8.19 (1H, d, *J* = 7.9 Hz) | 4.64 (1H, m) | 3.08 (1H, dd, *J* = 14.2 & & 2.91 (1H, dd, *J* = 14.2 & 8.8 Hz) | Indole: 10.80 (2H, s), 7.58 (2H, app dd, *J* = 18.9 & 7.8 Hz), 7.32 (2H, app dd, *J* = 14.9 & 8.1 Hz), 7.10 (1H, m), 7.05 (2H, m) & 6.97 (3H, m) | | |
| Ser9 | 8.38 (1H, d, *J* = 7.2 Hz) | 4.69 (1H, m) | 4.20 (1H, dd, *J* = 9.8 & 5.1 Hz) & 3.84 (1H, m) | | | |
| Ile10 | 8.71 (1H, d, *J* = 9.7 Hz) | 4.09 (1H, app t, J = 10.1 Hz) | 1.83 (1H, br m) | 0.84 (3H, m), 1.54 (1H, m) & 1.11 (1H, m) | 0.84 (3H, m) | |
| Gly11 | 8.53 (1H, t, *J* = 6.3 Hz) | 3.94 (1H, dd, *j* = 15.1 & 6.4 Hz) & 3.47 (1H, dd, *J* = 15.0 & 6.3 Hz) | | | | |
| Ser12 | 7.46 (1H, br) | 4.43 (1H, m) | 3.71 (1H, m) & 3.57 (1H, m) | | | |
| Lipid | 2.06 (2H, m, O=CC**H$_2$**-), 1.50 (2H, O=CCH$_2$C**H$_2$**-), 1.27 (5H, -**CH$_2$CH(**CH$_3$)**CH$_2$**CH$_3$), 1.10 (2H, -C**H$_2$**CH$_3$) & 0.81 (6H, -CH(C**H$_3$**)CH$_2$C**H$_3$**) | | | | | |

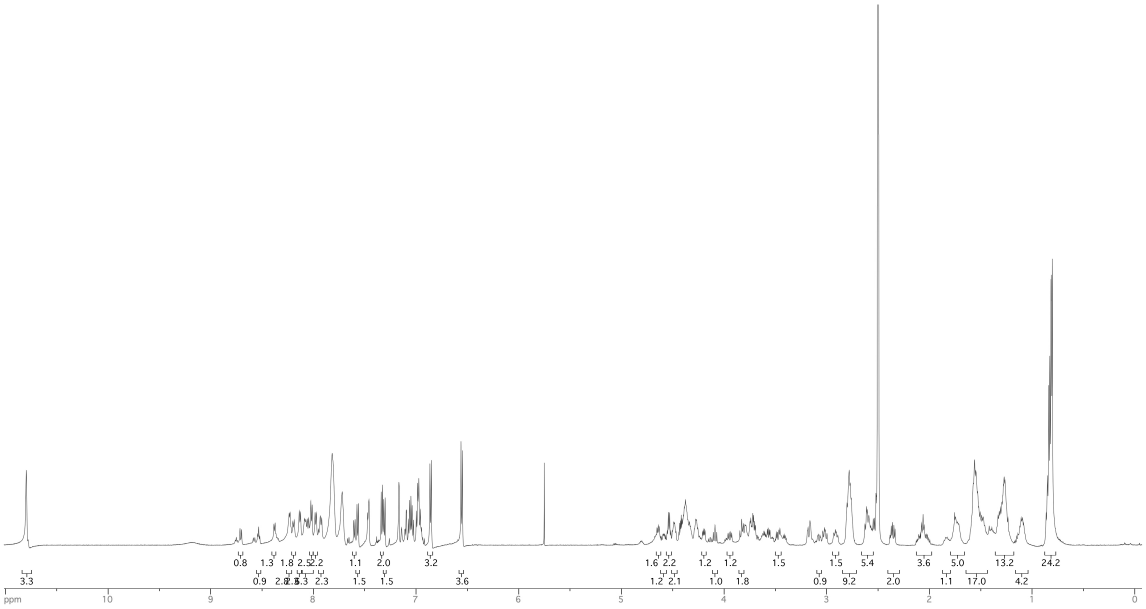


**$^1$H NMR**

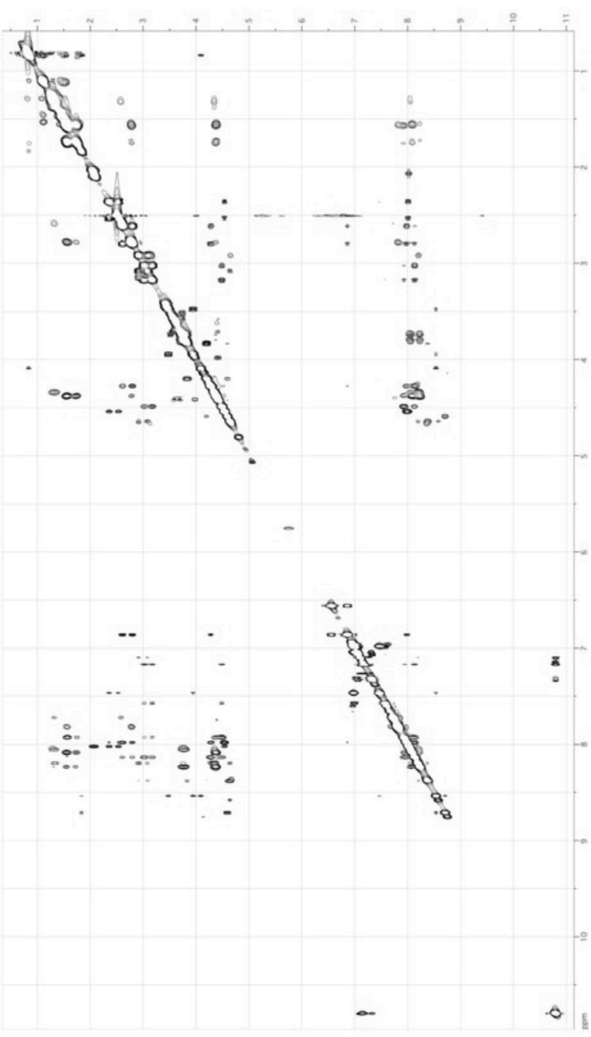

**NOESY**

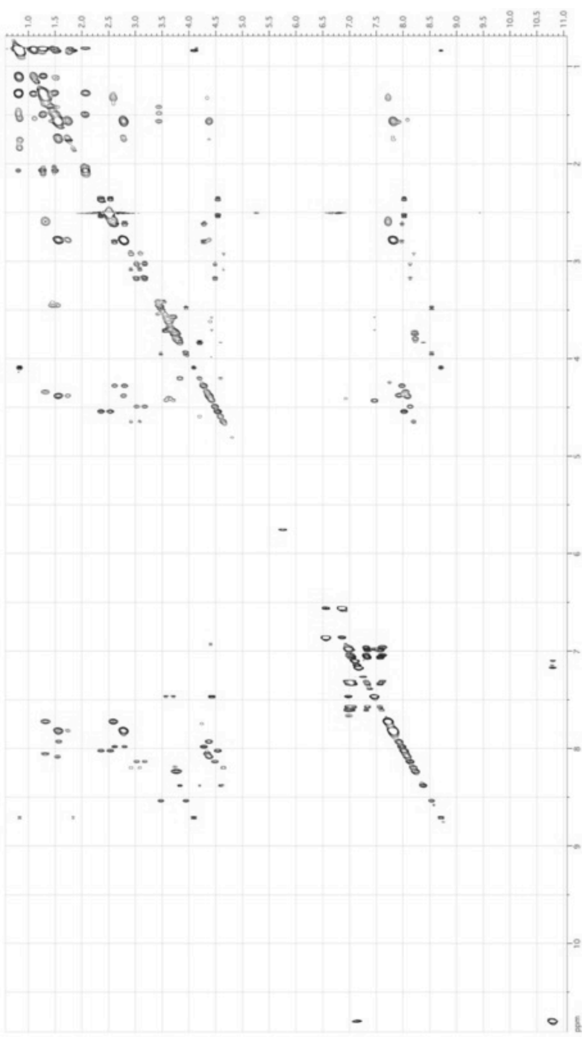

**TOCSY**

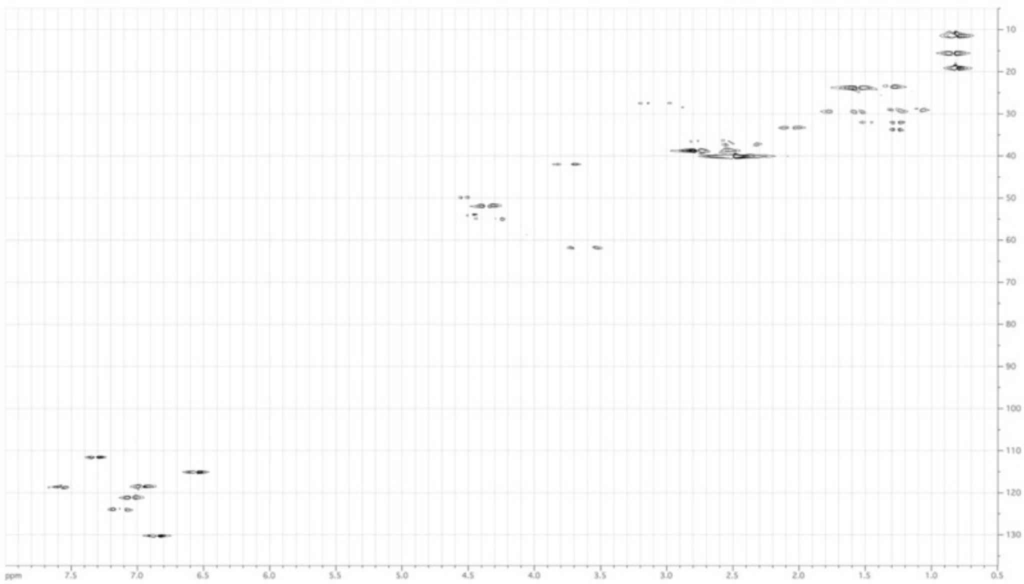

**HSQC**

## Dap9-Brevicidine (5)

| Residue | -NH | H$\alpha$ | H$\beta$ | H$\gamma$ | H$\delta$ | H$\varepsilon$ |
|---|---|---|---|---|---|---|
| D-Asn1 | 8.01 (1H, d, *J* = 7.8 Hz) | 4.53 (1H, q, *J* = 7.2 Hz) | 2.54 (under solvent) & 2.35 (1H, dd, *J* = 15.5 & 7.1 Hz) | | | |
| D-Tyr2 | 7.98 (1H, d, *J* = 7.3 Hz) | 4.28 (1H, m) | 2.77 (1H, m) & 2.61 (1H, m) | Aromatic: 6.85 (2H, d, *J* = 8.5 Hz), 6.55 (2H, d, *J* = 8.4 Hz) | | |
| D-Trp3 | 8.11 (1H, d, *J* = 7.3 Hz) | 4.46 (1H, m) | 3.17 (1H, m) & 3.01 (1H, m) | Indole: 10.78 (2H, br m), 7.32 (2H, dd, *J* = 14.9 & 8.0 Hz),7.17 (1H, br d, *J* = 2.0 Hz), 7.06 (3H, m) & 6.98 (3H, m) | | |
| D-Orn4 | 8.05 (1H, d, *J* = 8.3 Hz) | 4.35 (1H, m) | 1.70 (1H, m) & 1.52 (1H, m) | 1.52 (2H, m) | 2.77 (2H, m) | 7.71 (2H, br) |
| Orn5 | 7.92 (1H, d, *J* = 7.6 Hz) | 4.35 (1H, m) | 1.74 (1H, m) & 1.54 (1H, m) | 1.55 (2H, m) | 2.77 (2H, m) | 7.69 (2H, br) |
| Gly6 | 8.22 (1H, t, *J* = 5.3 Hz) | 3.82 (1H, dd, *J* = 17.1 & 5.3 Hz) & 3.74 (1H, dd, *J* = 16.8 & 4.8 Hz) | | | | |
| D-Orn7 | 8.05 (1H, d, *J* = 8.3 Hz) | 4.35 (1H, m) | 1.28 (2H, m) | 1.28 (2H, m) | 2.58 (2H, m) | 7.61 (2H, m) |
| Trp8 | 8.27 (1H, d, *J* = 8.2 Hz) | 4.60 (1H, m) | 3.05 (1H, m) & 2.88 (1H, m) | Indole: 10.89 (2H, br m), 7.32 (2H, dd, *J* =14.9 & 8.0 Hz), 7.06 (3H, m) & 6.98 (3H, m) | | |
| Dap9 | 8.30 (1H, m) | 4.46 (1H, m) | 3.44 (1H, dd, *J* = 12.7 & 6.0 Hz) & 3.03 (1H, m) | | | |
| Ile10 | 8.29 (1H, m) | 4.05 (1H, app t. *J* = 10.0 Hz) | 1.76 (1H, m) | 1.48 (1H, m), 1.09 (1H, m) & 0.82 (3H, m) | 0.82 (3H, m) | |
| Gly11 | 8.29 (1H, m) | 3.86 (1H, dd, *J* = 14.7 & 6.1 Hz) & 3.53 (1H, dd, *J* = 14.7 & 6.2 Hz) | | | | |
| Ser12 | 7.34 (1H, d, *J* = 8.4 Hz) | 4.20 (1H, m) | 3.57 (1H, m) & 3.34 (1H, m) | | | |
| Lipid | 2.06 (2H, m, O=CC**H$_2$**-), 1.49 (2H, O=CCH$_2$C**H$_2$**-), 1.25 (5H, -C**H$_2$**C**H**(CH$_3$)C**H$_2$**CH$_3$), 1.08 (2H, m, -C**H$_2$**CH$_3$) 0.80 (6H, -CH(C**H$_3$**)CH$_2$C**H$_3$**). | | | | | |

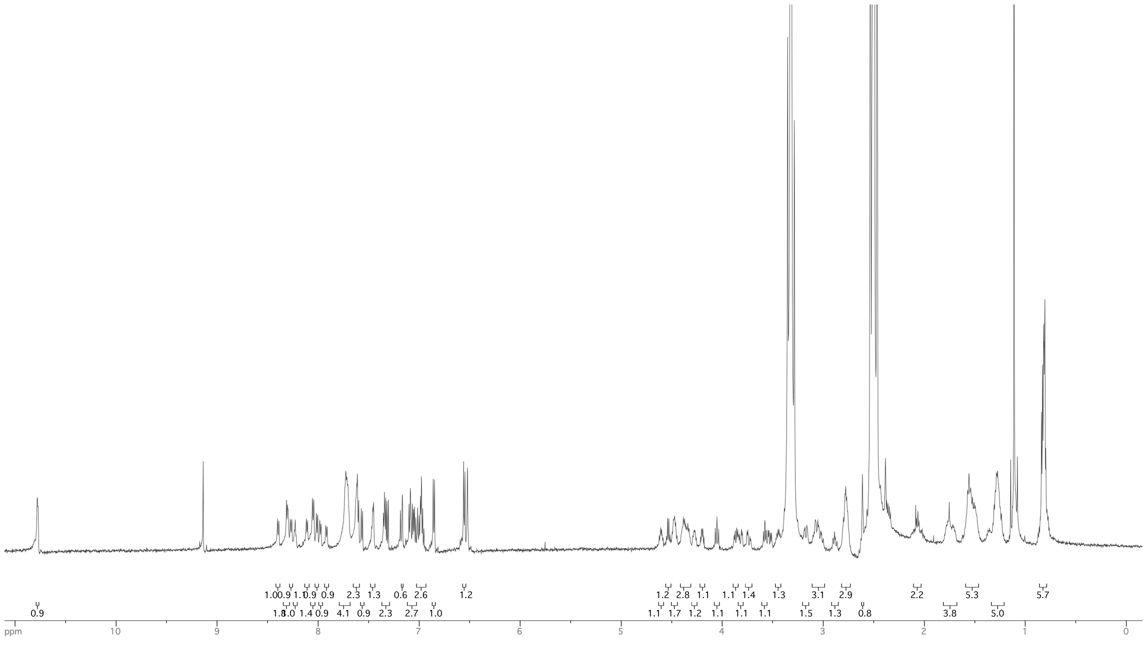

**$^1$H NMR**

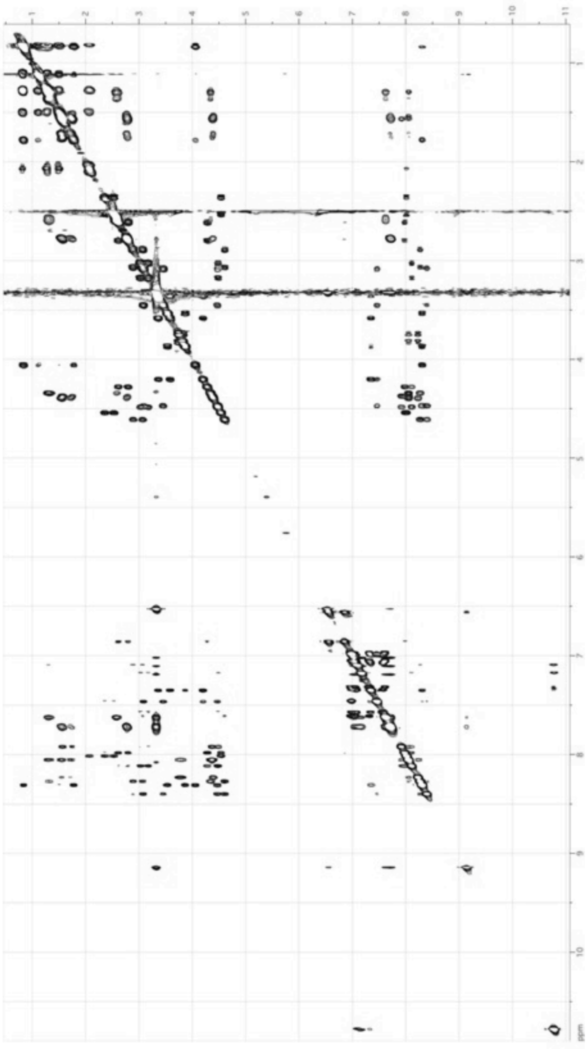

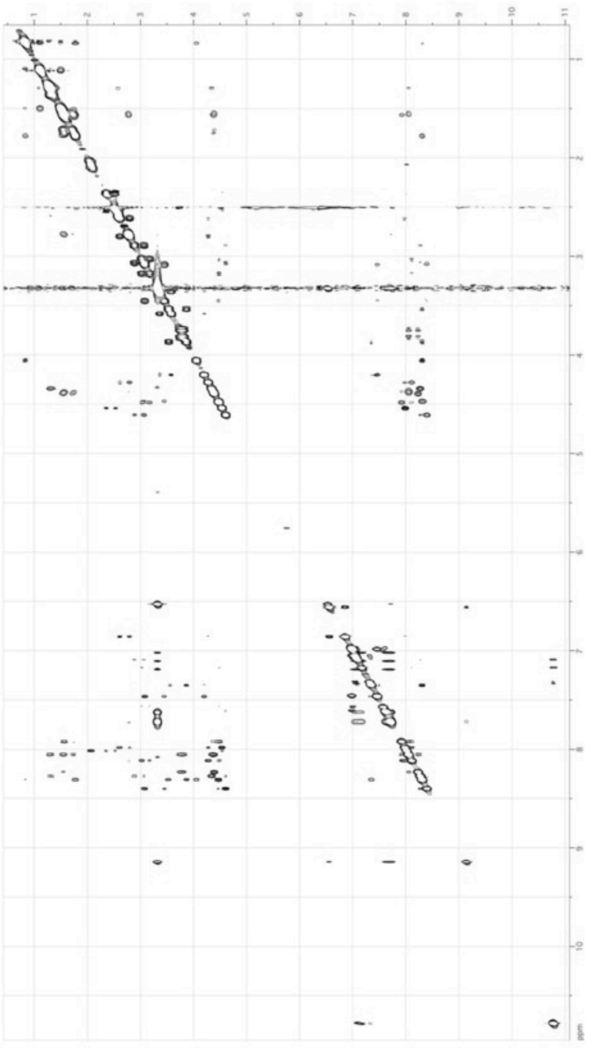

**NOESY** **TOCSY**

## MeDap9-Brevicidine (7)

| Residue | -NH | Hα | Hβ | Hγ | Hδ | Hε |
|---|---|---|---|---|---|---|
| D-Asn1 | 8.03 (1H, d, *J* = 7.8 Hz) | 4.53 (1H, app q, *J* = 7.2 Hz) | 2.52 (Under DMSO peak) & 2.35 (1H, dd, *J* = 15.2 & 7.0 Hz) | | | |
| D-Tyr2 | 7.98 (1H, d, *J* = 7.5 Hz) | 4.27 (1H, m) | 2.79 (1H, m) & 2.62 (1H, m) | Aromatic: 6.86 (2H, d, *J* = 8.4 Hz), 6.56 (2H, d, *J* = 8.5 Hz) | | |
| D-Trp3 | 8.15 (1H, m) | 4.42 (1H, m) | 3.17 (1H, dd, *J* = 14.9 & 4.0 Hz) & 3.02 (1H, dd, *J* = 14.5 & 8.9 Hz) | Indole: 10.81 (2H, d, *J* = 15.8 Hz), 7.57 (2H, d, *J* = 7.8 Hz), 7.32 (2H, app dd, *J* = 11.1 & 8.1 Hz), 7.17 (1H, d, *J* = 2.1 Hz), 7.05 (2H, m) & 6.97 (2H, m). | | |
| D-Orn4 | 8.14 (1H, m) | 4.37 (1H, m) | 1.73 (1H, m) & 1.55 (1H, m) | 1.55 (2H, m) | 2.78 (2H, m) | 7.86 (2H, br m) |
| Orn5 | 7.92 (1H, d, *J* = 8.1 Hz) | 4.38 (1H, m) | 1.74 (1H, m) & 1.58 (1H, m) | 1.58 (2H, m) | 2.78 (2H, m) | 7.86 (2H, br m) |
| Gly6 | 8.27 (1H, t, *J* = 5.6 Hz) | 3.81(1H, dd, *J* = 16.7 & 5.6 Hz) & 3.73 (1H, dd, *J* = 17.1 & 5.2 Hz) | | | | |
| D-Orn7 | 8.09 (1H, d, *J* = 8.4 Hz) | 4.36 (1H, m) | 1.34 (2H, m) | 1.34 (2H, m) | 2.59 (2H, m) | 7.73 (2H, br) |
| Trp8 | 8.24 (1H, d, *J* = 8.2 Hz) | 4.68 (1H, m) | 3.11 (1H, dd, *J* = 15.6 & 4.5 Hz) & 2.95 (1H, dd, *J* = 14.4 & 8.7 Hz) | Indole: 10.81 (2H, d, *J* = 15.8 Hz), 7.57 (2H, d, *J* = 7.8 Hz), 7.32 (2H, app dd, *J* = 11.1 & 8.1 Hz), 7.12 (1H, d, *J* = 2.1 Hz), 7.05 (2H, m) & 6.97 (2H, m). | | |
| MeDpa-9 | 8.14 (1H, m) | 4.41 (1H, dd, *J* = 8.4 & 4.4 Hz) | 4.18 (1H, m) | 1.01 (3H, d, *J* = 6.8 Hz) NH: 6.86 (2H, d, *J* = 8.4 Hz) | | |
| Ile10 | 8.59 (1H, d, *J* = 9.5 Hz) | 4.07 (1H, app t, *J* = 10.2 Hz) | 1.85 (1H, br) | 0.86 (3H, m), 1.54 (1H, m) & 1.07 (1H, m) | 0.86 (3H, m) | |
| Gly11 | 8.65 (1H, t, *J* = 5.7 Hz) | 3.93 (1H, dd, *J* =14.7 & 6.8 Hz) & 3.42 (under $H_2O$) | | | | |
| Ser12 | 7.54 (1H, d, *J* = 8.9 Hz) | 4.20 (1H, m) | 3.66 (1H, m) & 3.52 (1H, m) | | | |
| Lipid | 2.06 (2H, m, O=CC$\mathbf{H_2}$-), 1.50 (2H, O=CCH$_2$C$\mathbf{H_2}$-), 1.26 (5H, -$\mathbf{CH_2CH}$(CH$_3$)$\mathbf{CH_2}$CH$_3$), 1.06 (2H, -$\mathbf{CH_2}$CH$_3$) & 0.81 (6H, -CH($\mathbf{CH_3}$)CH$_2\mathbf{CH_3}$). | | | | | |

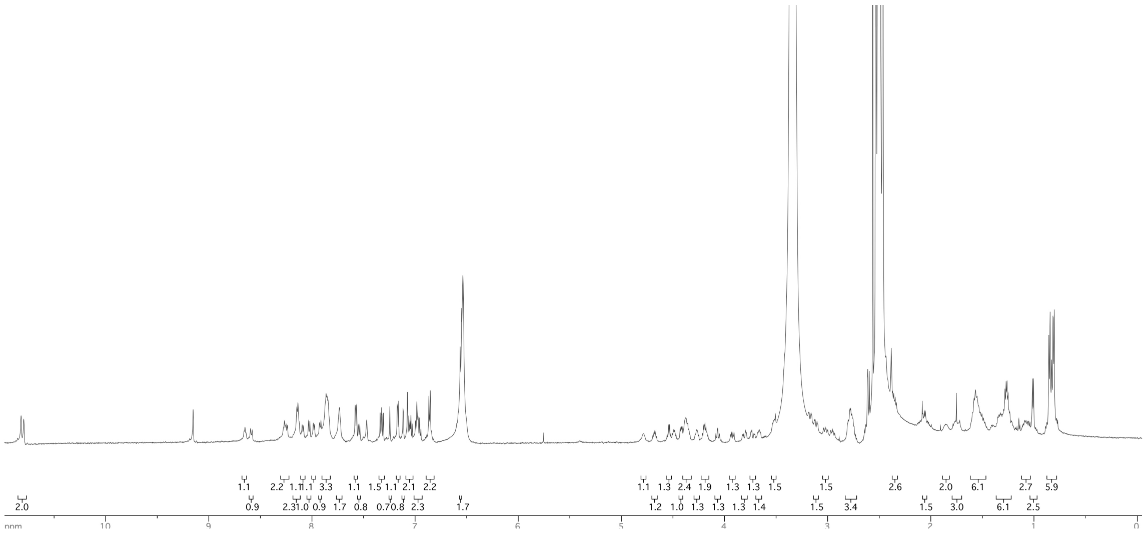

**$^1$H NMR**

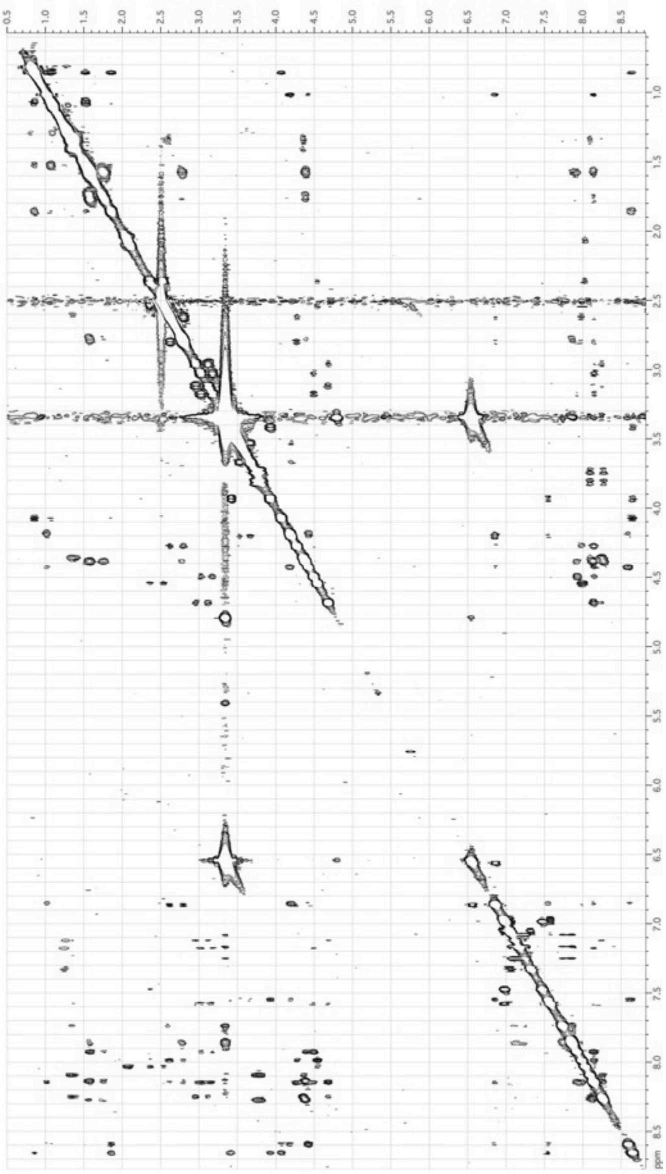

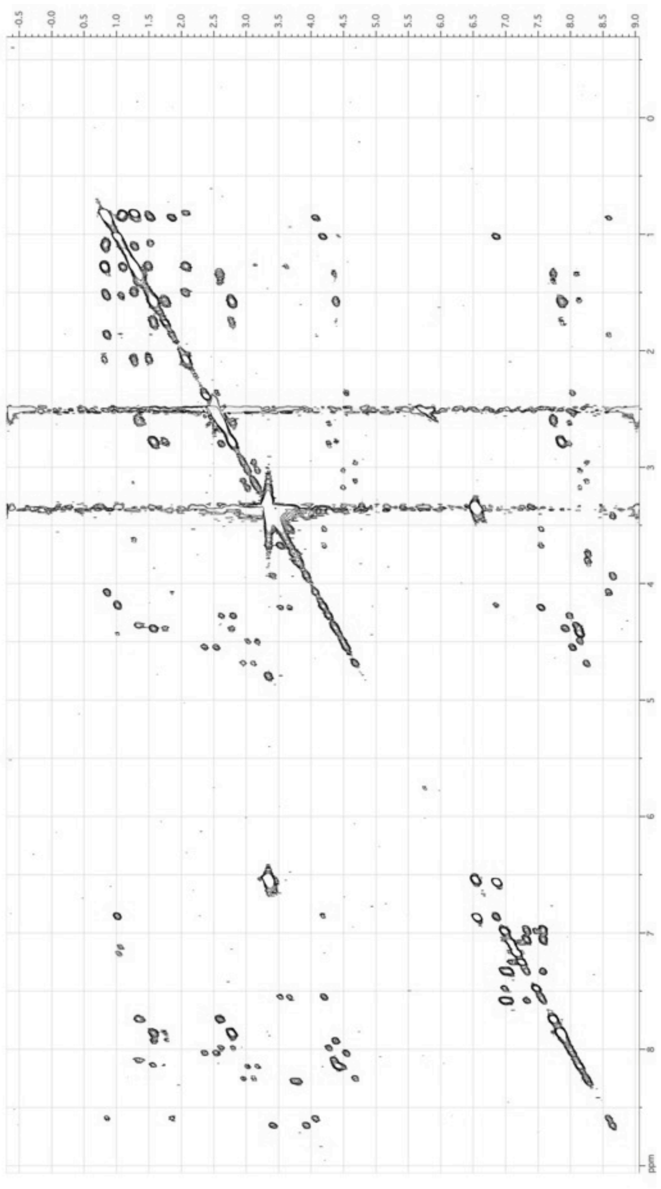

**NOESY** **TOCSY**

## Synthetic laterocidine (2)

| Residue | -NH | Hα | Hβ | Hγ | Hδ | Hε |
|---|---|---|---|---|---|---|
| Ser1 | 7.85 (1H, d, *J* = 7.4 Hz) | 4.26 (1H, dd, *J* = 13.5 & 6.4 Hz) | 3.50 (2H, m) | 5.12 (1H, t, *J* = 5.4 Hz) | | |
| Tyr2 | 8.00 (1H, m) | 4.34 (1H, m) | 2.83 (1H, dd, *J* = 14.3 Hz & 4.4 Hz) & 2.62 (1H, dd, *J* = 14.1 & 9.5 Hz) | Aromatic: 6.89 (2H, d, *J* = 8.5 Hz), 6.56 (2H, d, *J* = 8.5 Hz) | | |
| Trp3 | 8.00 (1H, m) | 4.52 (1H, m) | 3.15 (1H, m) & 2.93 (1H, m) | Indole: 10.79 (1H, s), 7.57 (1H, d, *J* = 7.8 Hz), 7.33 (1H, d, *J* = 8.0 Hz), 7.14 (1H, br s), 7.06 (1H, t, *J* = 8.0 Hz) & 6.98 (1H, t, *J* = 7.4 Hz) | | |
| Orn4 | 8.03 (1H, m) | 4.39 (1H, m) | 1.73 (1H, m) & 1.56 (1H, m) | 1.56 (2H, m) | 2.82 (2H, m) | 7.71 (2H, m) |
| D-Orn5 | 8.09 (1H, d, *J* = 8.3 Hz) | 4.41 (1H, m) | 1.70 (1H, m) & 1.52 (1H, m) | 1.52 (2H, m) | 2.76 (2H, m) | 7.71 (2H, m) |
| D-Gly6 | 8.24 (1H, t, *J* = 5.2 Hz) | 3.85 (1H, dd, *J* = 17.2 & 5.7 Hz) & 3.75 (1H, dd, *J* = 16.8 & 5.0 Hz) | | | | |
| Orn7 | 8.07 (1H, d, *J* = 8.5 Hz) | 4.40 (1H, m) | 1.43 (1H, m) & 1.29 (1H, m) | 1.43 (2H, m) | 2.59 (2H, m) | 7.63 (2H, m) |
| D-Trp8 | 8.32 (1H, d, *J* = 8.1 Hz) | 4.75 (1H, m) | 3.14 (1H, m) & 2.93 (1H, m) | Indole: 10.76 (1H, s), 7.59 (1H, d, *J* = 8.0 Hz), 7.32 (1H, d, *J* = 8.1 Hz), 7.12 (1H, br s), 7.04 (1H, t, *J* = 7.6 Hz) & 6.96 (1H, t, *J* = 7.6 Hz) | | |
| D-Thr9 | 7.92 (1H, d, *J* = 6.4 Hz) | 4.68 (1H, dd, *J* = 8.8 & 3.1 Hz) | 5.17 (1H, m) | 1.09 (3H, d, *J* = 6.4 Hz) | | |
| D-Ile10 | 8.30 (1H, d, *J* = 3.1 Hz) | 3.90 (1H, m) | 1.57 (1H, m) | 1.57 (1H, m), 1.11 (1H, m) & 0.86 (3H, m) | 0.86 (3H, m) | |
| D-Asn11 | 9.40 (1H, d, *J* = 6.7 Hz) | 4.12 (1H, m) | 2.89 (1H, m) & 2.65 (1H, m) | | | |
| D-Gly12 | 8.03 (1H, m) | 4.02 (1H, dd, *J* = 16.9 & 8.3 Hz) & 3.58 (1H, dd, *J* = 17.1 & 4.6 Hz) | | | | |
| D-Gly13 | 7.67 (1H, m) | 4.45 (1H, dd, *J* = 16.6 & 10.0 Hz) & 3.37 (under $H_2O$) | | | | |
| Lipid | 2.10 (2H, m, O=CC$\mathbf{H_2}$-), 1.45 (3H, O=CCH$_2$C$\mathbf{H_2}$- & -CH$_2$C$\mathbf{H}$(CH$_3$)$_2$), 1.20 (4H, -$\mathbf{CH_2CH_2}$CH(CH$_3$)$_2$), 1.11 (2H, -$\mathbf{CH_2}$CH(CH$_3$)$_2$) & 0.83 (6H,-CH$_2$CH($\mathbf{CH_3}$)$_2$) | | | | | |

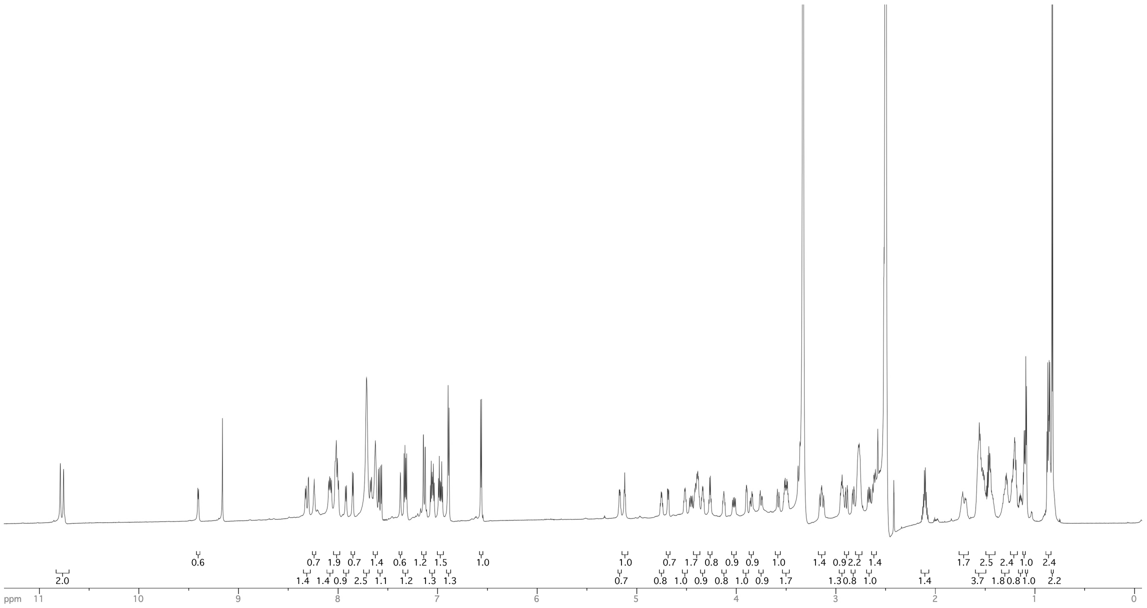


## $^1$H NMR

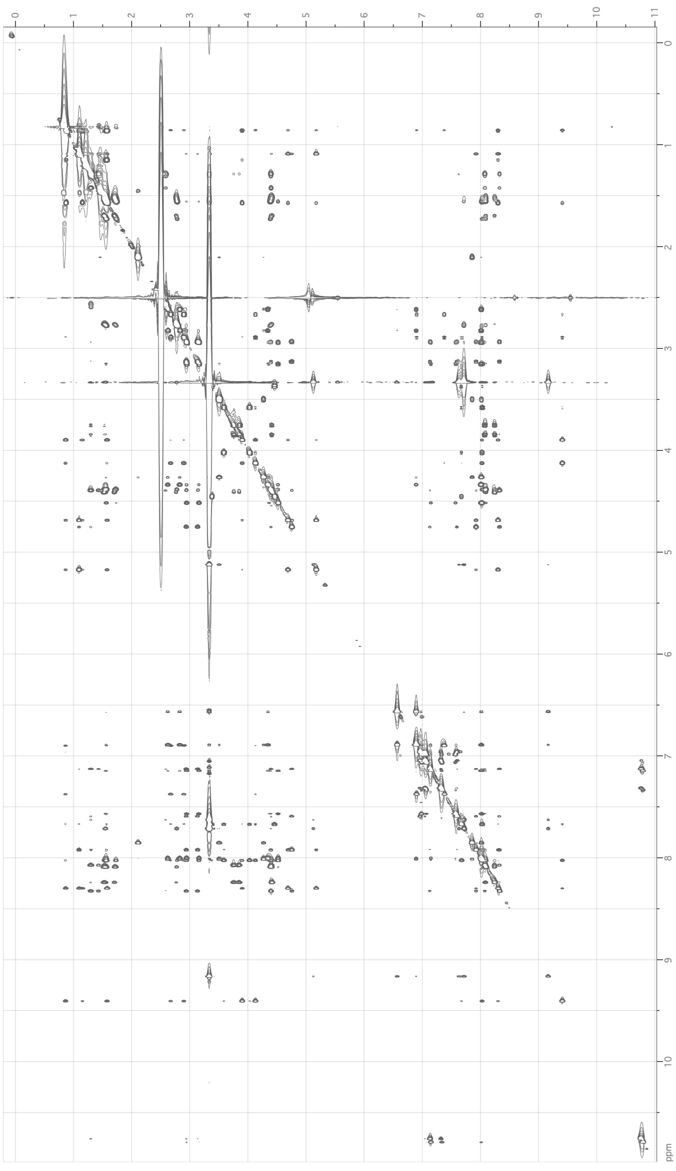

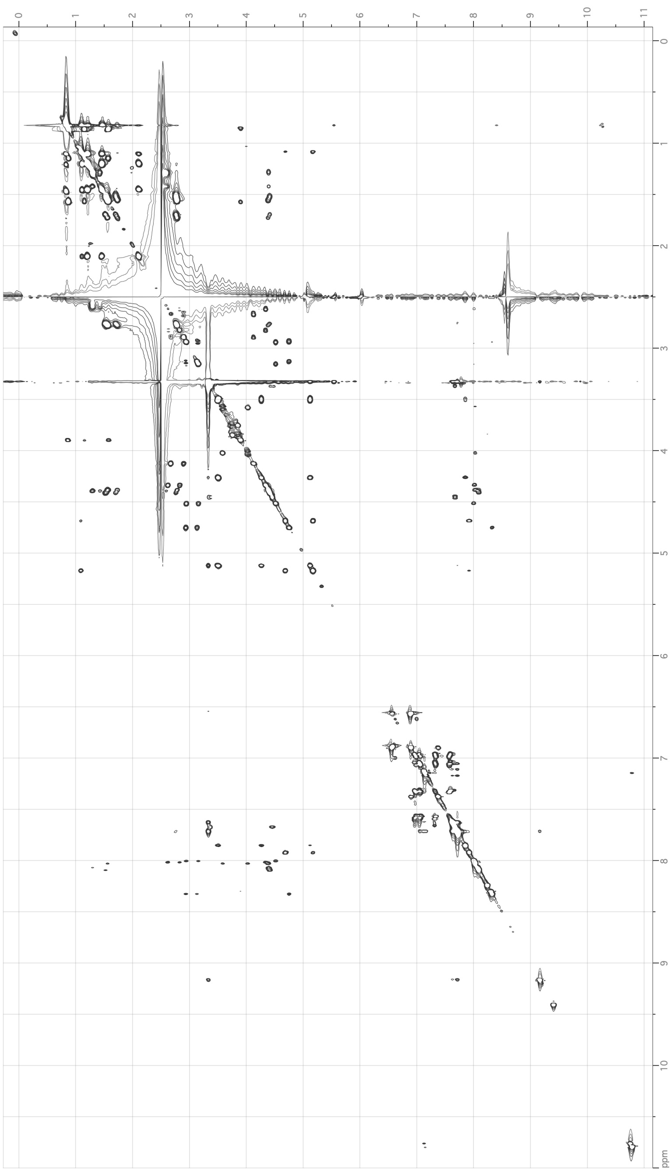

**NOESY** **TOCSY**

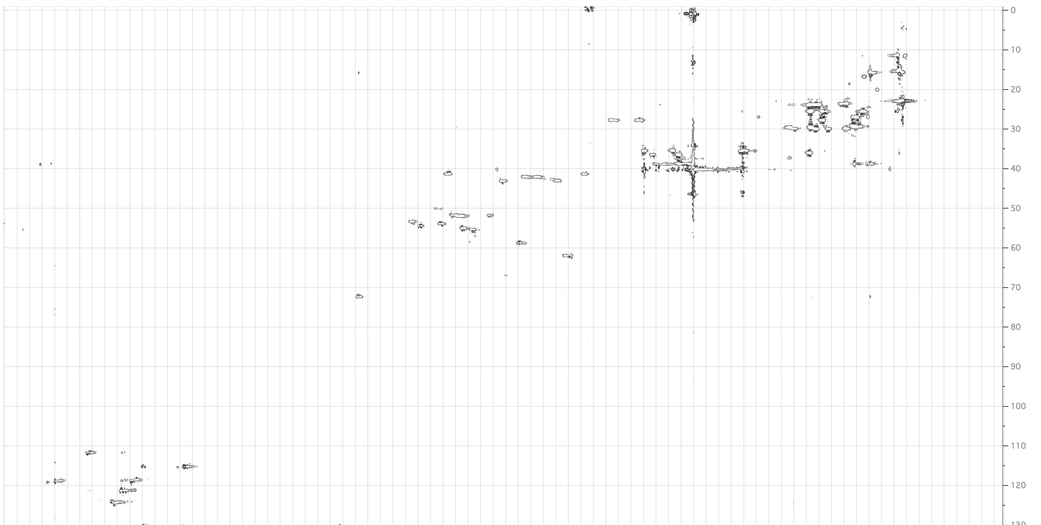

**HSQC**

## *ent*-Laterocidine (*ent*-2)

| Residue | -NH | Hα | Hβ | Hγ | Hδ | Hε |
|---|---|---|---|---|---|---|
| D-Ser1 | 7.85 (1H, d, *J* = 7.4 Hz) | 4.27 (1H, q, *J* = 6.6 Hz) | 3.50 (2H, m) | -OH: 5.13 (1H, t, *J* = 5.3 Hz) | | |
| D-Tyr2 | 8.02 (1H, m) | 4.34 (1H, td, *J* = 8.3, 4.7 Hz) | 2.82 (1H, dd, *J* = 14.1, 3.9 Hz) & 2.62 (1H, dd, *J* = 14.2, 9.8 Hz) | Aromatic: 6.89 (2H, d, *J* = 8.4 Hz) & 6.56 (2H, d, *J* = 8.3 Hz) & 9.17 (1H, s, -OH) | | |
| D-Trp3 | 8.00 (1H, m) | 4.52 (1H, app dd, *J* = 13.3, 8.0 Hz) | 3.15 (1H, dd, *J* = 15.3, 4.2 Hz) & 2.94 (m) | Indole: 10.79 (1H, s), 7.57 (1H, d, *J* = 7.9 Hz), 7.33 (1H, d, *J* = 8.1 Hz), 7.14 (1H, app s), 7.06 (1H, t, *J* = 7.6 Hz) & 6.98 (1H, t, *J* = 7.5 Hz) | | |
| D-Orn4 | 8.02 (1H, m) | 4.38 (1H, m) | 1.72 (1H, m) & 1.55 (1H, m) | 1.55 (2H, m) | 2.78 (2H, m) | 7.73 (2H, br) |
| Orn5 | 8.10 (1H, d, *J* = 8.1 Hz) | 4.41 (1H, m) | 1.70 (1H, m) & 1.52 (1H, m) | 1.51 (2H, m) | 2.76 (2H, m) | 7.73 (2H, br) |
| Gly6 | 8.24 (1H, app t, *J* = 5.3 Hz) | 3.85 (1H, dd, *J* = 16.7 & 5.7 Hz) & 3.75 (1H, dd, *J* = 16.7 & 4.9 Hz) | | | | |
| D-Orn7 | 8.07 (1H, d, *J* = 8.2 Hz) | 4.39 (1H, m) | 1.43 (1H, m) & 1.29 (1H, m) | 1.29 (1H, m) | 2.58 (2H, br s) | 7.65 (2H, m) |
| Trp8 | 8.32 (1H, d, *J* = 7.7 Hz) | 4.75 (1H, app dd, *J* = 13.9, 8.2 Hz) | 3.13 (1H, dd, *J* = 15.9, 5.5 Hz) & 2.93 (1H, m) | Indole: 10.76 (1H, s), 7.59 (1H, d, *J* = 7.9 Hz), 7.32 (1H, d, *J* = 8.0 Hz), 7.13 (1H, app s), 7.04 (1H, t, *J* = 7.4 Hz) & 6.96 (1H, t, *J* = 7.4 Hz) | | |
| Thr9 | 7.93 (1H, d, *J* = 8.6 Hz) | 4.69 (1H, dd, *J* = 8.6, 3.0 Hz) | 5.17 (1H, m) | 1.09 (2H, d, *J* = 6.2 Hz) | | |
| Ile10 | 8.30 (1H, d, *J* = 2.6 Hz) | 3.90 (1H, dd, *J* = 6.6, 3.8 Hz) | 1.58 (1H, m) | 1.58 (1H, m), 1.10 (1H, m) & 0.87 (2H, m) | 0.87 (3H, m) | |
| Asn11 | 9.41 (1H, d, *J* = 6.7 Hz) | 4.12 (1H, m) | 2.89 (1H, dd, *J* = 16.1, 3.5 Hz) & 2.67 (1H, dd, *J* = 16.1, 9.5 Hz) | -$NH_2$: 7.37 (1H, br s) | | |
| Gly12 | 8.03 (1H, m) | 4.02 (1H, dd, *J* = 17.0, 8.2 Hz), 3.58 (1H, dd, *J* = 16.9, 4.3 Hz) | | | | |
| Gly13 | 7.67 (1H, m) | 4.46 (1H, dd, *J* = 16.6, 9.9 7 Hz), 3.38 (under $H_2O$) (1H, s) | | | | |
| Lipid | 2.11 (2H, m, O=CC**H₂**-), 1.48 (1H, -$CH_2$**CH**$(CH_3)_2$), 1.45 (2H, O=CC$H_2$C**H₂**-), 1.20 (2H, -C**H₂**$CH_2CH(CH_3)_2$), 1.19 (2H, br m, O=C$(CH_2)_2$C**H₂**-), 1.10 (2H, -C**H₂**$CH(CH_3)_2$), 0.83 (6H,-$CH_2CH$(C**H₃**)₂) | | | | | |

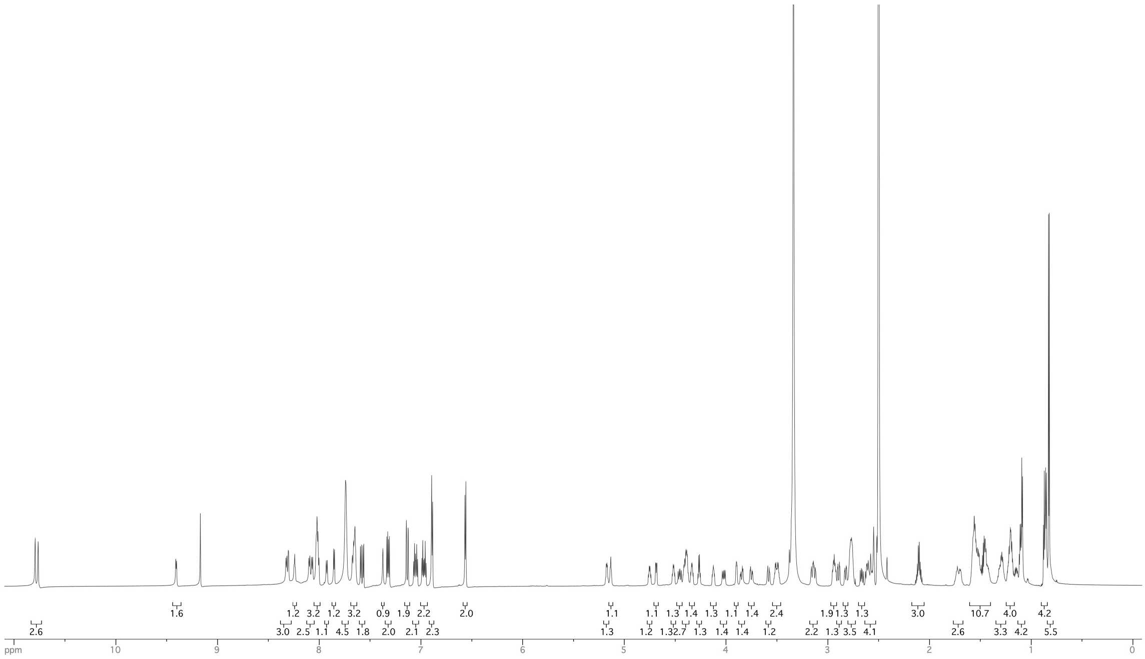


**$^1$H NMR**

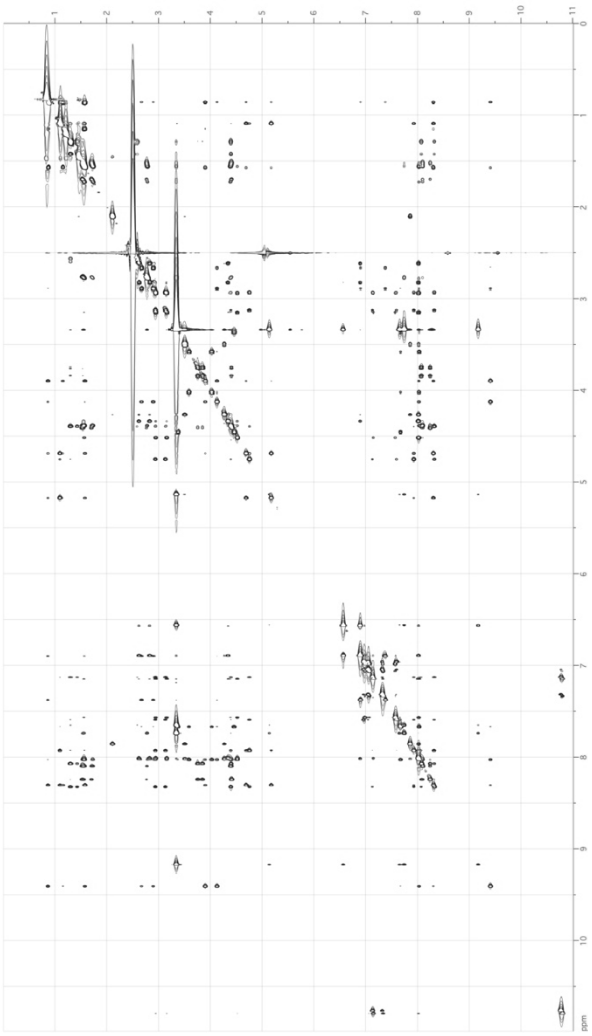

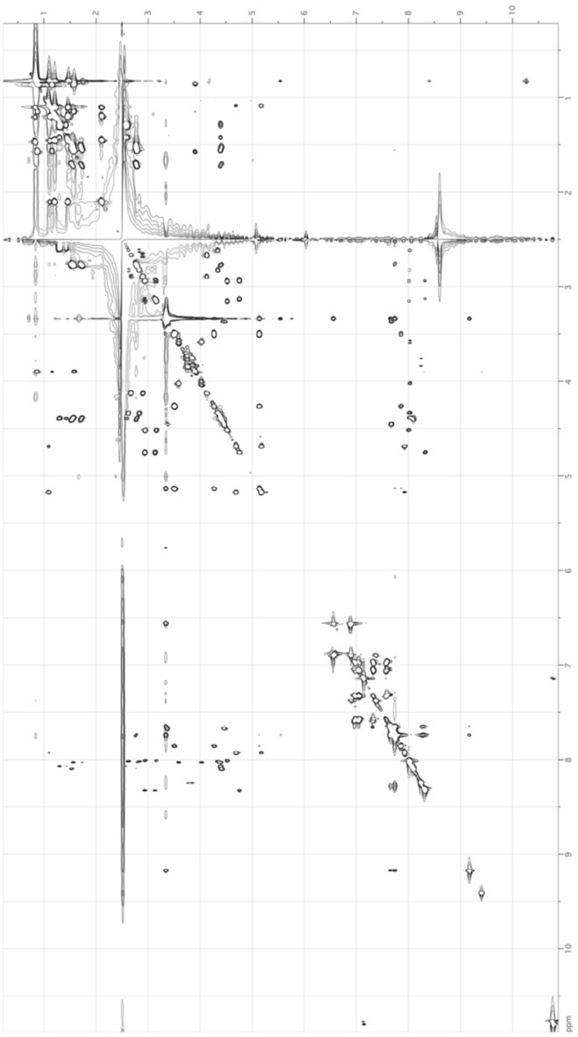

NOESY TOCSY

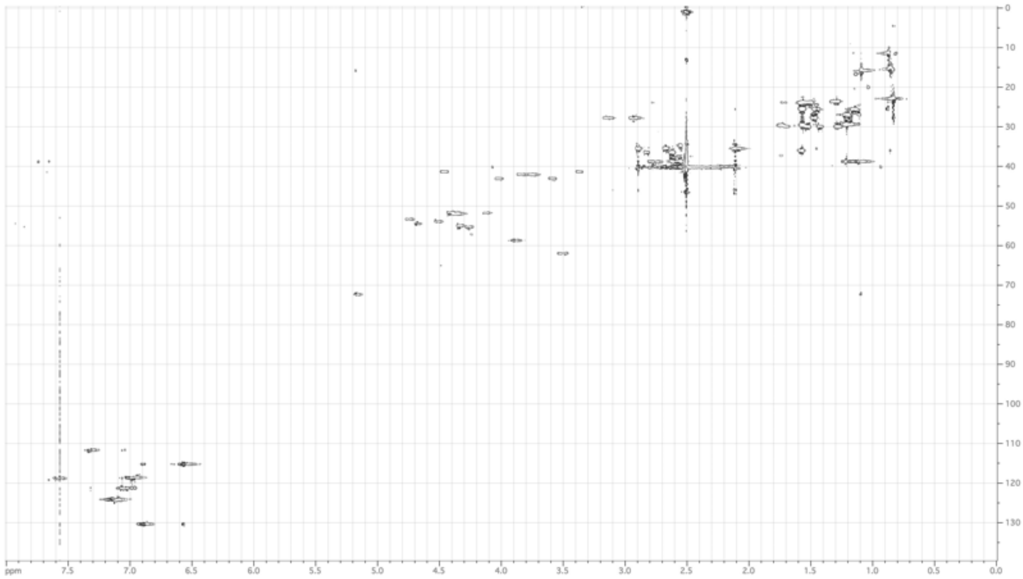

HSQC

## Ser9-Laterocidine (4)

| Residue | -NH | Hα | Hβ | Hγ | Hδ | Hε |
|---|---|---|---|---|---|---|
| D-Ser | 7.85 (1H, d, *J* = 7.2 Hz) | 4.26 (1H, q, *J* = 4.8 Hz) | 3.50 (m) | | | |
| D-Tyr2 | 8.00 (1H, m) | 4.34 (1H, m) | 2.83 (1H, dd, *J* = 14.3 & 3.8 Hz) & 2.62 (m) | 6.89 (2H, d, *J* = 8.3 Hz) & 6.56 (2H, d, *J* = 8.3 Hz) | | |
| D-Trp3 | 7.99 (1H, m) | 4.52 (1H, m) | 3.15 (1H, dd, *J* = 14.7 & 4.0 Hz) & 2.94 (m) | 10.79 (1H, s), 7.57 (2H, app t, *J* = 8.2 Hz), 7.33 (1H, d, *J* = 8.2 Hz), 7.14 (1H, br s), 7.07 (1H, t, *J* = 7.3 Hz), 6.98 (1H, t, *J* = 7.5 Hz) | | |
| D-Orn4 | 8.03 (1H, m) | 4.38 (1H, m) | 1.73 (1H, m), 1.56 (1H, m) | 1.56 (2H, m) | 2.78 (2H, m) | 7.69 (2H, m) |
| Orn5 | 8.08 (1H, d, *J* = 8.7 Hz) | 4.41 (1H, m) | 1.70 (1H, m), 1.52 (1H, m) | 1.52 (2H, m) | 2.77 (2H, m) | 7.69 (2H, m) |
| Gly6 | 8.23 (1H, m) | 3.82 (1H, dd, *J* = 17.0 & 5.9 Hz) & 3.76 (1H, dd, *J* = 17.4 & 4.1 Hz) | | | | |
| D-Orn7 | 8.08 (1H, m) | 4.40 (1H, m) | 1.39 (1H, m), 1.29 (1H, m) | 1.30 (2H, m) | 2.58 (2H, m) | 7.61 (2H, m) |
| Trp8 | 8.22 (1H, m) | 4.69 (1H, m) | 3.09 (1H, dd, *J* = 14.5 & 4.6 Hz) & 2.92 (m) | 10.76 (1H, s), 7.57 (2H, app t, *J* = 8.2 Hz), 7.31 (1H, d, *J* = 8.1 Hz), 7.10 (1H, br s), 7.04 (1H, t, *J* = 7.5 Hz), 6.95 (1H, t, *J* = 7.4 Hz) | | |
| Ser9 | 8.23 (1H, m) | 4.62 (1H, m) | 4.54 (1H, dd, *J* = 11.0 & 3.4 Hz) & 3.87 (1H, dd, *J* = 10.4 & 5.0 Hz) | | | |
| Ile10 | 8.31 (1H, d, *J* = 5.0 Hz) | 4.06 (1H, m) | 1.65 (1H, m) | 1.52 (1H, m), 1.14 (1H, m) & 0.87 (3H, m) | 0.86 (3H, m) | |
| Asn11 | 9.21 (1H, d, *J* = 6.2 Hz) | 4.17 (1H, m) | 2.87 (1H, dd, *J* = 16.0 & 3.8 Hz) & 2.62 (m) | | | |
| Gly12 | 7.98 (1H, m) | 4.04 (1H, m) & 3.50 (1H, m) | | | | |
| Gly13 | 7.60 (1H, m) | 4.39 (1H, m) & 3.56 (1H, br m) | | | | |
| Lipid | 2.11 (2H, m, O=C$\mathbf{CH_2}$), 1.46 (3H, O=C$CH_2\mathbf{CH_2}$ & $(CH_3)_2\mathbf{CH}$-), 1.20 (4H, $(CH_3)_2CHCH_2\mathbf{CH_2CH_2}$-), 1.11 (2H, $(CH_3)_2CH\mathbf{CH_2}$-), 0.83 (6H, $(\mathbf{CH_3})_2CHCH_2$-) | | | | | |

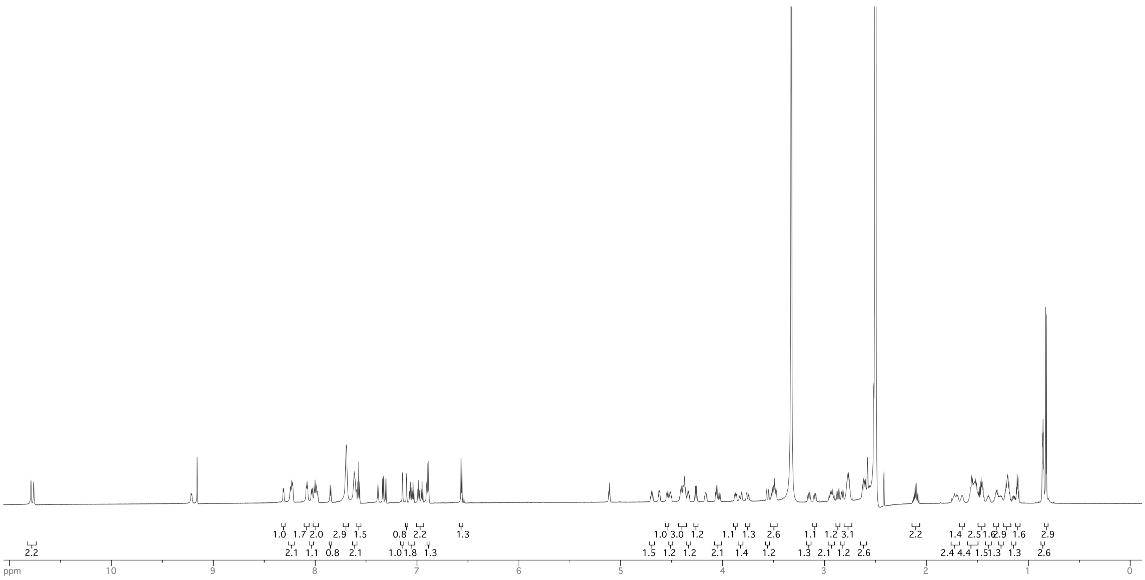


$^1$H NMR

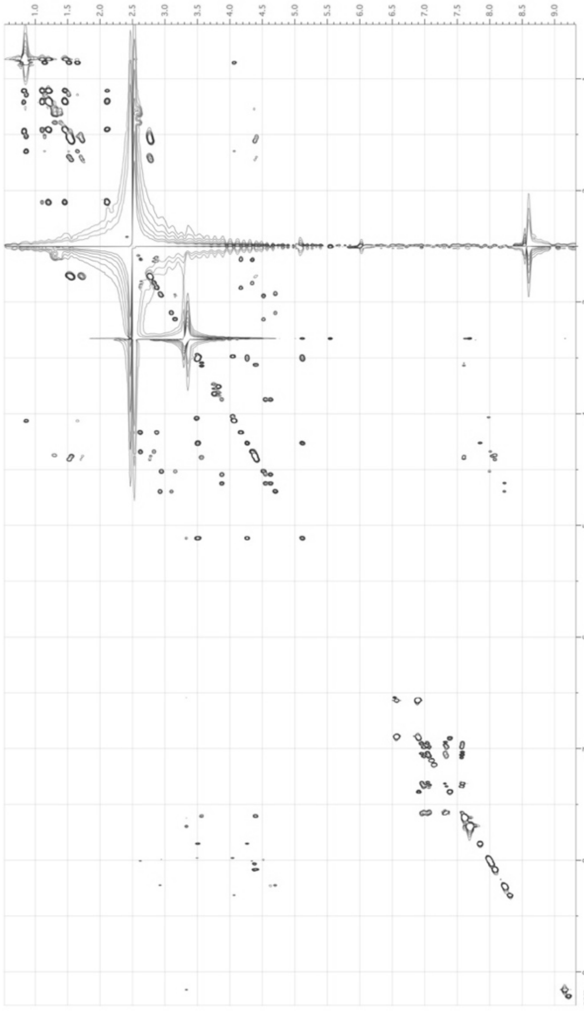

**NOESY**

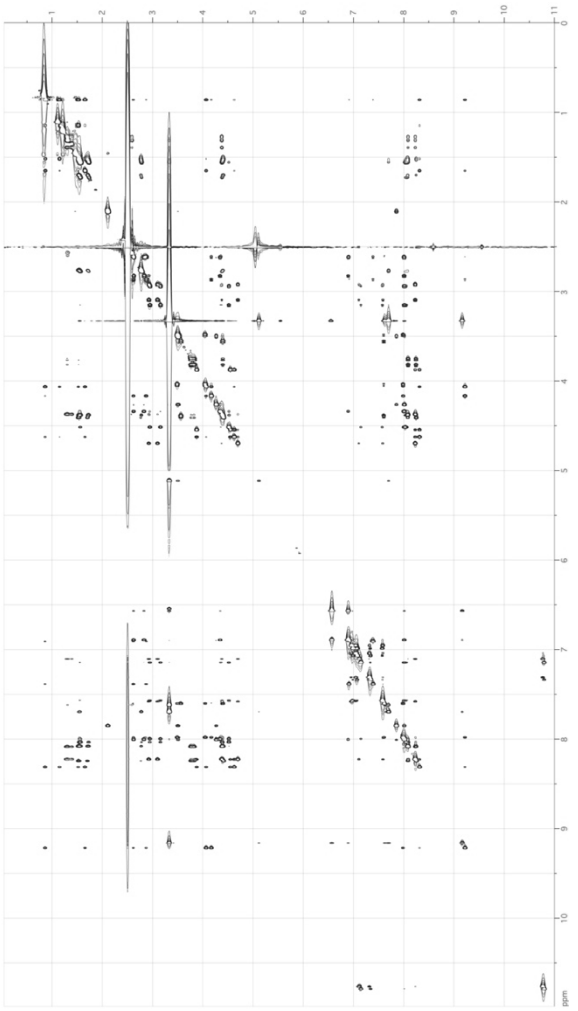

**TOCSY**

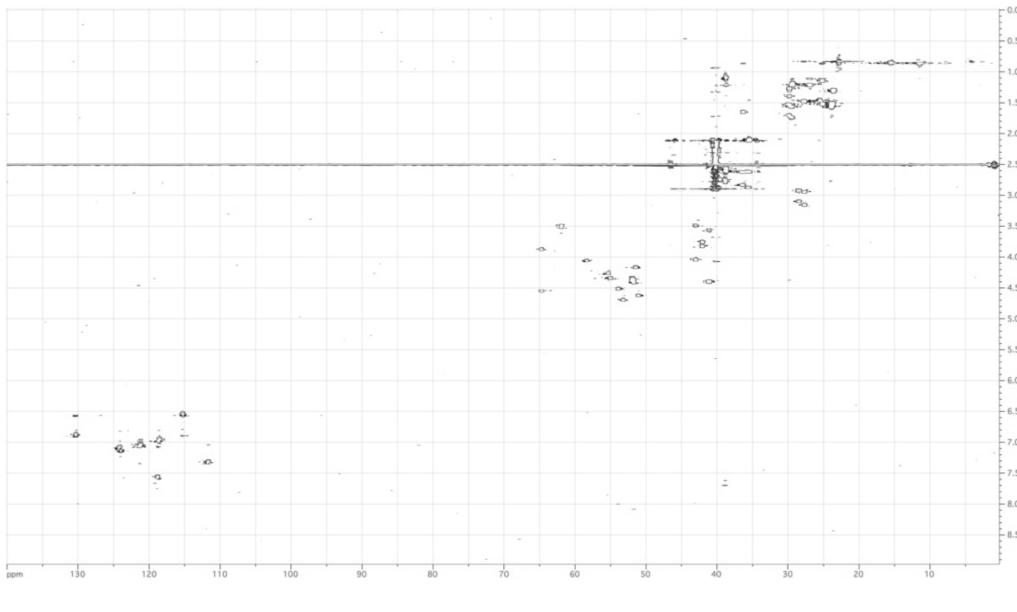


**HSQC**

## Dap9-Laterocidine (6)

| Residue | -NH | Hα | Hβ | Hγ | Hδ | Hε |
|---|---|---|---|---|---|---|
| D-Ser1 | 7.86 (1H, d, *J* = 7.3 Hz) | 4.26 (1H, q, *J* = 6.7 Hz) | 3.50 (1H, m) -OH: 5.14 (1H, br s) | | | |
| D-Tyr2 | 8.03 (1H, m) | 4.33 (1H, m) | 2.83 (1H, dd, *J* = 14.3, 4.2 Hz) & 2.62 (1H, m) | Aromatic: 6.89 (2H, d, *J* = 8.4 Hz) & 6.56 (2H, d, *J* = 8.3 Hz) & 9.18 (1H, s, -OH) | | |
| D-Trp3 | 8.02 (1H, m) | 4.51 (1H, m) | 3.15 (1H, dd, *J* = 14.5, 3.8 Hz) & 2.94 (1H, dd, *J* = 14.5, 9.3 Hz) | Indole: 10.80 (1H, s), 7.57 (1H, d, *J* = 7.9 Hz), 7.33 (1H, d, *J* = 8.1 Hz), 7.14 (1H, d, *J* = 1.7 Hz), 7.06 (1H, t, *J* = 7.5 Hz) & 6.98 (1H, t, *J* = 7.4 Hz) | | |
| D-Orn4 | 8.03 (1H, m) | 4.38 (1H, m) | 1.74 (2H, m) | 1.56 (2H, m) | 2.78 (2H, m) | 7.75 (2H, br) |
| Orn5 | 8.09 (1H, m) | 4.40 (1H, m) | 1.70 (2H, m) | 1.53 (2H, m) | 2.78 (2H, m) | 7.76 (2H, br) |
| Gly6 | 8.26 (1H, t, *J* = 5.1 Hz) | 3.79 (1H, dd, *J* = 16.8, 5.7 Hz) & 3.74 (1H, m) | | | | |
| D-Orn7 | 8.07 (1H, m) | 4.33 (1H, m) | 1.36 (1H, m) & 1.29 (1H, m) | 1.29 (2H, m) | 2.57 (2H, m) | 7.67 (2H, m) |
| Trp8 | 8.15 (1H, d, *J* = 8.3 Hz) | 4.65 (1H, m) | 3.09 (1H, dd, *J* = 14.1, 3.8 Hz) & 2.88 (1H, br m) | Indole: 10.78 (1H, s), 7.61 (1H, d, *J* = 7.9 Hz), 7.30 (1H, d, 8.1 Hz), 7.11 (1H, d, *J* = 1.8 Hz), 7.04 (1H, t, *J* = 7.4 Hz) & 6.96 (1H, t, *J* = 7.4 Hz) | | |
| Dap9 | 8.20 (1H, *J* = 7.0 Hz) | 4.29 (1H, br m) | 3.74 (1H, m) & 2.67 (1H, m) | -NH: 7.67 (1H, m) | | |
| Ile10 | 7.96 (1H, d, *J* = 9.1 Hz) | 4.51 (1H, m) | 1.62 (1H, m) | 1.05 (1H, br m), 1.40 (1H, m) & 0.85 (3H, m) | 0.81 (3H, m) | |
| Asn11 | 9.22 (1H, d, *J* = 6.2 Hz) | 4.16 (1H, m) | 2.92 (1H, m) & 2.56 (1H, m) | $-NH_2$: 7.39 (1H, br s) | | |
| Gly12 | 8.23 (1H, dd, *J* = 9.1, 2.5Hz) | 4.23 (1H, dd, *J* = 17.1, 9.6 Hz) & 3.28 (1H, br m) | | | | |
| Gly13 | 7.72 (1H, dd, *J* = 9.0, 3.5 Hz) | 4.15 (1H, m) & 3.39 (under $H_2O$) | | | | |
| Lipid | 2.10 (2H, m, O=C$C\mathbf{H_2}$-), 1.46 (3H, -$CH_2\mathbf{CH}(CH_3)_2$ & O=$CCH_2C\mathbf{H_2}$-), 1.20 (4H, m, -$\mathbf{CH_2}CH_2CH(CH_3)_2$ & O=$C(CH_2)_2C\mathbf{H_2}$-), 1.11 (2H, -$C\mathbf{H_2}CH(CH_3)_2$) & 0.83 (6H, -$CH_2CH(C\mathbf{H_3})_2$) | | | | | |

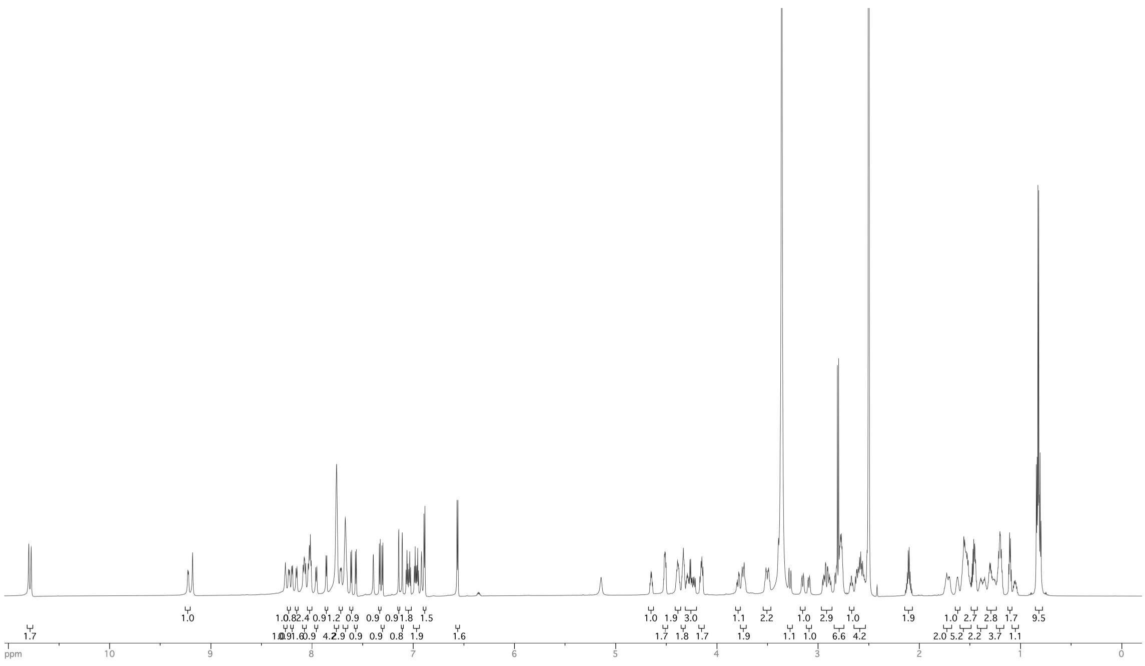


$^1$H NMR

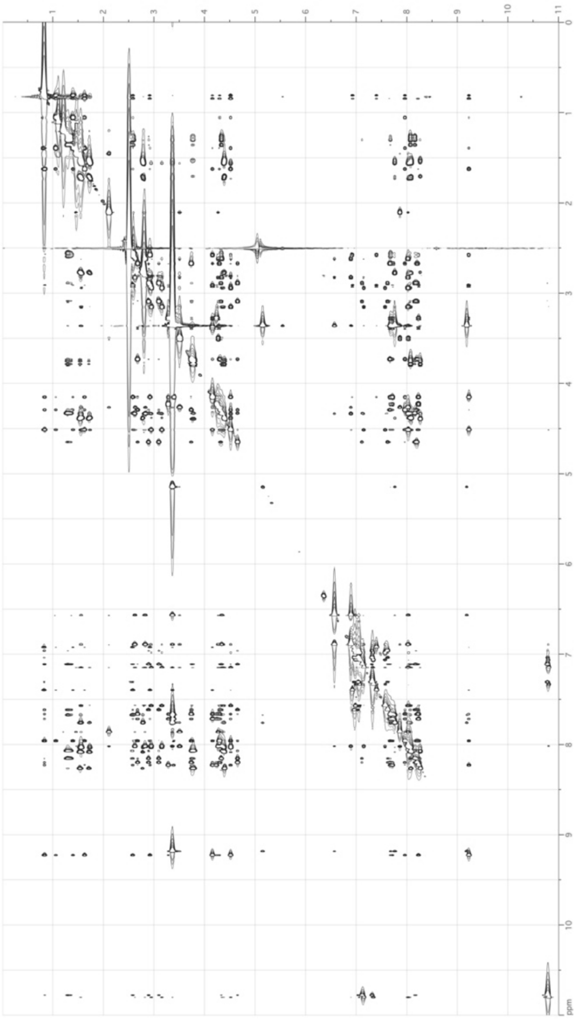

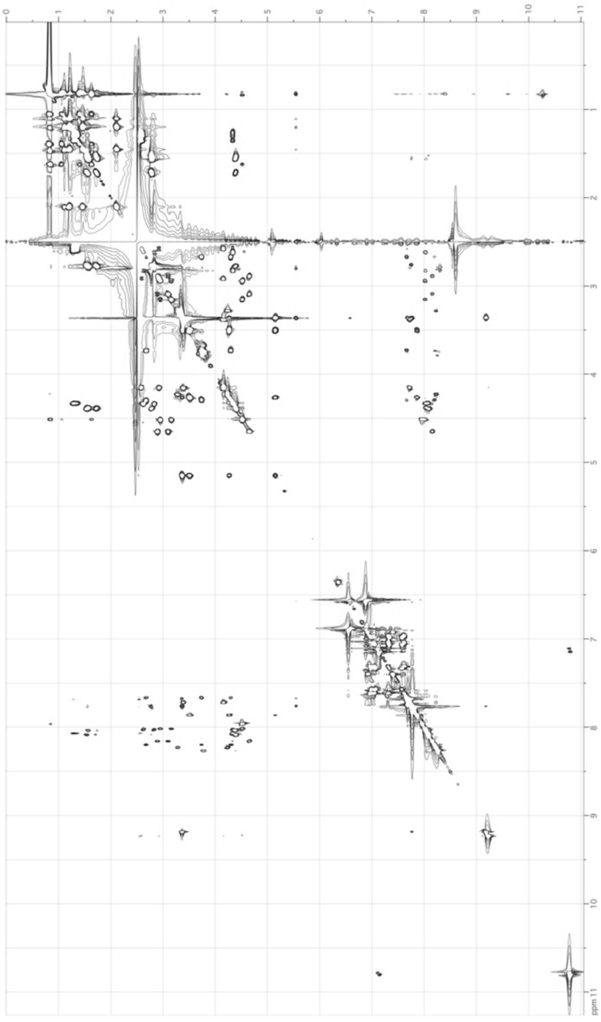

**NOESY** **TOCSY**

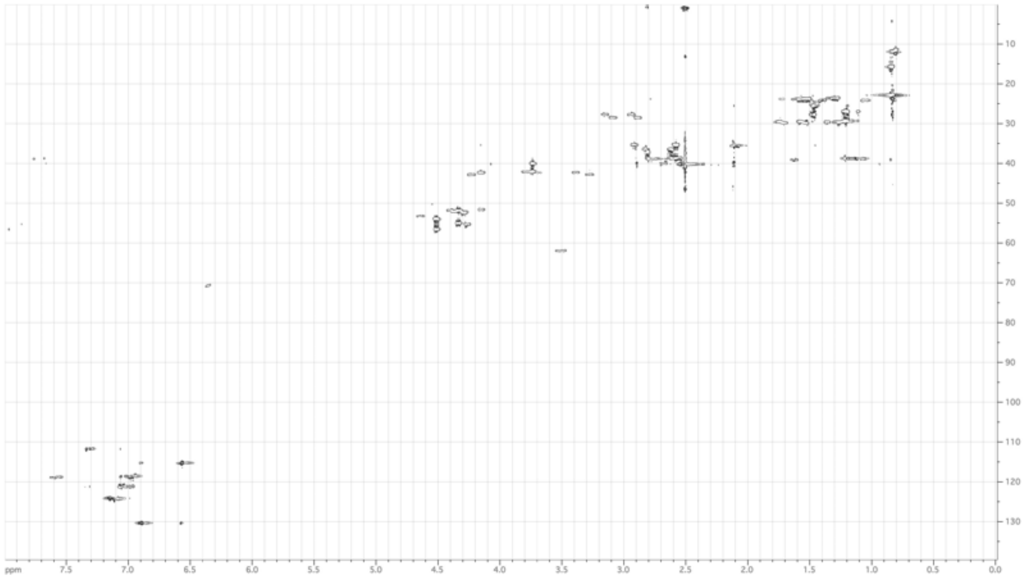


**HSQC**

## MeDap9-Laterocidine (8)

| Residue | -NH | Hα | Hβ | Hγ | Hδ | Hε |
|---|---|---|---|---|---|---|
| D-Ser1 | 7.85 (1H, d, *J* = 7.4 Hz) | 4.26 (1H, q, *J* = 6.7 Hz) | 3.50 (2H, m)<br>-OH: 5.12 (1H, *J* = 5.5 Hz) | | | |
| D-Tyr2 | 8.01 (1H, m) | 4.33 (1H, m) | 2.83 (1H, dd, *J* = 14.5, 4.2 Hz) & 2.62 (1H, m) | Aromatic: 6.89 (1H, d, *J* = 8.4 Hz) & 6.56 (1H, d, *J* = 8.3 Hz), 9.17 (1H, s, -OH) | | |
| D-Trp3 | 8.00 (1H, m) | 4.51 (1H, m) | 3.15 (1H, dd, *J* = 14.3, 3.6 Hz) & 2.93 (1H, br m) | Indole: 10.79 (1H, s), 7.57 (1H, d, *J* = 7.8 Hz), 7.33 (1H, d, *J* = 8.1 Hz), 7.14 (1H, br s), 7.06 (1H, t, *J* = 7.5 Hz) & 6.98 (1H, t, *J* = 7.4 Hz) | | |
| D-Orn4 | 8.03 (1H, d, *J* = 7.8 Hz) | 4.38 (1H, m) | 1.73 (1H, m) & 1.56 (1H, m) | 1.53 (2H, m) | 2.78 (2H, m) | 7.71 (2H, m) |
| Orn5 | 8.08 (1H, m) | 4.40 (1H, m) | 1.70 (1H, m) & 1.53 (1H, m) | 1.53 (2H, m) | 2.77 (2H, m) | 7.71 (2H, m) |
| Gly6 | 8.26 (1H, t, *J* = 5.0 Hz) | 3.82 (1H, dd, *J* = 17.2, 5.8 Hz) & 3.73 (1H, dd, *J* = 16.9, 4.8 Hz) | | | | |
| D-Orn7 | 8.13 (1H, d, *J* = 8.4 Hz) | 4.38 (1H, m) | 1.40 (1H, m) & 1.31 (1H, m) | 1.31 (2H, m) | 2.58 (2H, m) | 7.63 (2H, br m) |
| Trp8 | 8.20 (1H, d, *J* = 7.8 Hz) | 4.69 (1H, m) | 3.08 (1H, dd, *J* = 14.6 & 4.1 Hz) & 2.92 (1H, m) | Indole: 10.78 (1H, s), 7.60 (1H, d, *J* = 7.7 Hz), 7.31 (1H, d, 8.1 Hz), 7.12 (1H, d, *J* = 1.7 Hz), 7.04 (1H, t, *J* = 7.5 Hz) & 6.96 (1H, t, *J* = 7.3 Hz) | | |
| MeDap9 | 8.07 (1H, m) | 4.52 (1H, m) | 4.22 (1H, m) | 1.05 (2H, d, *J* = 7.0 Hz), 6.88 (1H, m, NH) | | |
| Ile10 | 7.91 (1H, d, *J* = 8.0 Hz) | 4.35 (1H, m) | 1.62 (1H, m) & 0.84 (1H, m) | 1.43 (1H, m), 1.08 (1H, m) & 0.84 (3H, m) | | |
| Asn11 | 9.25 (1H, d, *J* = 6.3 Hz) | 4.14 (1H, m) | 2.90 (1H, dd, *J* = 15.9 & 3.1 Hz) & 2.62 (1H, m) | | | |
| Gly12 | 8.35 (1H, br d, *J* = 7.6 Hz) | 4.12 (1H, m) & 3.41 (1H, dd, *J* = 17.1 & 3.1 Hz) | | | | |
| Gly13 | 7.73 (1H, m) | 4.17 (1H, dd, *J* = 16.9 & 9.3 Hz) & 3.36 (1H, under $H_2O$) | | | | |
| Lipid | 2.11 (2H, m, O=C$CH_2$-), 1.46 (3H, -$CH_2$**CH**$(CH_3)_2$ & O=C$CH_2$**$CH_2$**-), 1.20 (4H, -**$CH_2$**$CH_2CH(CH_3)_2$) & O=C$(CH_2)_2$**$CH_2$**-), 1.11 (2H, -**$CH_2$**$CH(CH_3)_2$) & 0.83 (6H, -$CH_2CH$(**$CH_3$**)$_2$ | | | | | |

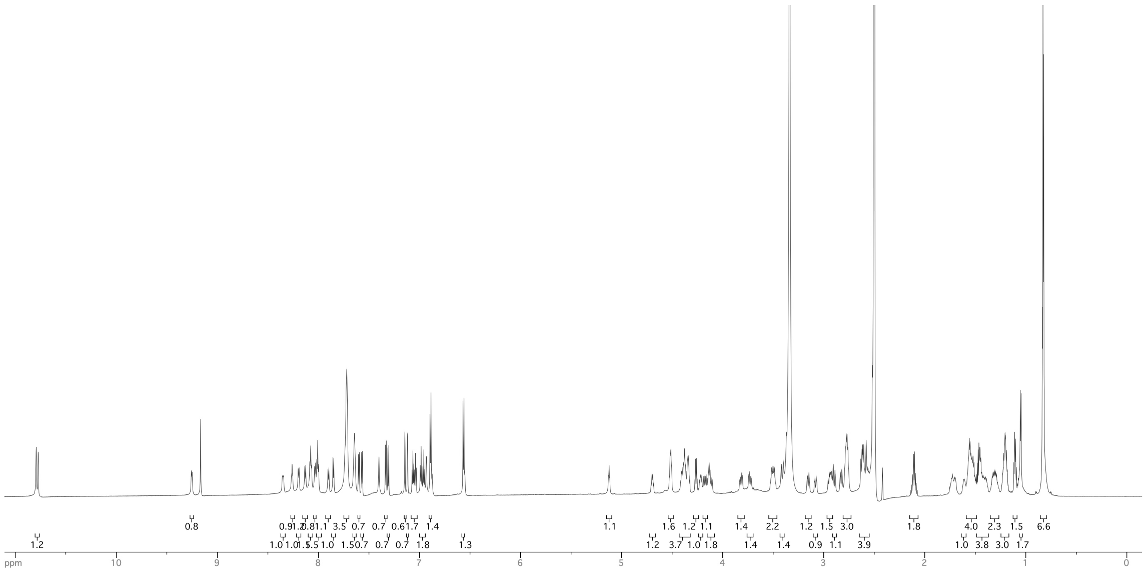

**$^1$H NMR**

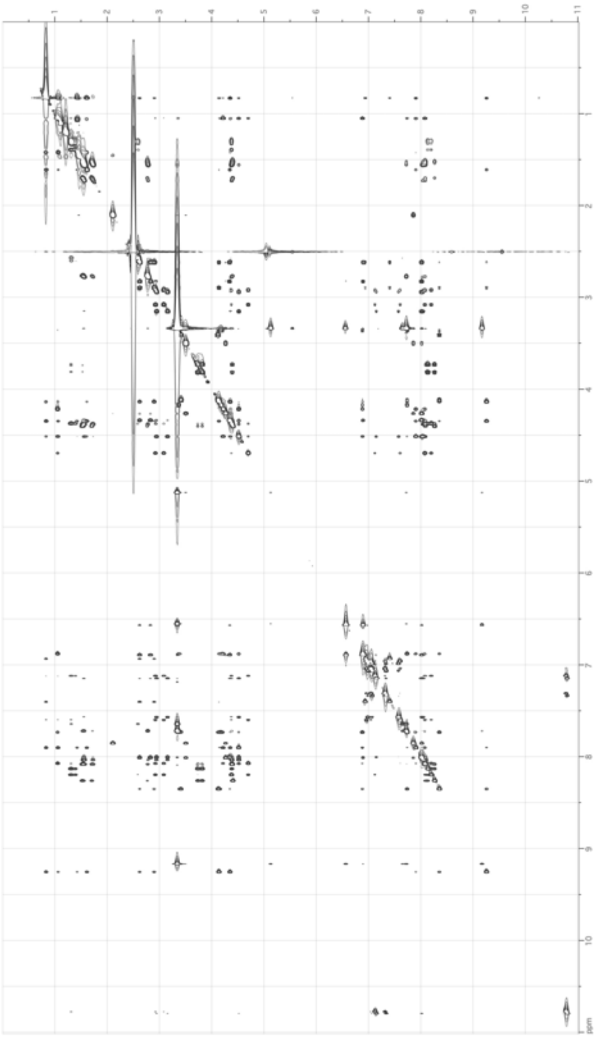

**NOESY**

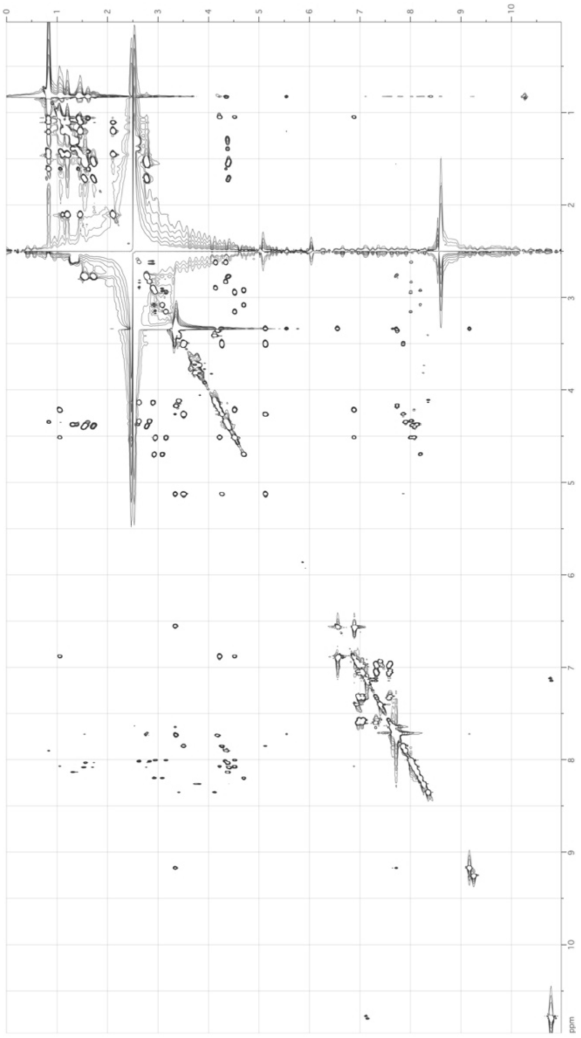

**TOCSY**

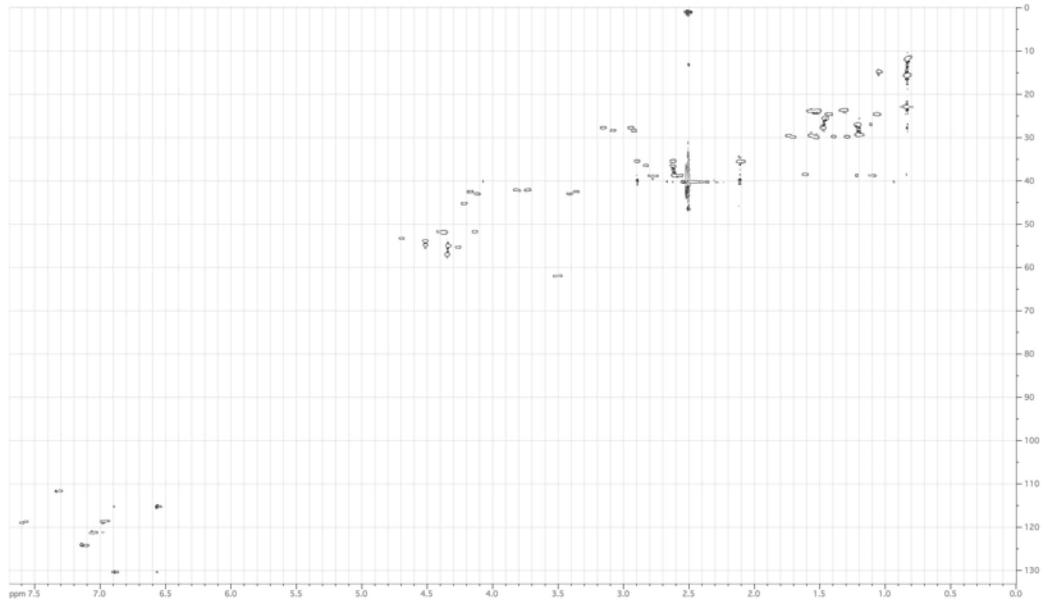

**HSQC**

## *References*


[1] S. Mukherjee, W. A. van der Donk. *J. Am. Chem. Soc.* **2014**, *136*, 10450-10459.

[2] J. Ge, L. Li, S. Q. Yao, *Chem. Commun.* **2011**, *47*, 10939-10941.

[3] F. Freire, J. D. Fisk, A. J. Peoples, M. Ivancic, I. A. Guzei, S. H. Gellman, *J. Am. Chem. Soc.* **2008**, *130*, 7839-7841.

[4] C. Chun, S. M. Lee, S. Y. Kim, H. K. Yang, S. C. Song, *Biomaterials* **2009**, *30*, 2349-2360.

[5] R. Moreira, G. Barnawi, D. Beriashvili, M. Palmer, S. D. Taylor, *Biorg. Med. Chem.* **2019**, *27*, 240-246.

[6] M. Gude, J. Ryf, P. D. White, *Lett. Pept. Sci.* **2002**, *9*, 203–206.
